# Transcriptome architecture and regulation at environmental transitions in flavobacteria: the case of an important fish pathogen

**DOI:** 10.1038/s43705-021-00029-9

**Published:** 2021-07-07

**Authors:** Cyprien Guérin, Bo-Hyung Lee, Benjamin Fradet, Erwin van Dijk, Bogdan Mirauta, Claude Thermes, Jean-François Bernardet, Francis Repoila, Eric Duchaud, Pierre Nicolas, Tatiana Rochat

**Affiliations:** 1 Université Paris-Saclay, INRAE, MaIAGE, 78350 Jouy-en-Josas, France; 2 Université Paris-Saclay, INRAE, UVSQ, VIM, 78350 Jouy-en-Josas, France; 3 Université Paris-Saclay, CEA, CNRS, Institute for Integrative Biology of the Cell (I2BC), 91198 Gif-sur-Yvette, France; 4 Sorbonne Université, CNRS, IBPS, Laboratoire de Biologie Computationnelle et Quantitative (LCQB), 75005 Paris, France; 5 Université Paris-Saclay, INRAE, AgroParisTech, Micalis Institute, 78350 Jouy-en-Josas, France

**Keywords:** Bacteria, Functional genomics

## Abstract

The family *Flavobacteriaceae* (phylum *Bacteroidetes*) is a major component of soil, marine and freshwater ecosystems. In this understudied family, *Flavobacterium psychrophilum* is a freshwater pathogen that infects salmonid fish worldwide, with critical environmental and economic impact. Here, we report an extensive transcriptome analysis that established the genome map of transcription start sites and transcribed regions, predicted alternative sigma factor regulons and regulatory RNAs, and documented gene expression profiles across 32 biological conditions mimicking the pathogen life cycle. The results link genes to environmental conditions and phenotypic traits and provide insights into gene regulation, highlighting similarities with better known bacteria and original characteristics linked to the phylogenetic position and the ecological niche of the bacterium. In particular, osmolarity appears as a signal for transition between free-living and within-host programs and expression patterns of secreted proteins shed light on probable virulence factors. Further investigations showed that a newly discovered sRNA widely conserved in the genus, Rfp18, is required for precise expression of proteases. By pointing proteins and regulatory elements probably involved in host–pathogen interactions, metabolic pathways, and molecular machineries, the results suggest many directions for future research; a website is made available to facilitate their use to fill knowledge gaps on flavobacteria.

## Introduction

Aquaculture is the fastest growing food-producing sector and now accounts for half of the human fish consumption in the world [1]. A primary concern of this expending sector is the control of infectious diseases, such as those caused by bacteria of the family *Flavobacteriaceae*. Flavobacteria are most frequently isolated from environmental sources such as soil, sediments and water, and represent an important component of their ecosystems by recycling complex organic matter [2]. Several species are aquatic pathogens [3], one of the most widely studied being *Flavobacterium psychrophilum*, a Gram-negative aerobic yellow-pigmented bacterium displaying gliding motility and growing between 4 and 23 °C [4].

*F. psychrophilum* primarily affects salmonids in freshwater environments. The conditions, known as rainbow trout fry syndrome and bacterial cold-water disease, are major sanitary issues for the fish farming activity worldwide. Control strategies rely exclusively on antibiotics and outbreaks have an impact on the environment and animal welfare [5, 6]. Infected fish present signs of tissue erosion, skin ulcerations, necrotic lesions and splenic hypertrophy. The bacterium is mainly found in skin lesions, dermal ulcers extending deeply into muscular tissues, and in lymphoid organs [7]. In rainbow trout fry, the disease often occurs as a septicemic form and mortality reaches 70% [5].

As members of the family *Flavobacteriaceae* and phylum *Bacteroidetes*, *F. psychrophilum* belongs to an understudied group whose transcription machinery and translation process substantially differs from most other bacteria: an unusual primary sigma factor binds atypical promoter sequences [8–10] and translation initiation relies on sequence properties differing from the Shine-Dalgarno [11, 12]. These unique expression signals result in limitations such as the inefficacy of genetic tools developed for other bacteria. Genome sequence analysis has generated relevant insights into *F. psychrophilum* epidemiology and evolution [13–17]. A number of infection-relevant phenotypic traits have also been reported. Bacterial cells are highly proteolytic [18, 19], cytotoxic for erythrocytes [20] and macrophages [21], attach to mucus [22], survive and potentially multiply inside phagocytes [23]. The Type IX secretion system (T9SS) is required for pathogenicity in rainbow trout [24]. Secreted proteases and iron acquisition systems are proposed to contribute to virulence [25–27]. Outside the host, water provides a natural dissemination medium for an aquatic pathogen provided that it can withstand a low nutrient environment before invading a host and *F. psychrophilum* survives long periods in freshwater while maintaining its virulence [28, 29]. Most genes associated with these phenotypic traits remain unknown and substantial efforts are needed to unravel the molecular factors involved in the pathogen life cycle.

During the last decade, advances in transcriptomics opened new routes to understand bacterial adaptation. Primary transcriptome mapping and condition-dependent transcriptome profiling proved to be particularly effective in providing genome-scale information allowing functional annotation of genomes, experimental discovery of regulatory elements such as promoters, transcription factors binding sites, *cis*- and *trans*-acting RNA elements [30–34]. Furthermore, by integrating expression data reflecting a large variety of living conditions one can observe how a bacterium reshapes its transcriptional program and understand some characteristics of transcriptional networks without the use of reverse genetics [35–37]. Nevertheless, transcriptomic studies are still scarce in the family *Flavobacteriaceae* [11, 38–41].

Here, we apply a combination of the above-mentioned transcriptomic approaches to address missing molecular knowledge on *F. psychrophilum* with a broad focus on understanding the timely and coordinated changes in gene expression needed to adapt to the diverse environmental conditions met by this aquatic pathogen during the three main stages of its life cycle: free-living in freshwater, on the fish surface, and inside the fish.

## Materials and methods

### Bacterial strains, plasmids, and growth conditions

This study used the wild-type strain *F. psychrophilum* OSU THCO2-90 isolated from the kidney of a Coho salmon in Oregon in 1990 which is a model strain for molecular genetics [16, 42]. Cultures were routinely performed in TYES broth at 18 °C (SI Appendix M1). Strains, plasmids and oligonucleotides are listed in SI Appendix T1 and T2. The transcriptional reporter plasmid pCP*Gm*^r^-P_*less*_-mCh and derivatives carrying *remF* promoter fragments were constructed using a pCP23-derivative vector carrying a gentamycin resistance marker as backbone [27] and the promoter activity was monitored using whole-cell fluorescence (SI Appendix M2). The *rfp18* deletion mutant was constructed using a pYT313-derivative plasmid [43] as described in SI Appendix M3. Proteolytic activity was quantified on azocasein substrate as previously described [24].

### Growth conditions

A total of 32 culture conditions were designed to cover environments encountered during *F. psychrophilum* life cycle with appropriate controls to allow meaningful analyses of differentially expressed genes (DEGs) (Table 1, SI Appendix T3). These conditions included different growth phases and controlled stresses or changes of the environmental parameters. Outside, surface and inside host environments were mimicked by incubating bacteria in freshwater into tanks (fish in rearing conditions) with or without fish and by exposure to fish mucus or fish plasma. Within-host osmotic conditions were imitated by 0.75% NaCl, a concentration supporting growth but close to the maximum that *F. psychrophilum* can tolerate [18].

**Table 1:** Overview of the 32 biological conditions analyzed for condition-dependent profiling.

^a^Three-color code indicating for which main stage of the pathogen life-cycle the condition is relevant: outside fish (blue), on fish surface (yellow) and inside fish (red).

^b^Conditions in which bacteria were in contact with rainbow trout-derived compounds are highlighted.

^c^Controlled laboratory conditions are highlighted.

^d^Italicized labels indicate conditions used as references in pairwise differential analyses to identify up- and down-regulated genes (DEG analysis described in Table S5).

^e^A more detailed description of the 32 culture conditions is available in Table S1.

### RNA extraction, libraries preparation, and RNA-sequencing

Two distinct sets of RNA samples were used for RNA-Seq (18 samples pooled) and for expression profiling (64 individual samples corresponding to 32 conditions in duplicate based in independent cultures; Table S1). Total RNA extractions were performed using the hot phenol method as previously described [44]. DNase-treated RNA extracts were used to prepare an equimolar 18-condition RNA pool that served for 5′-end and global RNA-Seq libraries preparation (SI Appendix M4). The sequencing was performed on Illumina HiSeq platform (single-end, 50 bp). Reads were aligned to the complete genome sequence [16] as described in SI Appendix M5.

### Determination of putative TSSs and classification of newly defined TRs

Identification of putative TSSs was based on the exact starting positions of uniquely mapped reads of the 5′-end RNA-Seq library (SI Appendix M6). Genomic segments transcribed outside genome annotation and RFAM predictions [45] were delineated based on expression-level reconstructed from global RNA-Seq reads [46] as well as predicted intrinsic terminators and high-confidence TSSs (SI Appendix M7). After expert curation, a total of 1511 TRs were classified into RNA categories according to their transcriptional context (SI Appendix D1).

### Condition-dependent transcriptomics

Design of SurePrint G3 Custom GE 8x60K microarrays (Agilent technologies), strand-specific cDNA synthesis, hybridization procedures and data processing are described in SI Appendix M8.

### Computational analysis of promoter sequences and newly defined TRs

Promoter sequences and promoter activities across samples were analyzed together to identify sigma factor motifs using the TreeMM algorithm [37] with some modifications (SI Appendix M9). Subsets of new TRs were analyzed for phylogenetic conservation, RNA secondary folding and mRNA-sRNA interactions (SI Appendix M10).

### Real time qPCR gene expression analysis

qPCR was performed on CFX system following manufacturer’s instruction (BIO-RAD) and expression level was normalized by geometric mean of 2 reference genes (SI Appendix M11).

### Online data display

The website https://fpeb.migale.inrae.fr embeds Jbrowse (version 1.12.3) [47] and a SequenceServer (version 1.0.11) to allow blast searches [48]. Browsing is possible along the chromosome, down to the level of read coverage and hybridization signal of individual probes, and across the expression space based on correlation between genes. The interface allows online extraction of specific lists of features (new RNAs, TSSs, gene clusters, DEGs), export of figures, access to genomic coordinates and nucleotide sequence for all features.

## Results and discussion

### Combining experimental and *in silico* strategies to unravel transcriptome architecture

**Fig. 1:**
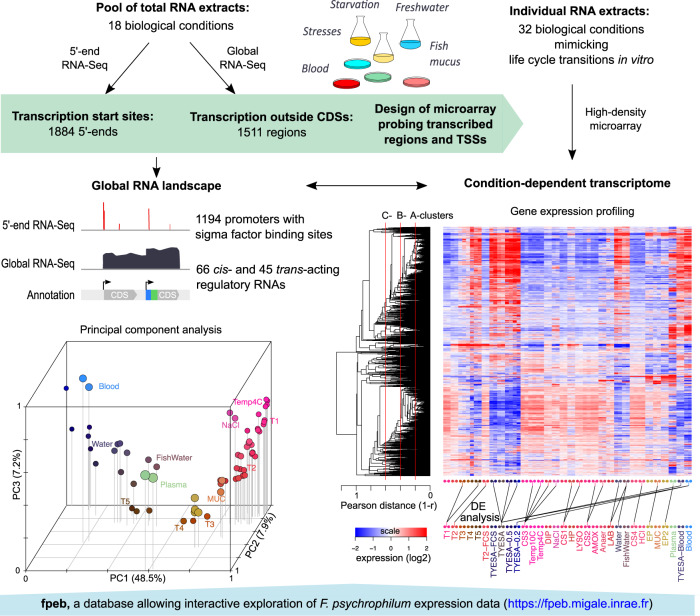
Global assessment of the RNA landscape and condition-dependent transcriptome. Upper panel: Overview of the strategy that combines the advantages of RNA-Seq and microarrays: detection of low-abundance transcripts and bp-level resolution; accurate and cost-effective quantification of transcript levels without PCR amplification biases. Lower left panel: 3D representation of the 64 samples with coordinates on the PC axes (Principal Component Analysis); lower right panel: Heatmap representation of gene-centered expression profiles and pairwise comparisons of samples identifying condition-relevant DEGs. Labels of samples are listed in Table 1 (details in Table S1). The average-link hierarchical tree shown on the left margin of the heatmap was built on pairwise Pearson distance (1 – *r*) between gene expression profiles and served to define co-expression clusters. The statistical significance of the three reference correlation levels (*r* = 0.8, 0.6, and 0.4) used to define A-, B- and C-clusters and the corresponding distributions of cluster sizes are illustrated in SI Appendix D2.

Experimental and computational methodologies were combined to reconstruct the transcriptional landscape of *F. psychrophilum* OSU THCO2-90 (Fig. 1). Transcription start sites (TSSs) and transcribed regions (TRs) outside CDSs were identified by 5′-end and global RNA-Seq (Tables S2 and S3). Transcriptional responses were analyzed across 32 biological conditions representative of *F. psychrophilum* living environments (Table 1). Genes were partitioned into clusters according to the hierarchical clustering tree of their expression profiles (Fig. 1, SI Appendix D2). Results presented here can be explored in details at https://fpeb.migale.inrae.fr.

Principal Component Analysis of the transcriptomes revealed highly coordinated changes in gene expression with growth phase (PC1) and between key environments: fish plasma (PC2), growth on blood (PC3) and freshwater (PC4; Fig. 1, SI Appendix D2). Analysis revealed that 57.4% of the 2410 CDSs were highly expressed (in the upper quartile of expression level) in at least one sample of the dataset. Only 5.8% (136) were highly expressed in all samples including typical housekeeping genes encoding ribosomal proteins and carbon metabolism enzymes, but also those encoding the TonB-ExbBD system, several outer membrane proteins, the T9SS core components and gliding motility proteins (Table S4). Only 4.4% (103) of the CDSs, mostly of unknown function, showed low expression in all samples. High congruence between biological replicates allowed to identify differentially expressed genes between conditions: 86% CDSs were found differentially expressed in at least one comparison (SI Appendix T4, Table S5). Taken together, these numbers indicate good coverage of the expression space.

### Characterization of promoters and alternative sigma factor regulons

The 1884 genomic positions identified as putative primary 5′-ends by 5′-end RNA-Seq (SI Appendix D3) were further analyzed to establish a list of high-confidence TSSs and to identify high-level transcriptional regulation by sigma factors, the transient subunits of bacterial RNA polymerase responsible for recognition of promoter sequences. Besides the primary sigma factor, hereafter named *σ*^A^, the *F. psychrophilum* genome contains 8 alternative sigma factors: 1 *σ*^54^ and 7 extracytoplasmic function (*σ*^ECF^) sigma factors, some of them induced in specific conditions such as plasma exposure and high osmotic pressure (SI Appendix D4). The dataset was analyzed for *de novo* identification of sigma factor binding sites by combining information from DNA sequences, 5′-end positions and condition-dependent expression profiles [37]. Sites were predicted for 1194 (63.4%) of the initial list of putative TSSs and their genomic contexts suggest a good sensitivity of *in silico* detection of promoters and good specificity of experimental mapping of TSSs (SI Appendix D3). These high-confidence TSSs preceded 890 CDSs (38% of the CDSs annotated in the genome) which gives a lower bound on the number of distinct mRNA transcription units, each often encompassing several adjacent codirectional CDSs (polycistronic mRNAs).

The length of 5′ untranslated regions (5′ UTRs), computed as the distance between a TSS and the predicted start codon, was examined for these 890 CDSs. The average and median lengths were 65 and 24 bp, which is close to those reported for *Bacteroides thetaiotaomicron* (52 and 32 bp, respectively) and for bacteria in other phyla such as *Escherichia coli* or *Bacillus subtilis* [49–51]. Leaderless mRNAs might have been more frequent given the absence of Shine-Dalgarno sequences, but they accounted for only 5.5% of these mRNAs, which is consistent with a previous observation in *Flavobacterium johnsoniae* [11].

**Fig. 2:**
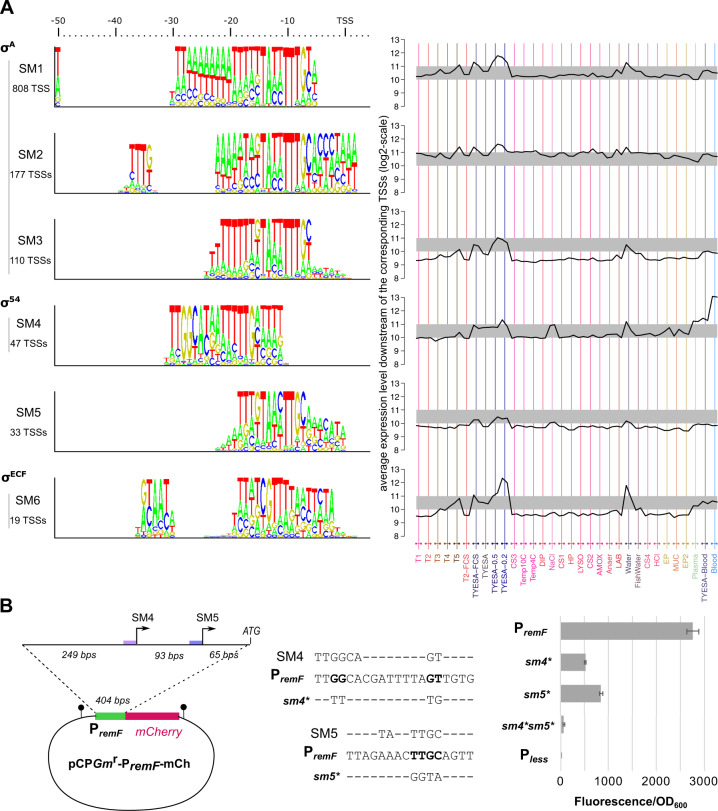
Sigma factor binding sites. **A** Logo representation of the 6 sigma factor binding motifs identified *in silico* and average expression downstream the corresponding TSSs across conditions. Expression levels have been normalized by applying the transformation used for quantile-normalization of CDS expression levels. **B** Mutagenesis of sigma factor binding sites of the *remFG-sprCDBF* operon. Schematic representation of transcriptional fusions and mutagenesis of highly conserved nucleotides in SM4 and SM5 motifs. Promoter activity was measured using whole-cell fluorescence of *F. psychrophilum* strains carrying the mCherry reporter plasmid. Values represent the mean and standard deviation of three independent experiments.

The *de novo* prediction algorithm associated TSSs to 6 distinct sigma factor binding site motifs, numbered SM1-6 according to their number of occurrences (Fig. 2A). The 3 most abundant (SM1-3) consist of variations around the TAnnTTTG consensus of the −7 box recognized by *σ*^A^. Subtle differences between these *σ*^A^ motifs may have a role in the regulation of promoter activity (SI Appendix D5). SM4-6 differ from the *σ*^A^ consensus and collectively represent 7% of the high-confidence TSSs, most likely under the control of alternative sigma factors.

Motif SM4 displays the typical −24/−12 elements of *σ*^54^ controlled promoters [52], indicating that downstream genes are part of the *σ*^54^ regulon of *F. psychrophilum*. Transcription level downstream SM4 promoters showed a strong induction during growth on blood agar and, to some extent, under high osmotic pressure, plasma exposure and into freshwater (Fig. 2A, SI Appendix D5). The transcribed genes mainly belonged to two co-expression clusters that encode functions related to quality control of proteins and envelope stress response, gliding motility as well as several exported proteins of unknown function (Table 2A). Across bacterial species, *σ*^54^ factors are reported to control pathways as diverse as nitrogen assimilation, flagellar biosynthesis or carbon uptake but a common theme is to control processes related to physical interaction with the environment [53]. This is consistent with our findings in *F. psychrophilum*. Transcriptional activation by *σ*^54^ strictly depends on enhancer-binding activators. Since three proteins containing a *σ*^54^ interaction domain (PF00158) were predicted in the genome, co-expression clusters within SM4 promoters might correspond to distinct activators sensing different environmental signals.

**Table 2:** Promoters with predicted alternative sigma factor binding sites.

TSS position	Downstream genes (locustag)	Downstream gene products	Gene cluster
**A. Selection of SM4 promoters**			
867211	THC0290_0729 THC0290_0728	10 kDa chaperonin GroES 60 kDa chaperonin GroEL	B254
1168709	THC0290_0994	ATP-dependent endopeptidase Lon (S16 family)	B254
1654192	THC0290_1425	Chaperone protein DnaK	B254
1798337	THC0290_1544 THC0290_1545	Chaperone protein GrpE Chaperone protein DnaJ	B254
2117454	THC0290_1828	Chaperone protein HtpG	B254
753817	THC0290_0618	Outer membrane chaperone Skp (OmpH)	B258
1110250	THC0290_0942	ClpB, ATPase with chaperone activity	B415
2425654	THC0290_2103	Rhomboid family intramembrane serine protease	B415
25239	THC0290_0021 to THC0290_0026	Gliding motility operon *remF-remG-sprC-sprD-sprB-sprF*	B419
356002	THC0290_0297 THC0290_0298	Protein of unknown function 1-deoxy-D-xylulose-5-phosphate synthase Dxs (isoprenoid biosynthesis)	B419
710561	THC0290_0583	Uncharacterized N-acetyltransferase YitI	B419
840245	THC0290_N_0510	Putative regulatory RNA Rfp36	B419
966765	THC0290_0811	Putative muramidase	B419
1042927	THC0290_0885	Probable lipoprotein precursor	B419
1655528	THC0290_1426	Protein of unknown function precursor	B419
1806311	THC0290_1552	Orotate phosphoribosyltransferase PyrE (pyrimidine metabolism)	B419
2172256	THC0290_1870	Protein of unknown function precursor	B419
2618622	THC0290_2263	Thioredoxin family protein	B419
490781	THC0290_0402	Outer membrane protein precursor, AsmA family	
552525	THC0290_0455	Protein of unknown function with C-type lysozyme inhibitor family domain	
2117336	THC0290_1827 THC0290_1826	Probable lipoprotein Probable lipoprotein with OmpA-like domain	
**B. Selection of SM5 promoters**			
3491	THC0290_0003	LysE-type exporter protein	
25333	THC0290_0021 to THC0290_0026	Gliding motility operon *remF-remG-sprC-sprD-sprB-sprF*	
479610	THC0290_0394	Asp/Glu-specific dipeptidyl-peptidase, Dpp11	
870544	THC0290_0733 THC0290_0732	Probable sigma-54-dependent transcriptional regulator Lipopolysaccharide assembly protein LptE	
999712	THC0290_0839 THC0290_0838 THC0290_0837	Protein of unknown function dTDP-4-amino-4,6-dideoxygalactose transaminase Oligosaccharide translocase WzxE	
1187638	THC0290_1009	Probable transmembrane protein of unknown function	
1582507	THC0290_1371	Putative glutamine cyclotransferase	
1716045	THC0290_1475	Glycosyl transferase (group 1 family)	
2046887	THC0290_1761	Probable lipoprotein precursor	
2501832	THC0290_2166	Putative enzyme with P-loop containing nucleotide triphosphate hydrolase domain	
2676614	THC0290_2321	Metallo-beta-lactamase superfamily protein	
2773735	THC0290_2400	RmlD, dTDP-4-dehydrorhamnose reductase (synthesis of dTDP-L-rhamnose)	
**C. Selection of SM6 promoters**			
9250	THC0290_0009	Putative inner membrane protein (Band 7 family)	
49622	THC0290_0031	Protein of unknown function YceA, rhodanese-related sulfurtransferase	
182564	THC0290_N_0107	Putative regulatory RNA Rfp11	
758025	THC0290_0621	Outer membrane protein; Homolog in *P. gingivalis* (PG0189) interacts with the PorM/PorN complex of T9SS	
882660	THC0290_0743	GldN, core component of the T9SS machinery	
1229139	THC0290_1050	ATPase component of a probable ABC-type transporter	
1750688	THC0290_1507	Putative inner membrane protein (Band 7 family)	
1954718	THC0290_1681	TPR-domain containing protein	
2057098	THC0290_1774 THC0290_1773	ECF-type sigma factor Anti-sigma factor	
2382309	THC0290_2050	RmlB, dTDP-glucose 4,6-dehydratase (synthesis of dTDP-L-rhamnose)	
2473290	THC0290_2146	PorU, C-terminal signal peptidase of the T9SS	

A full list of promoters is available in Table S2, see also the genome browser at https://fpeb.migale.inrae.fr/tss_annotation.html.

Motif SM5 is characterized by a TAnnTTGY box at the same position (−12 to −5 bp) than the *σ*^A^ consensus TAnnTTTG. A striking similarity is the presence of highly conserved elements TA and TTG, but the distance between them is 1 bp shorter in SM5. Accordingly, guanidine at −6 and pyrimidine at −5 are specific of SM5 promoters. Transcribed genes are related to LPS biosynthesis, amino-acids scavenging and gliding motility (Table 2B, SI Appendix D5).

Motif SM6 contains a conserved CGT box in the −10 region (Fig. 2A) that strongly suggests recognition by a σ^ECF^-type sigma factor [54]. Transcription downstream SM6 promoters was characterized by a strong induction under low nutrients conditions such as freshwater or growth on very low-nutrient agar. Transcribed genes encode several components of the T9SS (Table 2C, SI Appendix D5), which is reminiscent of the control of T9SS genes by a σ^ECF^ reported in *Porphyromonas gingivalis*, a non-motile *Bacteroidetes* [55, 56]. Two transmembrane proteins with conserved “Band-7” domain were also transcribed from SM6 promoters. In prokaryotes, this family contains scaffold proteins called flotillins that are associated with functional membrane microdomains. Though their function is not fully understood, flotillins can promote protein complexes assembly [57]. The presence of one anti-sigma/σ^ECF^ factor system among the SM6 associated genes suggests that this system regulates SM6 promoters. Nevertheless, the biological conditions of the expression of several σ^ECF^ factors overlapped (SI Appendix D4), which also suggests functional redundancy and partially overlapping regulons, as observed in other bacteria [58]. SM6 could thus correspond to promoters recognized by several sigma factors, as observed for computationally inferred σ^ECF^ binding sites in *Bacillus subtilis* [37]. Furthermore, the conditions considered in this study might not have allowed full activation of all σ^ECF^-type factors. Consequently, the relative contribution of the predicted seven σ^ECF^-type factors in the control of SM6 promoters remains to be determined, and other σ^ECF^-controlled TSSs may remain to be discovered.

Experimental validation of the inferred regulatory motifs was performed on a case of particular interest: the *remFG-sprCDBF* operon. This operon is conserved in several *Flavobacterium* species and encodes the cell surface adhesin SprB known in *F. johnsoniae* to mediate bacterial cell attachment and propulsion over surfaces, as well as SprF which is required for secretion of SprB by the T9SS [59, 60]. Two promoters carrying the SM4 (σ^54^) and SM5 sigma factor binding sites were predicted upstream the operon. By constructing a reporter plasmid for *F. psychrophilum* (SI Appendix D6) and promoter mutagenesis, we confirmed the contribution of these two regulatory elements to *remFG-sprCDBF* transcription and the importance of highly conserved nucleotides of the sigma factor motifs identified *in silico* (Fig. 2B).

This whole analysis of mRNA 5′-ends and transcription initiation signals provides valuable information for future molecular studies on flavobacteria and brings out several key processes that are tightly controlled by alternative sigma factors in *F. psychrophilum*.

### The noncoding RNA repertoire of *F. psychrophilum*

A repertoire of 1511 regions transcribed outside annotated CDSs or ubiquitous RNAs were classified according to their transcriptional context and encompassed regulatory RNA candidates as well as signatures of pervasive transcription. The condition-dependent transcriptome dataset confirmed expression of at least 87% of the TRs (Table 3; SI Appendix D1).

**Table 3:** Summary of the 1511 regions transcribed outside annotated CDSs.

Category of TRs^a^	Total number (#)	Median length (bp)	TRs of length <150 bp			TRs of length ≥ 150 bp			Expression^d^
			#	CDS^b^	AS^c^	#	CDS^b^	AS^c^	
5′	231	80	182	0	0	49	4	4	95%
3′	586	108	333	0	21	253	14	166	79%
3′ PT	67	314	13	0	4	54	5	35	78%
Intra	542	99	374	2	0	168	10	4	91%
Indep	85	176	40	0	14	45	4	26	94%
All	1511	105	942	2	39	569	37	235	87%

^a^Classification by transcriptional context: [5′] = in 5′ of a CDS; [3′] = in 3′ of a CDS; [3′-PT] = resulting from partial termination of transcription (*i.e*. downstream a 3′ region terminated by a predicted intrinsic terminator); [intra] = between two CDSs, part of a polycistronic mRNA; and [indep] = independent of previously annotated features.

^b^Number of TRs with predicted CDS.

^c^Number of TRs in antisense of CDSs or new TRs.

^d^Percentage of TRs whose expression level is above the lower quartile of CDS expression in at least one sample.

A detailed description of the 1511 TRs and all features from genome annotation is available in Table S3, see also the *fpeb* website at https://fpeb.migale.inrae.fr/structural_annotation.html.

#### Antisense RNAs

Overall, 287 TRs overlapping the antisense strand of 281 CDSs were detected. This represents 12% of the total number of CDSs and very diverse biological functions (Table S6). Antisense RNAs (asRNAs) originated mostly from imperfect termination of transcription and from transcription initiation at non-canonical locations, as exemplified by the high proportion of asRNAs in 3′, 3′PT, and indep TR categories (Table 3, SI Appendix D7). Such antisense transcription patterns have been reported in a wide range of bacterial species and is referred to as pervasive transcription. While the general role of bacterial asRNAs is still an open question, many cases of regulatory functions involving mechanisms as diverse as transcriptional interference, modulation of mRNA stability, and translation inhibition have been documented (reviewed in [61]). In *Bacteroides fragilis*, asRNAs are reported for 15 polysaccharides utilization loci (PULs), and some negatively modulate the expression of their cognate PUL [62]. The list of asRNAs reported here might be a starting point for similar functional studies in flavobacteria.

#### 5′ cis-regulatory RNAs

5′ *cis-*regulatory RNAs usually adopt complex secondary structures that are essential to sense a particular signal (e.g., small molecule, temperature, ribosome, or protein binding). We confirmed the expression of 5 predictions made by scanning the genome for known *cis*-regulatory RNA families using RFAM [45]. For each of these 5′ regulatory RNAs, there was a clear coherence between the predicted sensed signal (e.g., cobalamin, thiamine) and the functions and expression profiles of the regulated genes (SI Appendix D8). In particular, a cobalamin riboswitch (control by cobalamin availability), was identified upstream of an unknown TonB-dependent transporter (TBDT, THC0290_1776) located in the vicinity of the *cob* genes encoding the adenosyl cobalamin biosynthesis proteins. Interestingly, enzymes catalyzing the first steps of the pathway are missing in *F. psychrophilum* and supply of this cofactor likely relies on the scavenging of cobamide precursors or vitamin B12 itself. Upregulation of this TBDT gene in fish plasma suggests retrieval of this nutrient from the host.

To go beyond confirmation of known 5′ *cis*-regulatory RNAs, secondary RNA structures were examined. This resulted in a list of 64 structured 5′ TRs that most likely play a regulatory role (Table S7, SI Appendix D9). Strong clues for a leader peptide attenuation mechanism by which translation of a short peptide regulates transcriptional elongation [63] were found upstream operons encoding amino-acids biosynthesis pathways. Structured 5′UTRs were identified for mRNAs related to a large variety of functions such as aminoacyl-tRNA-synthetases, carbon metabolism enzymes and peptidases. Several 5′ TRs coincided with hits of Flavo-1, a computationally inferred RNA motif widespread in *Flavobacteriaceae* [64]. Nevertheless, this did not give real clues on the function of this structural element since Flavo-1 hits seemed indistinctly located in sense or antisense of the TRs and many were not in TRs. A number of structured 5′ elements identified for the first time in this study were conserved outside the species. Some are present in other genera of the family *Flavobacteriaceae* (*e.g*. the 5′ elements upstream of *pheA*, *hisG*, *ftsY*, *acsA*, *alaS*, or *rimP*). Without hits in the RFAM database, they probably represent original *cis*-regulatory elements whose study may be of great interest.

#### Small regulatory RNAs

**Fig. 3:**
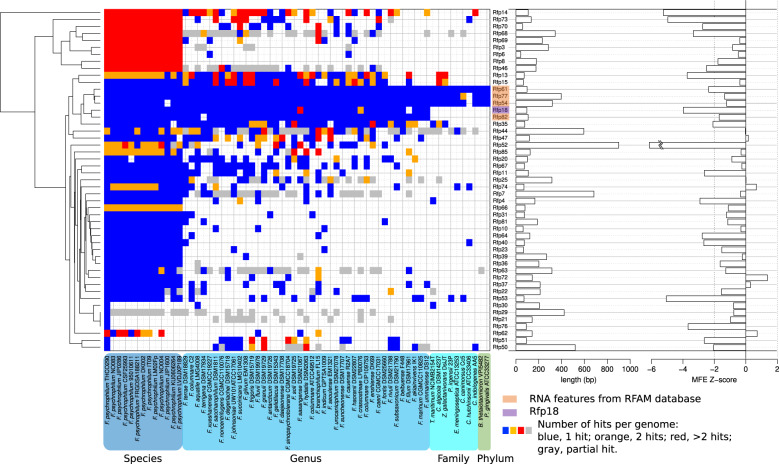
Conservation and secondary structure of Indep TRs. Heatmap representation of conservation profile for the 45 Indep TRs which are not antisense of CDSs and 4 RFAM RNAs. Length and evidence for secondary structure (low Minimum Free Energy z-score indicates significant folding) are represented as bar-plots on the right-hand side of the heatmap.

A total of 4 known RNAs (small signal recognition particle RNA, RNase P, transfer-messenger RNA and 6S RNA) and 85 newly described RNAs (indep TRs), named Rfp1-89, were detected (Table S8). Indep TRs were typically short (median length, 176 bp) and without coding potential; 40 were transcribed in antisense of CDSs, with a potential *cis*-regulatory effect (Table 3). Outside asRNAs whose conservation cannot be assessed independently of cognate CDSs, indep TR sequences tended to be conserved across the species *F. psychrophilum* and many may have homologs in other species of the genus (Fig. 3). Not surprisingly, known RNAs were among the most highly conserved. A fraction of these 45 non-antisense indep TRs probably act as *bona fide* regulatory sRNAs. sRNAs are key regulators that control numerous cellular processes by fine-tuning gene expression and usually act via base-pairing with several mRNAs, resulting in modulation of stability, structure and/or translation efficiency [65]. In the absence of dedicated molecular experimental studies, the discovery of their regulatory networks mainly relies on *in silico* sRNA-mRNA pairing predictions [66].

A strong secondary structure was predicted for 18 (40%) of the 45 Indep TRs not listed as antisense, which suggests functionality (SI Appendix D10). These included Rfp11, Rfp13, Rfp15, and Rfp18 which are conserved in genomes of other species (Fig. 3). Condition-dependent expression profiles revealed that most of the sRNAs were expressed under specific environmental conditions, a trend well known from studies in other bacteria [67].

Predictions of mRNA–sRNA interactions were examined to identify putative targets (Table S9). Despite well-known limitations (e.g., high number of false positives), this approach is efficient to select candidates for functional characterization, particularly when several targets with related functions are predicted for a same sRNA [68]. Among the 13 putative metalloprotease-encoding mRNAs identified in strain JIP02/86 [13], 4 were predicted as possible targets of Rfp18, a sRNA conserved outside the species *F. psychrophilum*, which likely folds into a strong secondary RNA structure (SI Appendix D11). Another predicted target encodes a putative secreted adhesin (THC0290_2338) [24]. The pairing region of Rfp18 was identical for all these predicted targets (third stem-loop). Regulation of proteases by sRNAs is already reported in other pathogenic bacteria, such as the collagenase ColA in *Clostridium perfringens*, the cysteine protease SpeB in *Streptococcus pyogenes* or the Vsm protease in *Vibrio* species [69–72]. *F. psychrophilum* produces several degradative enzymes (mainly metalloproteases) that allow bacterial cells to digest collagen, fibrinogen, elastin and fish muscle tissue, a trait proposed to participate to virulence and to promote tissue erosion in infected fish [13, 18, 19, 42]. Expression control of these putative virulence determinants remains to be elucidated and most have unknown substrates.

**Fig. 4:**
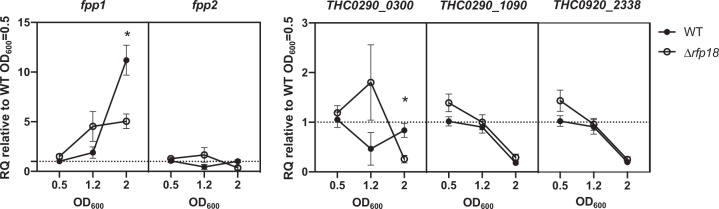
Rfp18 deletion affects the mRNA level of two metalloprotease encoding genes. RNAs from wild-type and Δ*rfp18* cultures sampled in exponential (OD600 = 0.5), transition (1.2) and stationary phase (2) were used for RT-qPCR assays. Relative quantification (2^−∆∆Ct^; RQ) of putative target mRNA is calculated using wild-type in exponential phase of growth as the reference condition. Values are the mean ± s.e.m from five biological replicates. (*) indicates significant difference using two-way ANOVA analysis (Bonferroni adjusted *p* value < 0.05).

A *rfp18* deletion mutant was constructed and the expression level of several predicted mRNA targets was compared in wild-type and Δ*rfp18* strains by RT-qPCR. The results showed that mRNA levels of two metalloproteases, Fpp1 and THC0290_0300, were significantly reduced in Δ*rfp18* during stationary phase (Fig. 4). Fpp1 is reported as transcribed together with the Fpp2 metalloprotease [26], which was confirmed here by identification of a single TSS. Regulation by Rfp18 did not affect Fpp2 mRNA level, and Rfp18 pairing, which was predicted in the 107-bp intergenic region of the *fpp2*-*fpp1* operon, appears thus to uncouple expression of the two genes. We confirmed expression of the homolog of Rfp18 in *Flavobacterium columnare*. Furthermore, predicted targets in this other serious fish pathogen are homologs to *F. psychrophilum* metalloproteases and adhesin THC0920_2338 (SI Appendix D11). Altogether, these results indicate that Rfp18 is required for the precise expression control of several metalloproteases and evolutionary conservation underlines the importance of this regulatory mechanism.

### Transcriptional changes in response to environmental transitions

**Fig. 5:**
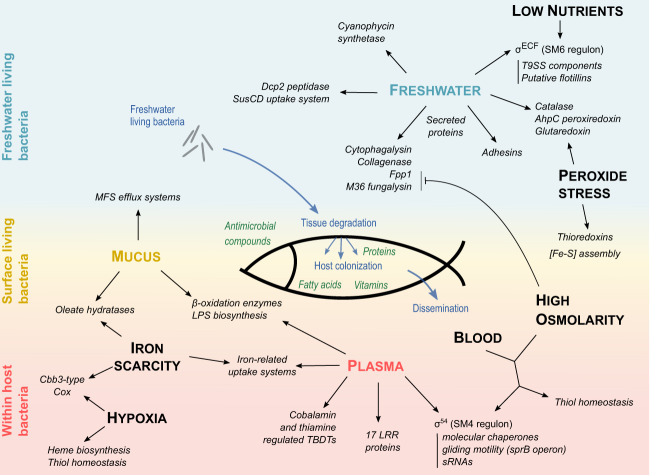
Graphical summary of the main functions identified to contribute to *F. psychrophilum* adaptation. In vitro conditions analyzed (uppercase letters) are positioned to reflect three environmental niches: freshwater living bacteria, external surface associated bacteria and within-host bacteria. The three conditions related to fish-derived components are in colored uppercase (freshwater-living bacteria, blue; fish mucus, yellow; fish plasma, red). Arrows indicate transcriptional up-regulation.

Pathogenic bacteria have to cope with diverse environments characterized by distinct constraints [73]. Several life stages are important for the success of *F. psychrophilum* as an aquatic pathogen: (i) life outside the host, ensuring long-term survival in water while keeping infectivity, (ii) attachment and life on the host surface, damaging tissues for successful invasion, and (iii) life inside the host, entering the bloodstream and colonizing organs until host’s death (Fig. 5). Environmental conditions greatly vary between outside and inside the fish from standpoints as diverse as osmotic pressure, exposure to host’s defenses, shifts in nutrient sources and concentrations. Osmotic pressure in body fluids is much higher than in freshwater or on fish surface. On the skin surface, the bacterium has to adapt in order to resist the host defense components present in the mucus barrier such as proteases, lysozyme, antimicrobial peptides, complement, lectins or immunoglobulins [74]. Specific in vitro conditions were designed to mimic outside, surface and inside host environmental niches, and others to establish more direct functional links between genes and specific stimuli (Table 1). DEGs were analyzed to predict functions and metabolic pathways involved in adaptation to these environmental conditions (Table S5, the *fpeb* website). As co-expressed genes (clusters) tend to have similar functions or to be part of the same biological pathway, we also formulate functional hypotheses for genes of unknown function based on correlation of expression profiles (Fig. 1). Results are reported in the following paragraphs and detailed in SI Appendix D12–D15.

#### Transcriptional adaptation of freshwater-living bacteria

Freshwater-living bacteria transcriptomes were established in fish rearing conditions, 24 h after inoculation of *F. psychrophilum* into tanks with or without rainbow trout (Table S1). Both transcriptome profiles differed markedly from TYES broth conditions, with patterns testifying of the nutritional deprivation of *F. psychrophilum* outside the host (SI Appendix D12). Dissolved O_2_ was maintained near to saturation into tank water (10.7 mg L^−1^, 10.5 °C) to meet respiration needs of rainbow trout and transcriptional responses related to redox homeostasis indicated bacterial adaptation to oxidative stress. Expression of iron acquisition systems suggested availability of ferrous iron in freshwater. Several peptidases genes were induced in both freshwater conditions. Fish skin contains large amounts of collagen and gelatin, whose hydrolysis-released peptides likely constitute an important source of nutrients for the bacterium during host invasion. Consistently, among the freshwater upregulated gene cluster B549, the collagenase, a SusCD family outer-membrane uptake system (THC0290_2100 and _2101) with homologs in the whole family *Flavobacteriaceae* and a peptidyl-dipeptidase Dcp2, could ensure the hydrolysis of collagen and the import of extracellular oligopeptides providing amino-acids for growth, as shown in *P. gingivalis* [75]. Their up-regulation was independent of the presence of fish, suggesting that freshwater-living bacteria are transcriptionally pre-adapted to host encountering. This adaptation could be driven by nutrient starvation as expression of genes belonging to cluster B549 also increased when cells enter into stationary phase.

#### Responses to fish components

In contrast to the collagenase, Fpp metalloproteases were induced in freshwater-living cells in the presence of fish only (SI Appendix D13). Co-expression of cyanophycin synthetase CphA with *fpp* and THC0290_0300 metalloproteases suggests that peptides released by these proteases may partly be used in *F. psychrophilum* for biosynthesis of cyanophycin, a branched non-ribosomal peptide composed of L-arginine and L-aspartate, commonly found in cyanobacteria and serving as a cytoplasmic reservoir for carbon, nitrogen and energy [13, 76]. Other genes specifically up-regulated in freshwater in the presence of fish included *ybcL* homolog, which encodes a protein inhibiting neutrophil migration in uropathogenic *E. coli* strains [77], suggesting that it may also counteract mucosal immune defenses in *F. psychrophilum*.

Genes related to fatty acids (FA) β-oxidation pathway were highly up-regulated in the presence of fish compounds such as mucus and plasma, suggesting that FA breakdown serves as an energy source. These observations indicate that FA could be scavenged from the host during the infectious process as observed in other bacterial pathogens [78]. This was quite unexpected as *F. psychrophilum* was believed to solely use proteinaceous compounds but consistent with reported lipolytic activity [18].

Transcriptional changes also attested to responses against harmful conditions. Genes highly upregulated under fish mucus exposure encoded oleate hydratases that confer bacterial resistance to antimicrobial FA in some pathogenic bacteria [79, 80], multidrug efflux pumps that are used to extrude host antimicrobial peptides and FA, and exopolysaccharides biosynthesis proteins. Similar global responses involving LPS modifications and efflux pumps are reported in Gram-negative pathogenic bacteria submitted to antimicrobial compounds [81]. The overlap observed between transcriptional responses under skin mucus and plasma exposure likely reflects the response to both mucosal and systemic innate immunity.

#### Life inside the host

Transcriptional analysis of stress responses, such as those induced by reactive oxygen species, hypoxia, or sequestration of essential metals, is an efficient way to discover genes required for virulence (SI Appendix D14) [73].

Peroxide stress response likely plays an important role in the resistance against bacterial killing during respiratory burst of phagocytes [73]. Hydrogen peroxide exposure led to a typical oxidative stress response in *F. psychrophilum*, characterized by the overexpression of antioxidative enzymes and components of [Fe-S] clusters assembly machinery. Uncharacterized transcription factors and conserved proteins of unknown function were also up-regulated suggesting their involvement in oxidative stress responses.

Inflammatory hypoxia can occur in infected tissues due to the activity of the numerous phagocytes recruited *in situ* [82]. As other pathogenic bacteria colonizing anoxic tissues [83], *F. psychrophilum* exposed to oxygen limitation adapt by a strong up-regulation of the high affinity *cbb3*-type cytochrome oxidase (*cbb3*-Cox) [83]. Also upregulated, enzymes of the heme biosynthesis pathway could provide the porphyrins required for assembly of newly synthetized *cbb3*-Cox complexes.

*F. psychrophilum* response to high osmotic pressure was characterized by the induction of osmoregulation system, gliding motility genes, the T9SS C-terminal signal peptidase PorU, as well as thiol-specific antioxidative enzymes. Growth on blood triggered transcriptional increase of those high osmolarity-induced genes, suggesting that osmotic pressure serves as a signal for activation of the oxidative stress response. Such coordinated regulation may anticipate the respiratory burst when the bacterium enters body fluids.

Bacterial pathogens have evolved to perceive iron scarcity as a marker of the host’s internal environment and have developed mechanisms to evade this nutritional immunity [84]. *F. psychrophilum* response under metal deprivation was characterized by the up-regulation of several TBDTs, which could be involved in iron acquisition, and other uncharacterized genes expressed in iron-deficient conditions in other bacteria. FA detoxification and hypoxia-induced genes were also part of the response to iron scarcity. Co-induction of these genes by several stimuli reveals a common response to the multiple stresses faced during host colonization.

Iron scarcity, hypoxia, osmotic and peroxide stress responses described above were all part of the global transcriptional adaptation of *F. psychrophilum* cells exposed to fish plasma. Other genes induced by plasma exposure encoded efflux pumps, TBDTs that may play a role in blood-derived nutrients acquisition, uncharacterized transcription factors, and several enzymes involved in O-antigen biosynthesis whose modulation may participate to the resistance to killing by the host’s complement.

A comparative proteomic analysis of *F. psychrophilum* identified 20 proteins modulated in vivo in rainbow trout [85]. Half of them corresponded to DEGs under our in vitro conditions (SI Appendix D14), which validates the strategy of mimicking within-host environments to provide a functional context to unknown genes at the genome scale.

#### Expression of putative virulence factors across environmental conditions

The T9SS is known as essential for *F. psychrophilum* virulence in rainbow trout, however most proteins predicted as secreted are uncharacterized and those contributing to virulence are not identified [24, 27]. Several T9SS secreted protein encoding genes were up-regulated under within-host mimicking conditions while other were up-regulated in freshwater and might have a role in host invasion (SI Appendix D15). Among the putative secreted adhesins, 17 tandem-arranged Leucine-rich repeat (LRR) proteins were induced under fish plasma exposure. In other species, LRR proteins can mediate host–pathogen interactions allowing adhesion to surface receptors of host immune cells [86]. Many secreted proteins are predicted peptidases and some are suspected to play a role in adaptation to specific fish hosts [13, 87]. Here, half of secreted proteases were modulated by many biological conditions, and some appeared more dedicated to outside-host or within-host conditions. Dedicated experiments confirmed variations of exoproteolytic activity consistent with the transcriptional changes. In particular, activity was reduced under high osmotic pressure or presence of serum, two conditions mimicking life inside host (SI Appendix D15). Altogether, these results document how *F. psychrophilum* adapts its pool of secreted degradative enzymes and allow to formulate hypotheses on their respective importance at particular life stages. Expression of this large variety of degradative enzymes and their refined regulation at environmental transitions highlight sophisticated adaptations to a pathogenic lifestyle.

## Conclusion

Due to their diverse ecological niches and their important contribution to aquatic ecosystems, there is a great interest in flavobacteria [2]. Their biology reveals original features shared with other members of the phylum *Bacteroidetes* such as their gene expression signals, protein secretion machinery, mode of locomotion by gliding or unique outer membrane systems dedicated to nutrients acquisition [9, 56, 75, 88, 89]. We describe here the first RNA landscape of a flavobacteria and the molecular changes taking place when a pathogen of this family adapts to the diverse environments met during its life-cycle, by using an array of specifically designed in vitro conditions. The results highlight similarities with other, better known, bacteria and original characteristics linked to the position in the phylum *Bacteroidetes* and the ecological niche of an aquatic pathogen. By pointing proteins and regulatory elements probably involved in host-pathogen interactions, metabolic pathways, and molecular machineries, the results suggest many directions for future research; a website is made available to facilitate their use to fill knowledge gaps on flavobacteria.

## Supplementary Information

Supplementary Information

Table S1

Table S2

Table S3

Table S4

Table S5

Table S6

Table S7

Table S8

Table S9

## Data Availability

Expression datasets are deposited in GEO (accession numbers GSE163842, GSE164189 and GSE164190). The *F. psychrophilum* codon-optimized mCherry sequence is deposited in GenBank (accession number MW401799).

## References

[1] FAO, editor The state of world fisheries and aquaculture. Rome: Food and Agriculture Organization (FAO); 2018.

[2] Fernandez-Gomez B (2013). Ecology of marine *Bacteroidetes*: a comparative genomics approach. ISME J.

[3] Bernardet J. F. *Flavobacteriaceae* Bergey’s manual of systematics of archaea and bacteria; 2015.

[4] Nematollahi A, Decostere A, Pasmans F, Haesebrouck F (2003). *Flavobacterium psychrophilum* infections in salmonid fish. J Fish Dis.

[5] Barnes ME, Brown ML (2011). A review of *Flavobacterium psychrophilum* biology, clinical signs, and Bacterial Cold Water Disease prevention and treatment. Open Fish Sci J.

[6] Bayliss SC (2017). The promise of whole genome pathogen sequencing for the molecular epidemiology of emerging aquaculture pathogens. Front Microbiol.

[7] Evensen O, Lorenzen E (1996). An immunohistochemical study of *Flexibacter psychrophilus* infection in experimentally and naturally infected rainbow trout (*Oncorhynchus mykiss*) fry. Dis Aquatic Org.

[8] Bayley DP, Rocha ER, Smith CJ (2000). Analysis of *cepA* and other *Bacteroides fragilis* genes reveals a unique promoter structure. FEMS Microbiol Lett.

[9] Vingadassalom D (2005). An unusual primary sigma factor in the *Bacteroidetes* phylum. Mol Microbiol.

[10] Chen S, Bagdasarian M, Kaufman MG, Walker ED (2007). Characterization of strong promoters from an environmental *Flavobacterium hibernum* strain by using a green fluorescent protein-based reporter system. Appl Environ Microbiol.

[11] Baez WD (2019). Global analysis of protein synthesis in *Flavobacterium johnsoniae* reveals the use of Kozak-like sequences in diverse bacteria. Nucleic Acids Res.

[12] Wegmann U, Horn N, Carding SR (2013). Defining the bacteroides ribosomal binding site. Appl Environ Microbiol.

[13] Duchaud E (2007). Complete genome sequence of the fish pathogen *Flavobacterium psychrophilum*. Nature Biotechnol.

[14] Wiens GD, et al. Complete genome sequence of *Flavobacterium psychrophilum* strain CSF259-93, used to select rainbow trout for increased genetic resistance against Bacterial Cold Water Disease. Genome Announc. 2014;2:e00889-14. 10.1128/genomeA.00889-14 PMC4172266 25237017

[15] Castillo D, Christiansen RH, Dalsgaard I, Madsen L, Middelboe M (2015). Bacteriophage resistance mechanisms in the fish pathogen *Flavobacterium psychrophilum*: linking genomic mutations to changes in bacterial virulence factors. Appl Environ Microbiol.

[16] Rochat T, et al. Complete genome sequence of *Flavobacterium psychrophilum* strain OSU THCO2-90, used for functional genetic analysis. Genome Announc. 2017;5:e01665-16. 10.1128/genomeA.01665-16 PMC5323625 28232446

[17] Duchaud E (2018). Genomic diversity and evolution of the fish pathogen *Flavobacterium psychrophilum*. Front Microbiol.

[18] Bernardet JF, Kerouault B (1989). Phenotypic and genomic studies of “*Cytophaga psychrophila*” isolated from diseased rainbow trout (*Oncorhynchus mykiss*) in France. Appl Environ Microbiol.

[19] Otis EJ. Lesions of cold-water disease in steelhead trout (*Salmo gairdneri*): the role of *Cytophaga psychrophila* extracellular products. [MSc thesis]: University of Rhode Island, Kingston; 1984.

[20] Hogfors-Ronnholm E, Wiklund T (2010). Hemolytic activity in *Flavobacterium psychrophilum* is a contact-dependent, two-step mechanism and differently expressed in smooth and rough phenotypes. Microbial Pathog.

[21] Hogfors-Ronnholm E, Wiklund T (2012). In vitro opsonin-independent interactions between cells of smooth and rough phenotypes of *Flavobacterium psychrophilum* and rainbow trout (*Oncorhynchus mykiss*) head kidney macrophages. Microbial Pathog.

[22] Papadopoulou A, Dalsgaard I, Linden A, Wiklund T (2017). In vivo adherence of *Flavobacterium psychrophilum* to mucosal external surfaces of rainbow trout (*Oncorhynchus mykiss*) fry. J Fish Dis.

[23] Decostere A, D’Haese E, Lammens M, Nelis H, Haesebrouck F (2001). In vivo study of phagocytosis, intracellular survival and multiplication of *Flavobacterium psychrophilum* in rainbow trout, *Oncorhynchus mykiss* (Walbaum), spleen phagocytes. J Fish Dis.

[24] Barbier P, et al. The type IX secretion system is required for virulence of the fish pathogen *Flavobacterium psychrophilum*. Appl Environ Microbiol. 2020;86:e00799-20. 10.1128/AEM.00799-20 PMC7414955 32532872

[25] Alvarez B, Alvarez J, Menendez A, Guijarro JA (2008). A mutant in one of two *exbD* loci of a TonB system in *Flavobacterium psychrophilum* shows attenuated virulence and confers protection against cold water disease. Microbiology.

[26] Pérez-Pascual D (2011). Comparative analysis and mutation effects of *fpp2-fpp1* tandem genes encoding proteolytic extracellular enzymes of *Flavobacterium psychrophilum*. Microbiology.

[27] Pérez-Pascual D (2017). More than gliding: Involvement of GldD and GldG in the virulence of *Flavobacterium psychrophilum*. Front Microbiol.

[28] Madetoja J, Nystedt S, Wiklund T (2003). Survival and virulence of *Flavobacterium psychrophilum* in water microcosms. FEMS Microbiol Ecol.

[29] Vatsos IN, Thompson KD, Adams A (2003). Starvation of *Flavobacterium psychrophilum* in broth, stream water and distilled water. Dis Aquat Organ.

[30] Sharma CM (2010). The primary transcriptome of the major human pathogen *Helicobacter pylori*. Nature..

[31] Toledo-Arana A (2009). The *Listeria* transcriptional landscape from saprophytism to virulence. Nature..

[32] Innocenti N (2015). Whole-genome mapping of 5′ RNA ends in bacteria by tagged sequencing: a comprehensive view in *Enterococcus faecalis*. RNA..

[33] Kroger C (2018). The primary transcriptome, small RNAs and regulation of antimicrobial resistance in *Acinetobacter baumannii* ATCC 17978. Nucleic Acids Res.

[34] Heidrich N, Bauriedl S, Barquist L, Li L, Schoen C, Vogel J (2017). The primary transcriptome of *Neisseria meningitidis* and its interaction with the RNA chaperone Hfq. Nucleic Acids Res.

[35] Kroger C (2013). An infection-relevant transcriptomic compendium for *Salmonella enterica* Serovar Typhimurium. Cell Host Microbe.

[36] Mader U (2016). *Staphylococcus aureus* transcriptome architecture: from laboratory to infection-mimicking conditions. PLoS Genet.

[37] Nicolas P (2012). Condition-dependent transcriptome reveals high-level regulatory architecture in *Bacillus subtilis*. Science..

[38] Ficko-Blean E (2017). Carrageenan catabolism is encoded by a complex regulon in marine heterotrophic bacteria. Nat Commun.

[39] Gomez-Consarnau L (2016). Proteorhodopsin light-enhanced growth linked to vitamin-B1 acquisition in marine Flavobacteria. ISME J.

[40] Tang K, Lin Y, Han Y, Jiao N (2017). Characterization of potential polysaccharide utilization systems in the marine *Bacteroidetes Gramella Flava* JLT2011 using a multi-omics approach. Front Microbiol..

[41] Tekedar HC (2017). Comparative genomics and transcriptional analysis of *Flavobacterium columnare* strain ATCC 49512. Front Microbiol.

[42] Bertolini JM, Wakabayashi H, Watral VG, Whipple MJ, Rohovec JS (1994). Electrophoretic detection of proteases from selected strains of *Flexibacter psychrophilus* and assesment of their variability. J Aquat Anim Health.

[43] Zhu Y (2017). Genetic analyses unravel the crucial role of a horizontally acquired alginate lyase for brown algal biomass degradation by *Zobellia galactanivorans*. Environ Microbiol.

[44] Rochat T, et al. Identification of a novel elastin-degrading enzyme from the fish pathogen *Flavobacterium psychrophilum*. Appl Environ Microbiol. 2019;85:e02535-18. 10.1128/AEM.02535-18 PMC6414381 30635380

[45] Kalvari I (2018). Rfam 13.0: shifting to a genome-centric resource for non-coding RNA families. Nucleic Acids Res.

[46] Mirauta B, Nicolas P, Richard H (2014). Parseq: reconstruction of microbial transcription landscape from RNA-Seq read counts using state-space models. Bioinformatics..

[47] Buels R (2016). JBrowse: a dynamic web platform for genome visualization and analysis. Genome Biol.

[48] Priyam A (2019). Sequenceserver: a modern graphical user interface for custom BLAST databases. Mol Biol Evol.

[49] Irnov I, Sharma CM, Vogel J, Winkler WC (2010). Identification of regulatory RNAs in *Bacillus subtilis*. Nucleic acids Res.

[50] Kim D (2012). Comparative analysis of regulatory elements between *Escherichia coli* and *Klebsiella pneumoniae* by genome-wide transcription start site profiling. PLoS Genet.

[51] Ryan D, Jenniches L, Reichardt S, Barquist L, Westermann AJ (2020). A high-resolution transcriptome map identifies small RNA regulation of metabolism in the gut microbe *Bacteroides thetaiotaomicron*. Nature Commun.

[52] Barrios H, Valderrama B, Morett E (1999). Compilation and analysis of sigma(54)-dependent promoter sequences. Nucleic Acids Res.

[53] Francke C (2011). Comparative analyses imply that the enigmatic Sigma factor 54 is a central controller of the bacterial exterior. BMC Genom.

[54] Staron A (2009). The third pillar of bacterial signal transduction: classification of the extracytoplasmic function (ECF) sigma factor protein family. Mol Microbiol.

[55] Kadowaki T (2016). A two-component system regulates gene expression of the type IX secretion component proteins via an ECF sigma factor. Sci Rep.

[56] Sato K (2010). A protein secretion system linked to bacteroidete gliding motility and pathogenesis. Proc Natl Acad Sci USA.

[57] Lopez D, Koch G (2017). Exploring functional membrane microdomains in bacteria: an overview. Curr Opin Microbiol.

[58] Helmann JD (2002). The extracytoplasmic function (ECF) sigma factors. Adv Microb Physiol.

[59] Kulkarni SS, Johnston JJ, Zhu Y, Hying ZT, McBride MJ. The carboxy-terminal region of *Flavobacterium johnsoniae* SprB facilitates its secretion by the Type IX secretion system and propulsion by the gliding motility machinery. J Bacteriol. 2019;201:e00218-19. 10.1128/JB.00218-19 PMC6755757 31262839

[60] Rhodes RG, Nelson SS, Pochiraju S, McBride MJ (2011). *Flavobacterium johnsoniae sprB* is part of an operon spanning the additional gliding motility genes *sprC*, *sprD*, and *sprF*. J Bacteriol.

[61] Georg J, Hess WR. Widespread antisense transcription in prokaryotes. Microbiol Spectr. 2018;6. 10.1128/microbiolspec.rwr-0029-2018 PMC11633618 30003872

[62] Cao Y, Forstner KU, Vogel J, Smith CJ (2016). *cis*-encoded small RNAs, a conserved mechanism for repression of polysaccharide utilization in *Bacteroides*. J Bacteriol.

[63] Gollnick P, Babitzke P (2002). Transcription attenuation. Biochim Biophys Acta.

[64] Weinberg Z (2010). Comparative genomics reveals 104 candidate structured RNAs from bacteria, archaea, and their metagenomes. Genome Biol.

[65] Jorgensen MG, Pettersen JS, Kallipolitis BH (2020). sRNA-mediated control in bacteria: an increasing diversity of regulatory mechanisms. Biochim Biophys Acta Gene Regul Mech.

[66] Georg J (2020). The power of cooperation: experimental and computational approaches in the functional characterization of bacterial sRNAs. Mol Microbiol.

[67] Holmqvist E, Wagner EGH (2017). Impact of bacterial sRNAs in stress responses. Biochem Soc Trans.

[68] Pain A (2015). An assessment of bacterial small RNA target prediction programs. RNA Biol.

[69] Kovacikova G, Skorupski K (2002). Regulation of virulence gene expression in *Vibrio cholerae* by quorum sensing: HapR functions at the *aphA* promoter. Mol Microbiol.

[70] Mangold M (2004). Synthesis of group A streptococcal virulence factors is controlled by a regulatory RNA molecule. Mol Microbiol.

[71] Nguyen AN, et al. *csrB* gene duplication drives the evolution of redundant regulatory pathways controlling expression of the major toxic secreted metalloproteases in *Vibrio tasmaniensis* LGP32. mSphere. 2018;3:e00582-18. 10.1128/mSphere.00582-18 PMC6262261 30487156

[72] Obana N, Shirahama Y, Abe K, Nakamura K (2010). Stabilization of *Clostridium perfringens* collagenase mRNA by VR-RNA-dependent cleavage in 5′ leader sequence. Mol Microbiol.

[73] Fang FC, Frawley ER, Tapscott T, Vazquez-Torres A (2016). Bacterial stress responses during host infection. Cell Host Microbe.

[74] Ángeles Esteban M. An overview of the immunological defenses in fish skin. International Scholarly Research Notices, 2012;2012:853470.

[75] Madej M, et al. Structural and functional insights into oligopeptide acquisition by the RagAB transporter from *Porphyromonas gingivalis*. Nat Microbiol. 2020;5:1016–25. 10.1038/s41564-020-0716-y PMC7610489 32393857

[76] Ziegler K (1998). Molecular characterization of cyanophycin synthetase, the enzyme catalyzing the biosynthesis of the cyanobacterial reserve material multi-L-arginyl-poly-L-aspartate (cyanophycin). Eur J Biochem.

[77] Lau ME, Loughman JA, Hunstad DA (2012). YbcL of uropathogenic *Escherichia coli* suppresses transepithelial neutrophil migration. Infect Immun.

[78] Fozo EM, Rucks EA (2016). The making and taking of lipids: the role of bacterial lipid synthesis and the harnessing of host lipids in bacterial pathogenesis. Adv Microb Physiol.

[79] Subramanian C, Frank MW, Batte JL, Whaley SG, Rock CO (2019). Oleate hydratase from *Staphylococcus aureus* protects against palmitoleic acid, the major antimicrobial fatty acid produced by mammalian skin. J Biol Chem.

[80] Volkov A (2010). Myosin cross-reactive antigen of *Streptococcus pyogenes* M49 encodes a fatty acid double bond hydratase that plays a role in oleic acid detoxification and bacterial virulence. J Biol chem.

[81] Band VI, Weiss DS (2015). Mechanisms of antimicrobial peptide resistance in Gram-negative bacteria. Antibiotics..

[82] Campbell EL (2014). Transmigrating neutrophils shape the mucosal microenvironment through localized oxygen depletion to influence resolution of inflammation. Immunity..

[83] Pitcher RS, Watmough NJ (2004). The bacterial cytochrome *cbb3* oxidases. Biochim Biophys Acta.

[84] Skaar EP (2010). The battle for iron between bacterial pathogens and their vertebrate hosts. PLoS Pathog.

[85] LaFrentz BR, LaPatra SE, Call DR, Wiens GD, Cain KD (2009). Proteomic analysis of *Flavobacterium psychrophilum* cultured and in iron-limited media. Dis Aquat Org.

[86] Loimaranta V (2009). Leucine-rich repeats of bacterial surface proteins serve as common pattern recognition motifs of human scavenger receptor gp340. J Biol Chem.

[87] Nakayama H, Tanaka K, Teramura N, Hattori S (2016). Expression of collagenase in *Flavobacterium psychrophilum* isolated from cold-water disease-affected ayu (*Plecoglossus altivelis*). Biosci Biotechnol Biochem.

[88] Martens EC, Koropatkin NM, Smith TJ, Gordon JI (2009). Complex glycan catabolism by the human gut microbiota: the *Bacteroidetes* Sus-like paradigm. J Biol Chem.

[89] McBride MJ. *Bacteroidetes* gliding motility and the Type IX secretion system. Microbiol Spectr. 2019;7. 10.1128/microbiolspec.psib-0002-2018 PMC11588200 30767845

