# Supplementary material for: Transcriptome architecture and regulation at environmental transitions in flavobacteria: the case of an important fish pathogen
Source: ISME Commun. 2021 Jul 7;1:33. doi: 10.1038/s43705-021-00029-9 (PMC9723704; doi:10.1038/s43705-021-00029-9)
Supplement: Supplementary file 1 — Supplementary Information [file 43705_2021_29_MOESM1_ESM.docx]

**SUPPLEMENTARY INFORMATION**

[SI Appendix, Supplementary data 3](#_Toc70541120)

[D1. Identification and classification of transcribed regions outside annotated CDSs 3](#_Toc70541121)

[D2. Gene co-expression clusters and Principal Component Analysis. 5](#_Toc70541122)

[D3. Classification of 5’-ends 7](#_Toc70541123)

[D4. Condition-dependent expression profiles of the primary and alternative sigma factors. 7](#_Toc70541124)

[D5. Promoter sequence discovery and classification into sigma factor regulons. 10](#_Toc70541125)

[D6. Two promoters control the expression of the gliding motility operon *remFG-sprCDBF* 11](#_Toc70541126)

[D7. Antisense transcription mainly originates from 3’ extended regions and non-coding RNAs. 11](#_Toc70541127)

[D8. 5’ TRs belonging to known *cis-*regulatory RNA families*.* 14](#_Toc70541128)

[D9. New 5’ *cis*-regulatory RNAs candidates 14](#_Toc70541129)

[D10. Small regulatory RNAs 18](#_Toc70541130)

[D11. Rfp18, a putative regulatory sRNA predicted to control several metalloproteases. 20](#_Toc70541131)

[D12. Transcriptional adaptation of freshwater-living bacteria 24](#_Toc70541132)

[D13. Responses to fish compounds 26](#_Toc70541133)

[D14. Life inside the host 28](#_Toc70541134)

[D15. Expression of putative virulence factors across environmental conditions 34](#_Toc70541135)

[SI Appendix, Materials and Methods 38](#_Toc70541136)

[M1. Bacterial strains and growth conditions 38](#_Toc70541137)

[M2. Construction of transcriptional reporter plasmids and promoter activity monitoring 38](#_Toc70541138)

[M3. Construction of the *rfp18* deletion mutant 40](#_Toc70541139)

[M4. RNA extraction, libraries preparation and RNA-sequencing 40](#_Toc70541140)

[M5. Read mapping 41](#_Toc70541141)

[M6. Determination of putative TSSs 41](#_Toc70541142)

[M7. Delineation of newly defined transcribed regions 41](#_Toc70541143)

[M8. High-density microarray design and strand-specific hybridization procedure 42](#_Toc70541144)

[M9. Sigma factors binding sites discovery 43](#_Toc70541145)

[M10. Computational analysis of newly defined transcribed regions 43](#_Toc70541146)

[M11. Real Time quantitative PCR 43](#_Toc70541147)

[M12. Reverse Transcription PCR 44](#_Toc70541148)

[SI Appendix, Supplementary Tables 45](#_Toc70541149)

[T1. Strains and plasmids. 45](#_Toc70541150)

[T2. Oligonucleotides. 46](#_Toc70541151)

[T3. Description of the set of biological conditions analyzed by microarrays. 47](#_Toc70541152)

[T4: Number of differentially expressed features across 23 pairwise comparisons of biological conditions. 48](#_Toc70541153)

[Supplementary references 49](#_Toc70541154)

SI Appendix, Supplementary data

# D1. Identification and classification of transcribed regions outside annotated CDSs

The ~17 million reads generated by sequencing the global transcriptome RNA pool library allowed to identify 1511 TRs outside annotated CDSs or RFAM features. These TRs were classified according to their transcriptional context. A full description of all these TRs is available in **Table S4** and on the ***fpeb*** website.

**
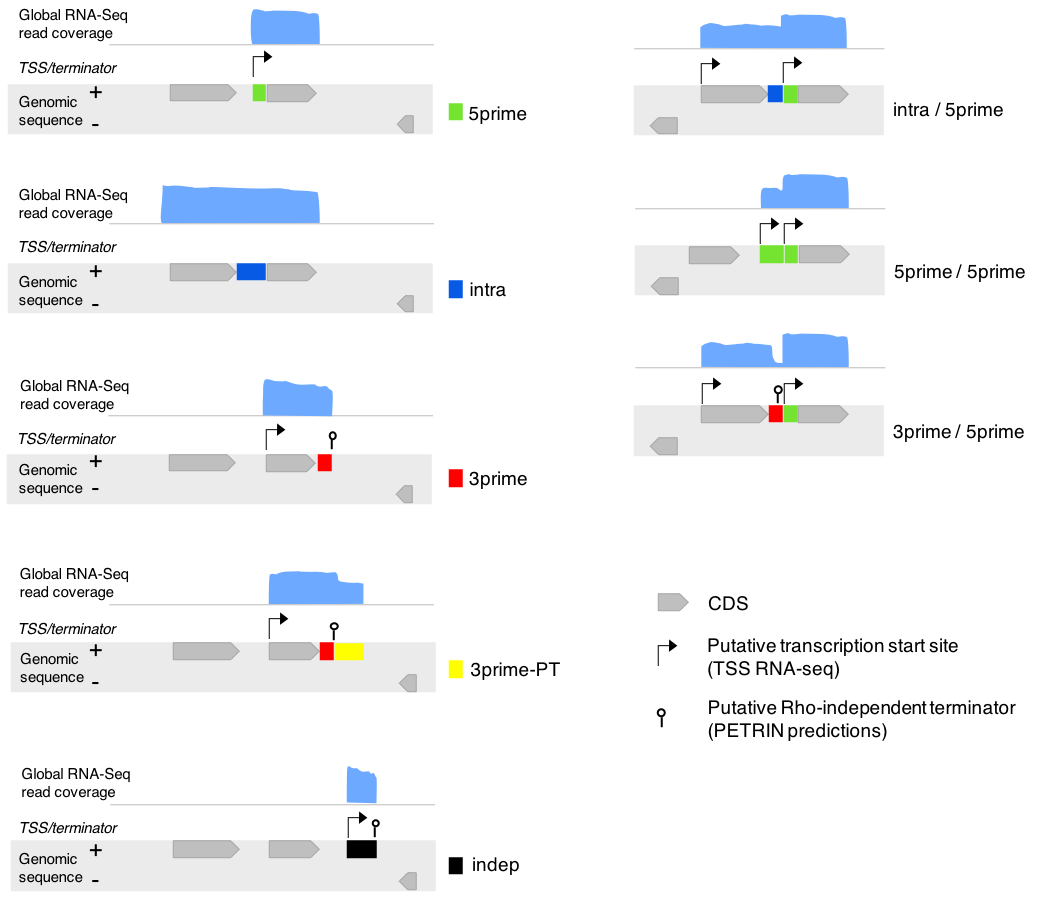
**

**Classification scheme of the 1511 TRs into RNA categories.**

[5prime] = 5’ transcribed region; [intra] = transcribed region lying between two genes, part of a polycistronic mRNA; [3prime] = 3’ untranslated region; [3prime-PT] = 3’ transcribed region resulting from partial termination of transcription (segment located downstream a 3prime region and a Rho-independent terminator); [indep] = region transcribed independently of previously annotated features

The validity of expression was confirmed for at least 87% of the 1511 TRs based on their expression level in the *in vitro* condition-dependent transcriptome dataset.

**Proportion of TRs expressed, in at least one sample, at low, middle and high level across *in vitro* conditions, relative to CDSs expression level.**

| Category of TRs | Total number (#) | TRs of length < 150bp | | | | | TRs of length ≥ 150 bp | | | | |
| --- | --- | --- | --- | --- | --- | --- | --- | --- | --- | --- | --- |
|  |  | # | withexp^a^ | Q25^b^ | Q50^c^ | Q75^d^ | # | withexp^a^ | Q25^b^ | Q50^c^ | Q75^d^ |
| 5’ | 231 | 182 | 150 | 140 | 126 | 96 | 49 | 49 | 49 | 44 | 30 |
| 3’ | 586 | 333 | 204 | 139 | 95 | 65 | 253 | 253 | 220 | 129 | 48 |
| 3’ PT | 67 | 13 | 10 | 8 | 5 | 5 | 54 | 54 | 42 | 18 | 7 |
| Intra | 542 | 374 | 327 | 291 | 244 | 156 | 168 | 168 | 160 | 132 | 79 |
| Indep | 85 | 40 | 37 | 36 | 33 | 20 | 45 | 45 | 41 | 30 | 20 |

^a^ number of new TRs for which expression level is available in the *in vitro* condition data-set (specific probes available)

^b^ number of new TRs with expression level that exceeds, in at least one sample, the expression value of the 25% less expressed CDSs

^c^ the median expression value; ^d^ the expression value of the 75% most highly expressed CDSs.

A first group of 231 TRs (category 5’) correspond to regions extending upstream of the start codon of annotated CDSs, where translation initiation elements are usually located. Most of these 5’ UTRs are of small size (median, 80 bp). However, some are long (49 TRs of length ≥ 150 bp) and encompass *cis*-acting regulatory elements such as riboswitches or transcription attenuation RNA elements.

Among regions extending in 3’, a small group (category 3’-PT) accounts for 67 TRs that extend (median, 314 bp) downstream predicted intrinsic terminators and thus apparently result from imperfect termination of transcription. Interestingly, the group of 586 other TRs classified as 3’ is also composed of a mixture between short TRs that tend to terminate at a defined position and long TRs that tend not to terminate at defined termination sites and characterized by gradual decrease of expression. Lack of correspondence between 3’-ends of regions extended in 3’ and intrinsic terminators indicated imprecise termination for a majority of long (>150 bp) 3’ regions: the 3’-ends of 87.7 % of the 333 short 3’ TRs coincide with predicted intrinsic terminators but this proportion drops to 19.4% for the 253 long 3’ TRs and is as low as 9.0% for the 67 TRs in the 3’-PT category.

Another group of 542 TRs, named ‘intra’, was composed of regions between consecutive CDSs on the same strand, most of them being within polycistronic mRNAs. Several carry a predicted open reading frame, such as THC0290_N_0396 or THC0290_N_0391 which could encode small uncharacterized proteins missing from the current genome annotation. Some are very long untranslated regions (up to 996 bp for THC0290_N_1363) and their role remain unknown.

Lastly, the ‘indep’ category encompasses 85 RNAs transcribed independently of any CDS annotation, among which are expected *bona-fide* regulatory RNAs (also named sRNAs). Besides, 4 ‘indep’ TRs carry small putative CDSs among which two are transcribed in antisense of each other (THC0290_N_0389 and THC0290_N_0390) and another one (THC0290_N_0643) encodes a putative exported protein and is part of gene cluster A1751 containing genes functionally related to redox homeostasis.

# D2. Gene co-expression clusters and Principal Component Analysis.

Gene co-expression clusters were defined based on pairwise Pearson distance (1 – r) of expression profiles. Three statistically significant correlation levels (r=0.8, 0.6, and 0.4) were used to define A-, B- and C-clusters. This use of an average-link hierarchical clustering tree based on Pearson distance to define co-expression clusters (1, 2) ensures consistency across the three levels of clustering (A-clusters subdivide B-clusters which subdivide C-clusters) and does not induce artificial grouping of genes in co-expression clusters (a drawback of many approaches discussed in Abu-Jamous and Kelly 2018 (3)): 32% of the genes are in the 69% of A-clusters that are of size 1.

**Significance of the three levels of co-expression clusters**

A. Distribution of pairwise Pearson correlation coefficients between the 3981 genes and transcribed regions (~7.9e+6 pairs). This distribution is compared to the distribution under the null hypothesis of uncorrelated expression profiles obtained by 10 random shuffling the sample labels for each gene (dashed line) and to the three reference values (r=0.8, 0.6, and 0.4) at which the hierarchical clustering tree was cut to define co-expression clusters.

B. Cumulated number of genes in clusters when including clusters in order of decreasing size for A-, B- and C-clusters. The subplots corresponding to clusters of size >1 are delineated by dotted lines (583/1876 A-clusters, 381/578 B-clusters, 121/148 C-clusters). The insert plot displays illustrative examples of relationships at the three reference Pearson correlation levels. For each level, it also reports the corresponding p-value computed, either with the standard one-tailed test implemented in R “cor.test” function (i.e. based on asymptotic confidence intervals based on Fisher's Z transform), or as the quantile of the shuffled distribution shown in panel A (italicized).

Principal Component Analysis of the transcriptomes revealed highly coordinated changes in gene expression: the position of the 64 samples on the first axis already explains as much as 48.5% of the total variance of the dataset, reflecting changes across growth stages, and the next three axes highlight the main transcriptome variations associated with fish plasma exposure (PC2), growth on blood (PC3) and survival in water (PC4).

**PCA axes 1 to 10: rmse's and coordinates of the biological conditions.**

Upper panel: Coordinates of samples on the PCA axes 1-5 and axes 6-10 are plotted separately. Coordinates are rescaled (and shifted) for each axis such as to span exactly the (0,1) interval.

Lower panel: root mean square error (rmse) between the gene expression levels as predicted by the coordinates of the sample in the PCA projection and measured gene expression levels as previously described (1). Rmse decreases gradually (predictions become more accurate) when the dimension of the PCA projection increases. PCA axes 1-10 are represented by staked colored surfaces whose widths indicate the decrease in rmse associated to each axis. To make the identification of the axis corresponding to each significant surface area easier we recycled 5 different colors 2 times to represent the 10 axes (separating by black lines each 2 group of axes).

The percentage of total variance explained by each axis is indicated between parentheses.

# D3. Classification of 5’-ends

Deep sequencing of the 5’-end RNA-Seq library resulted in ~7.5 million reads which were analyzed to retrieve putative TSSs as peaks above background signal. Altogether, 1884 genomic positions were identified as enriched in 5’-ends (**Table S3**).


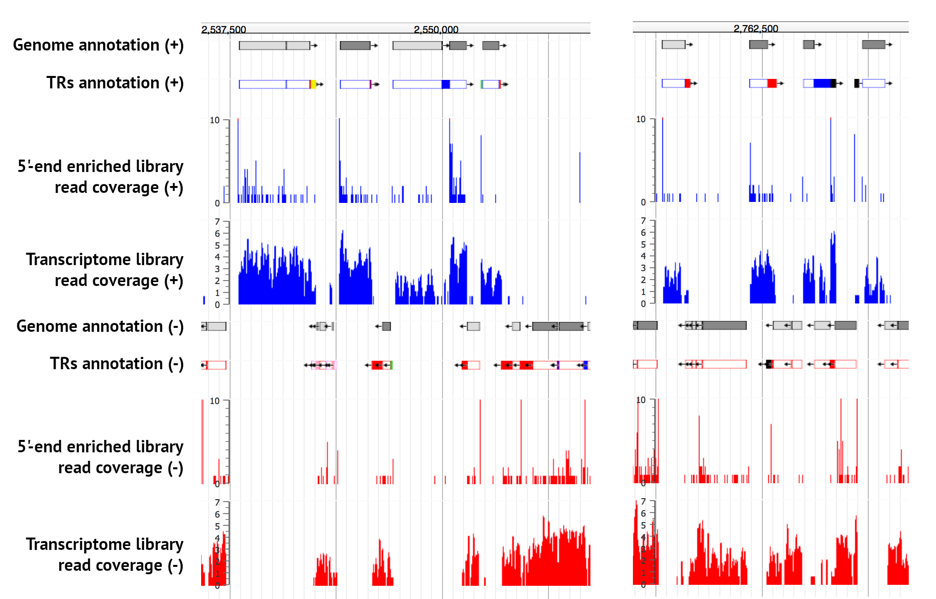


**Two genomic views of reads coverage profiles of the 5’-ends enriched and global RNA-Seq libraries of *F. psychrophilum*.**

These putative TSSs were classified into 5 groups according to their genomic context with respect to annotated CDSs. Those in the main group (64.4%) are less than 500 bp upstream of start codons of annotated CDSs, which can be considered as the canonical position for a TSS. The other contexts are inside a CDS on the sense strand (26.8%) or on the antisense strand (4.4%), and intergenic (between CDSs) but not upstream a start codon (4.4%). The last group (55 positions) correspond to the 5’-ends of conserved RNAs already reported in the RFAM database, including tRNAs, small signal recognition particle RNA, tmRNA, RNase P RNA, *etc*.

**Repartition of putative TSSs into different categories according to the genomic context of 5’-ends.**

|  | all | canonical | sense | antisense | inter | RNA |
| --- | --- | --- | --- | --- | --- | --- |
| All 5’-ends | 1884 | 1178 | 491 | 80 | 80 | 55 |
| 5’-ends (excluding redundant sequences) | 1868 | 1170 | 491 | 80 | 72 | 55 |
| 5’-ends with predicted sigma factor motif | 1194 | 951 | 73 | 76 | 57 | 37 |

Sigma factor binding sites were predicted for 1194 (63.4%) of the initial list of putative TSSs (**Table S2**) with important differences between genomic contexts. The proportion of TSSs confirmed by the presence of a sigma factor binding site is of 81.2% for putative TSSs in canonical and intergenic contexts, 95.0% for putative TSSs in antisense, but 14.9% for putative TSSs in sense. These proportions suggest a good sensitivity of *in silico* sigma factor binding site detection and good specificity of experimental mapping of TSSs, except for a majority of the putative TSSs in sense which probably correspond to 5’-ends of mRNA degradation products.

# D4. Condition-dependent expression profiles of the primary and alternative sigma factors.

A total of 9 sigma factor encoding genes were identified in the genome of *F. psychrophilum* OSU THCO2-90 using prediction of pfam domain PF04542 which corresponds the region structurally conserved among the most divergent family members of σ^70^, and of pfam domain PF04552 which corresponds to the highly conserved RpoN box of the σ^54^ family. Therefore, alongside the primary sigma factor σ^A^ encoded by *rpoD,* *F. psychrophilum* contains 8 alternative sigma factors: 1 σ^54^ factor encoded by *rpoN* and 7 extracytoplasmic function (σ^ECF^) sigma factors. ECFfinder web tool successfully assigned 6 σ^ECF^ sigma factors to one of the 43 phylogenetically distinct σ^ECF^ groups defined by Staron *et al* 2009 (4).

**Operons encoding sigma factors in the genome of *F. psychrophilum* OSU THCO2-90.**

| **Locustag** | **Protein ID** | | **Start** | | **End** | **Name** | **Annotation** | | **ECFfinder** | |
| --- | --- | --- | --- | --- | --- | --- | --- | --- | --- | --- |
| THC0290_0666 | FP2048 | 803319 | | 804182 | | *rpoD* | | Primary sigma-70 sigma factor |  |  |
| THC0290_2206 | FP2249 | 2555365 | | 2556831 | | *rpoN* | | RNA polymerase sigma-54 factor |  |  |
| THC0290_0638 | FP2077 | 772469 | | 773029 | |  | | RNA polymerase ECF-type sigma factor | ECF21 |  |
| THC0290_0639 | FP2076 | 773016 | | 773693 | |  | | Putative anti ECF-type sigma factor |  |  |
| THC0290_0640 | FP2075 | 773726 | | 774790 | |  | | Putative sensor of anti-sigma and ECF sigma factor |  |  |
| THC0290_0781 | FP1933 | 929993 | | 930601 | |  | | Putative anti ECF-type sigma factor |  |  |
| THC0290_0782 | FP1932 | 930615 | | 931109 | |  | | RNA polymerase ECF-type sigma factor | ECF22 |  |
| THC0290_1586 | FP0623 | 1842055 | | 1842597 | |  | | RNA polymerase ECF-type sigma factor | ECF03 |  |
| THC0290_1587 | FP0622 | 1842628 | | 1843008 | |  | | Putative anti ECF-type sigma factor |  |  |
| THC0290_1588 | FP0621 | 1843019 | | 1843477 | |  | | Putative sensor of anti-sigma and ECF sigma factor |  |  |
| THC0290_1653 | FP0549 | 1917528 | | 1918436 | |  | | Putative anti ECF-type sigma factor |  |  |
| THC0290_1654 | FP2508 | 1918439 | | 1918939 | |  | | RNA polymerase ECF-type sigma factor | ECF21 |  |
| THC0290_1773 | FP1454 | 2056140 | | 2056589 | |  | | Putative anti ECF-type sigma factor |  |  |
| THC0290_1774 | FP1455 | 2056576 | | 2057085 | |  | | RNA polymerase ECF-type sigma factor |  |  |
| THC0290_1785 | FP1466 | 2068194 | | 2068742 | |  | | RNA polymerase ECF-type sigma factor | ECF01 |  |
| THC0290_1868 | FP1106 | 2168095 | | 2168679 | |  | | RNA polymerase ECF-type sigma factor | ECF01 |  |

For sigma factor genes that are transcribed as polycistronic mRNAs, all genes of the operon are listed.

All but one (THC0290_1785) are expressed in at least one of the condition-dependent dataset. While *rpoD* (σ^A^) is expressed at high level in all samples, its expression is highest in conditions such as freshwater survival or fish plasma exposure. In contrast, *rpoN* (σ^54^) appears as constitutively expressed. This is consistent with a putative post-transcriptional control of its activity as described for the σ^54^ family that requires enhancer-binding activators providing ATP for transcription initiation (5). The expression profiles of the 6 σ^ECF^-type factors follow a general pattern of increase in response to nutrient limitation (low nutrient agar, freshwater). However, they are not identical suggesting differentiated roles: dynamic range of induction is widest for THC0290_1586, and some factors are also induced in specific conditions such as plasma exposure for THC0290_1774 (and to a lower extent THC0290_0638), and high osmotic pressure for THC0290_1868.


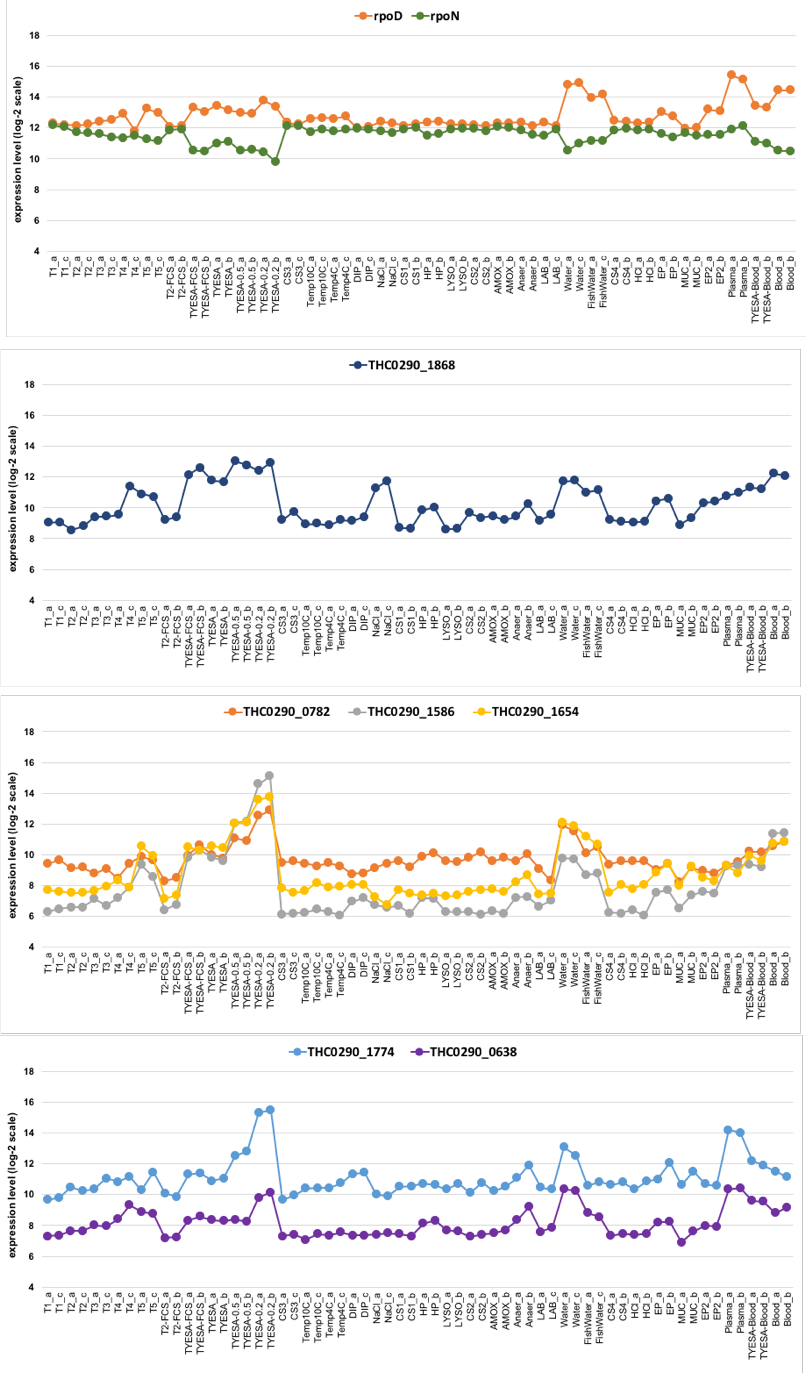


**Quantile-normalized log2-expression level of mRNAs encoding sigma-factors across 32 biological conditions in duplicates.**

# D5. Promoter sequence discovery and classification into sigma factor regulons.

The *de novo* prediction algorithm classified the TSSs as associated to occurrences of 6 distinct sigma factor binding site motifs, numbered from SM1 to SM6 according to their numbers of occurrences (**Table S2**).

***Primary sigma factor motifs.*** The first 3 motifs (SM1, SM2 and SM3) carry the TAnnTTTG consensus of the -7 box recognized by σ^A^ with 5’ and 3’ extensions whose composition and length differed between the 3 motifs. Compared to SM1, which represents the majority of promoters (808 TSSs), SM3 (110 TSSs) shows a shorter 5’ extension but with a more pronounced T-enrichment. Motif SM2 (177 TSSs) displays a C+A-rich 3’ extension and is also the only one that displays a TTTG box around position -33, suggesting that the presence of the -33 box is dispensable. The 3 motifs differed by the average transcription level measured downstream the corresponding promoters: this level was the highest for SM2 and the lowest, but also the most variable across conditions, for SM3.

***Alternative sigma factor motifs.***

**SM4:** Motif SM4 corresponds to the typical σ^54^ consensus sequence defined by the consensus TGGCACGnnnnTTGC (5). Heterogeneity between SM4 promoters was examined under the light of gene co-expression clusters (**Table S3**). Two clusters (B254 and B419) were over-represented among SM4 promoters associated genes. Genes belonging to cluster B254 were expressed at a high basal level, up-regulated during growth on blood agar and down-regulated under nutrient starvation (stationary phase, freshwater, low-nutrient agar). This cluster contains 7 transcriptional units, out of which 5 were identified as transcribed from SM4 promoters. These encode the ubiquitous heat-shock inducible molecular chaperones DnaJ-DnaK-GrpE, GroELS and HtpG, and the ATP-dependent protease Lon responsible for misfolded protein degradation. Although no TSS was detected by 5’-end RNA-seq (and thus no sigma binding site identified) upstream of the two other genes belonging to this cluster (namely, *ftsH* and *secA*), it is remarkable that their functions are also related to quality control of proteins and to envelope stress response. Furthermore, other genes with related functions but outside of co-expression cluster B254, such as those encoding the outer membrane chaperone Skp (OmpH) and ClpB AAA+ disaggregase are also predicted to be transcribed from SM4 promoters. The second over-represented cluster B419 is composed of 34 transcriptional units, 13 of which were identified as transcribed from SM4 promoters. Genes in this cluster are up-regulated during growth on blood, but in contrast to cluster B254, they are also highly induced under osmotic pressure, plasma exposure and growth on low-nutrient agar. They encode proteins involved in gliding motility, pyrimidine and isoprenoid biosynthesis, as well as several exported proteins of unknown function.

**SM5:** No specific conditions of induction were identified based on average expression profiles, however SM5 promoters are found upstream of genes whose products are involved in specific functional pathways, such as sugars metabolism and amino-acids scavenging. In particular, SM5 promoters are found upstream genes encoding the lipopolysaccharide assembly protein LptE, the oligosaccharide translocase WzxE, a dTDP-4-amino-4,6-dideoxygalactose transaminase and the dTDP-4-dehydrorhamnose reductase RmlD (nucleotide sugar precursors synthesis), which link SM5 regulon to LPS biosynthesis. Genes downstream SM5 promoters also include the *remFG-sprCDBF* operon, as well as a LysE-type exporter related to amino-acids efflux systems, the Asp/Glu-specific dipeptidyl-peptidase Dpp11 and a glutamine cyclotransferase which catalyzes the cyclization of free L-glutamine and N-terminal glutaminyl residues in proteins (6-8).

**SM6:** Genes encoding several components of the T9SS are transcribed from SM6 promoters: the core machinery subunit GldN (PorN) transcribed from two promoters (SM6 and SM1); the peptidase PorU that cleaves the C-terminal secretion signal after translocation of T9SS substrates on cell surface (44); and THC0290_0621, an outer membrane protein of unknown function identified in *Porphyromonas gingivalis* (PG0189) as interacting with the PorK/PorN complex (45). Others proteins of unknown function are also part of the SM6 regulon, such as Band 7 domain proteins and the ATPase component of a putative ABC transporter (THC0290_1050), found specifically induced in very low-nutrient solid medium.

# D6. Two promoters control the expression of the gliding motility operon *remFG-sprCDBF*

The role of the motifs SM4 and SM5 identified in the region upstream the *remFG-sprCDBF* operon on the transcriptional initiation was evaluated in *F. psychrophilum* THCO2-90. A transcriptional reporter plasmid (pCP*Gm*^r^-P*_less_*-mCh) that allows fluorescence-monitoring of the activity of selected promoters in *F. psychrophilum* was constructed (***SI Appendix* M2**) and used in order to analyze the contribution of the SM4 and SM5 sigma factor binding sites. A high activity was measured when the 404 bp-long DNA region upstream *remF* was cloned upstream the mCherry gene. The introduction of transversion mutations *sm4** or *sm5** at highly conserved positions of SM4 and SM5 consensus resulted in a significant decrease of promoter activity and mutagenesis of both motifs s*m4***sm5** resulted in residual activity comparable to the promoter-less plasmid. These results confirm the role of the two TSSs to *remFG-sprCDBF* transcription and the importance of highly conserved nucleotides identified *in silico*.

# D7. Antisense transcription mainly originates from 3’ extended regions and non-coding RNAs.

We detected 287 TRs, hereafter named asRNAs, that overlap the antisense strand of 281 CDSs. A wide variety of sense/antisense configurations is found regarding the length (with 25% of asRNAs < 196 bp and 25% > 545 bp) and the location of asRNAs. Some are 3’UTRs extending tail-to-tail within adjacent CDSs, others are indep TRs partially or fully overlapping the coding region in sense.

An assessment of 3’-end boundaries and antisense transcription was performed using condition-dependent expression profiles. The boundaries of transcribed regions (TRs) were defined based on the RNA-seq coverage of a 18-condition RNA pool. As a consequence, the resulting annotation corresponds to the largest region transcribed under these 18 experimental conditions. Expression levels of 64 individual RNA samples showed that for many 3’ and indep TRs, the exact 3’ boundaries greatly differed between biological conditions and correlate with the mRNA expression level.


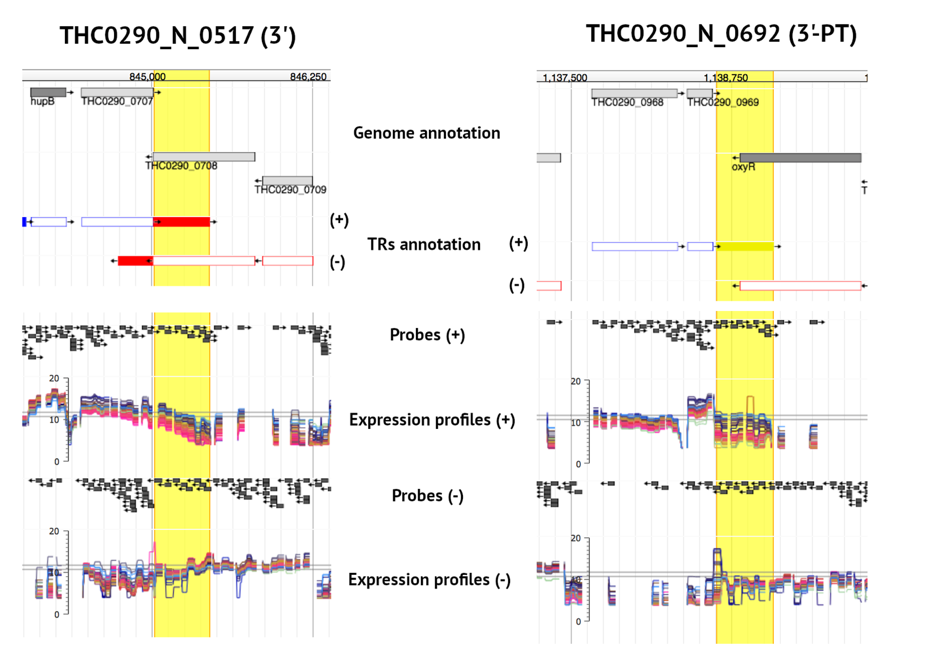

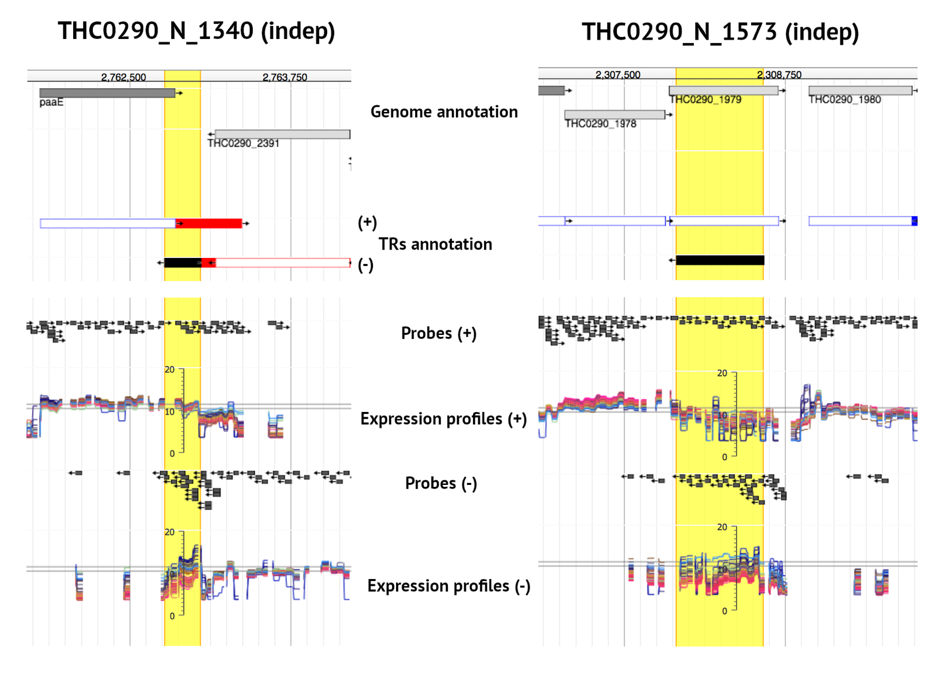


**Examples of asRNAs originating from 3' extended regions and Indep TRs.**

The region defined based on RNA-seq read coverage data is highlighted in yellow (“TRs annotation”). An overlay of the 64 microarray expression profiles is shown (one color by condition; log2-expression signal). The black horizontal lines correspond to 1X and 2X the median log2-expression signal for the whole chromosome, respectively.

**Conservation across the phylum *Bacteroidetes* and secondary structure of Indep TRs which are antisense of CDSs.**

Heatmap representation of conservation profile for the Indep TRs identified in this study which are antisense of annotated CDSs and RFAM RNAs. Length and evidence for secondary structure (low Minimum Free Energy z-score indicates significant folding) are represented as bar-plots on the right-side of the heatmap.

# D8. 5’ TRs belonging to known *cis-*regulatory RNA families*.*

TRs detected in this study confirm the expression of 5 predictions made by scanning the *F. psychrophilum* THCO2-90 genome for RNA features known in other species, based on RFAM (9) (**Table S7**). A cobalamin riboswitch was identified upstream of THC0290_1776 mRNA which encodes an uncharacterized TonB-dependent transporter (TBDT) up-regulated under fish plasma exposure and located in the vicinity of the *cob* genes of adenosyl cobalamin biosynthesis. Two thiamine pyrophosphate (TPP) riboswitches were identified, respectively upstream of *thiSCE1DE2GHF* operon (thiamine biosynthesis pathway) and THC0290_2296 mRNA, which encodes an uncharacterized TBDT, suggesting a role in the uptake of thiamine or a related compound. These two mRNAs, which are not genetically linked, are co-expressed (gene cluster B209), confirming that they are subject to the same transcriptional control: their transcript levels increase concomitantly during transition from exponential to stationary phase and under nutrient deprivation. A SAM-II riboswitch that binds S-adenosyl-L-methionine (SAM) was found in the 5’UTR of *metK* mRNA encoding methionine adenosyltransferase (SAM biosynthesis). Finally, a *Bacteroidetes* tryptophan peptide leader RNA, previously identified *in silico* in genomes of *Bacteroidetes* species (10), was mapped upstream of the *trpE-pabA-trpDCFBA* operon indicating post-transcriptional control of tryptophan biosynthesis by uncharged tRNA^Trp^ availability as already described for other bacteria (11).

# D9. New 5’ *cis*-regulatory RNAs candidates

**Leader peptide attenuation elements.**

In-depth sequence analysis of the 64 structured 5’ TRs indicates leader peptide-mediated transcriptional attenuation mechanism for several of them (**Table S7**). In particular, a 25-aa long CDS containing 8 isoleucine residues was predicted within the long 5’ structured TR of *ilvE* mRNA (branched-chain AA synthesis), strongly suggesting a control mechanism by leader peptide attenuation. Similarly, considering (i) prediction of short CDSs with tandem-arranged aromatic amino-acids in the 5’ region, (ii) transcriptional read-through observed under specific conditions, and (iii) co-expression of downstream genes, the expression of *hisG*, *pheA* and *trpE* operons encoding enzymes of the aromatic amino-acids biosynthetic pathways are probably also controlled in *F. psychrophilum* by leader peptide attenuation.


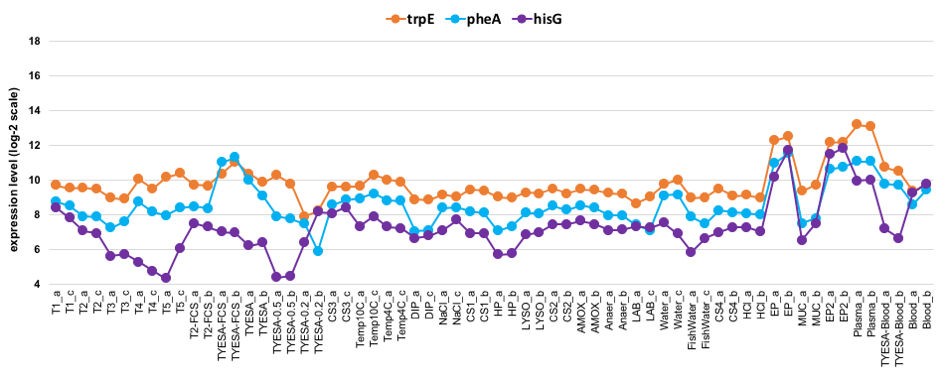


**Expression of regulated genes across biological conditions.**

Log2-expression level of the first gene of *hisG*, *pheA* and *trpE* operons across 64 individual RNA samples. All genes of these operons were co-expressed across conditions and are part of gene cluster C53. Maximal transcription levels were observed when cells are in minimal nutrient media.


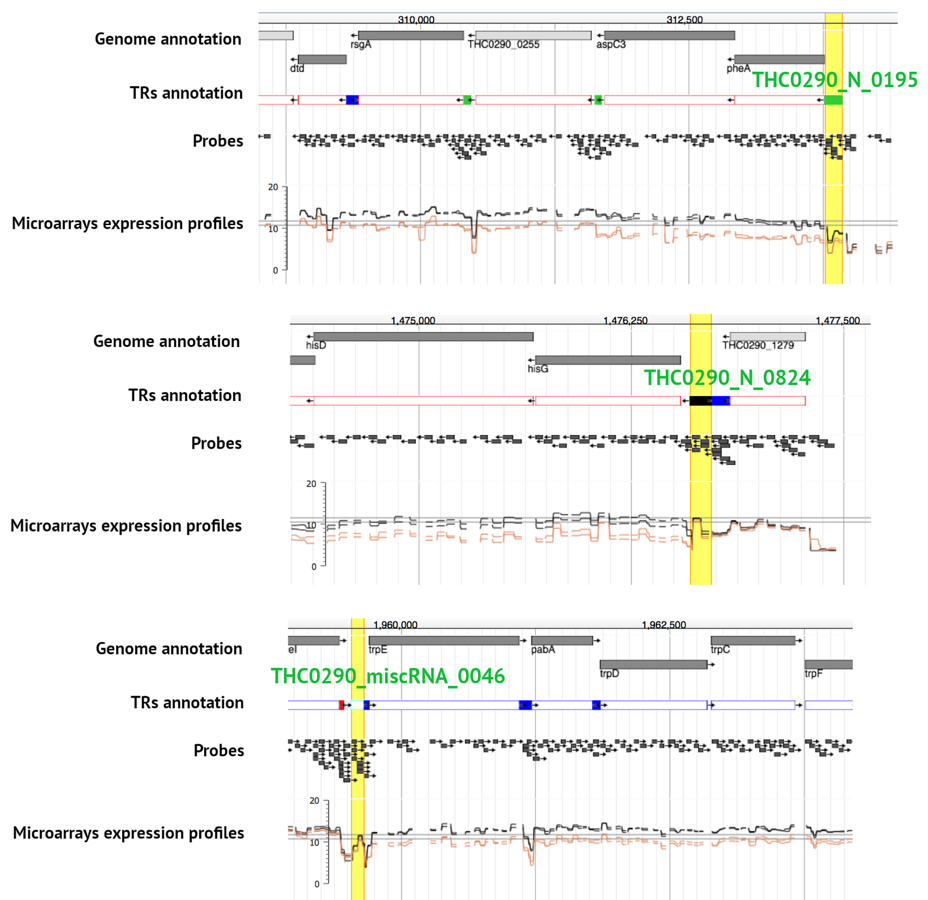


**Genomic view of three operons encoding enzymes for the aromatic amino acids biosynthesis pathways.**

Selected expression profiles are representative of two conditions with differential expression of the three operons (expression profiles colors are: black, cells resuspended in 1% peptone water only; brown, cells resuspended in 1% peptone water with fish mucus). Probable leader peptide 5’ RNA elements are highlighted in yellow.

*ilvE* operon: MRKFN**II**PS**III**A**I**V**II**TTAKGML (25 amino-acids)

*trpE* operon: MNTFLNNT**WWW**NNLRQTS (18 amino-acids)

*pheA* operon: MNTNNT**FY**SNNS**FYFY**WNIGRDC**Y**MVI (27 amino-acids)

*hisG* operon: MSLNQL**HHHHFH**ICSQAKC (19 amino-acids)

**Sequences of the short peptides containing tandem-arranged aromatic amino-acids within the 5’ regions of the aromatic amino-acids biosynthetic pathways operons.**


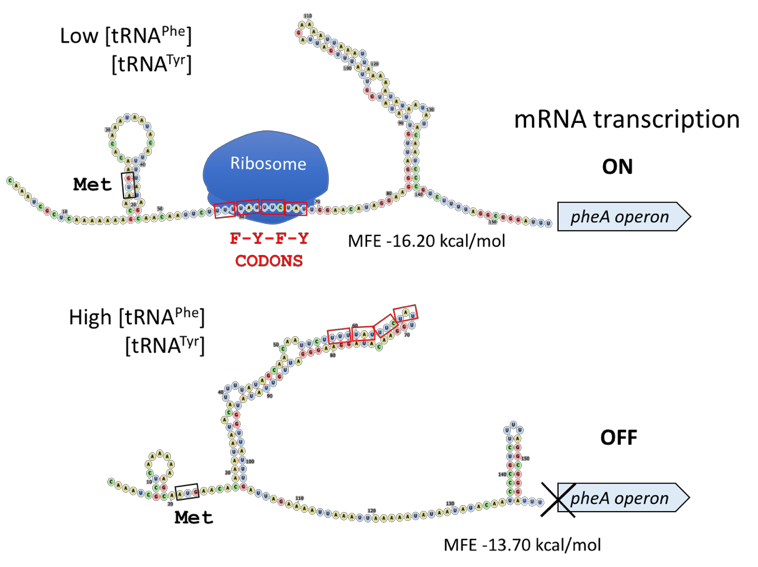


**Putative terminator/anti-terminator hairpins in the leader *cis*-regulatory element of *pheA* operon.**

Secondary RNA structures predicted by RNAfold and visualized using *forna* tool (the Vienna web services, TBI university).

**5’ untranslated regions.**

Structured 5’UTRs were identified for many mRNAs (**Table S7**). This is the case for instance in 4 mRNAs encoding aminoacyl-tRNA-synthetases (*alaS, proS, hisS* and *valS*) which is fully consistent with control of aminoacyl-tRNA-synthetases described in many bacteria by mechanisms as diverse as riboswitches (*eg*. T-box), autoregulation by binding of aminoacyl-tRNA-synthetase to its own 5’UTR, and transcriptional attenuation (12). Two ‘indep’ TRs were detected upstream of *metY* operon (homoserine biosynthesis) and may constitute a *cis*-regulatory element, but the exact boundaries were not correctly resolved. Other genes preceded by structured 5’UTRs include *acsA* (acetyl-CoA synthetase), *fumC* (fumarase; TCA cycle), *gpmI* (phosphoglycerate mutase), *ftsY* (signal recognition particle receptor involved in protein translocation), *clpP-clpX* (ATP-dependent protease complex playing a pleiotropic role by degrading abnormal proteins and mediating the turnover of some regulatory proteins), *ltaE* (threonine catabolism), a secreted subtilisin-family serine endopeptidase or the σ^ECF^ factor encoded by THC0290_1868. Several other exceptionally long UTRs (> 200 bp) may contain functional structured parts not identified due to the MFE cut-off established for the whole length of the TR. Among them are *hutU* (histidine utilization), *prfB* (peptide chain release factor 2 involved in translation termination), *wbpM* involved in UDP-D-Qui2NAc biosynthesis (O-antigen biosynthesis), *speA* (spermidine synthesis) and *pyrE* (pyrimidine synthesis).

A first information on the role of these 5’ regulatory elements can be suggested by condition-dependent expression profiles. For instance, aromatic amino-acids biosynthesis operons controlled by leader peptides are induced when cells are in minimal nutrient media or THC0290_0102 mRNA encoding a conserved exported protein of unknown function is down-regulated under cold temperature and high osmotic conditions (see below).


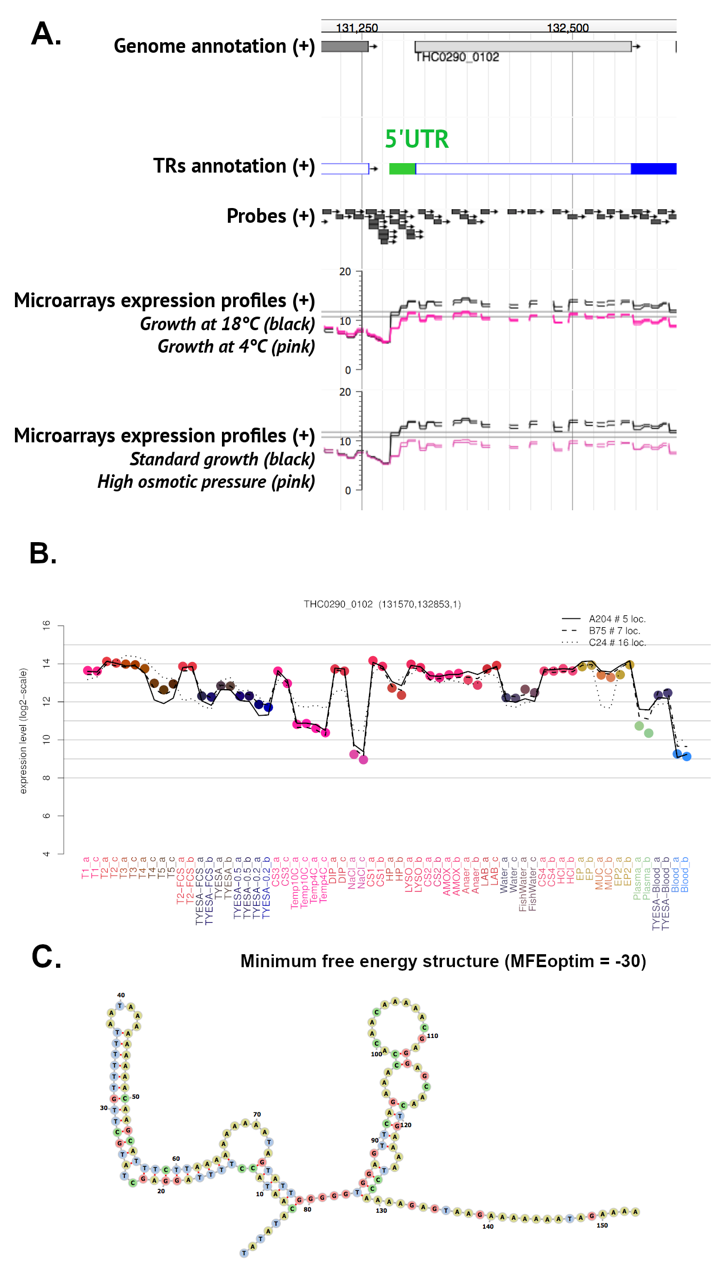


**Structured 5’ TR of THC0290_0102 mRNA encoding a conserved exported protein of unknown function down-regulated at low temperature and high osmotic pressure.**

A. Genomic view of the microarray expression profiles in bacterial cultures growing at 18°C or 4°C (upper panel) and under high osmotic pressure (0.75% NaCl) or not (lower panel). B. Log2-expression level of the THC0290_0102 mRNA across 32 biological conditions. C. Secondary RNA structure of the 152 nt-long 5’ UTR of THC0290_0102 mRNA predicted by RNAfold and visualized using *forna* tool (the Vienna web services, TBI university).

# D10. Small regulatory RNAs

A strong secondary structure was predicted for 18 of the 45 Indep TRs not listed as antisense (*versus* only 7 for the 40 TRs listed as antisense). The minimum free energy (MFE) structure was calculated using RNAfold and results visualized with the *forna* tool using the ViennaRNA Web Services (13). MFEoptim is the MFE optimal secondary structure with corresponding energy and Zscore (corrected for difference in nucleotide compositions between sequences).

**
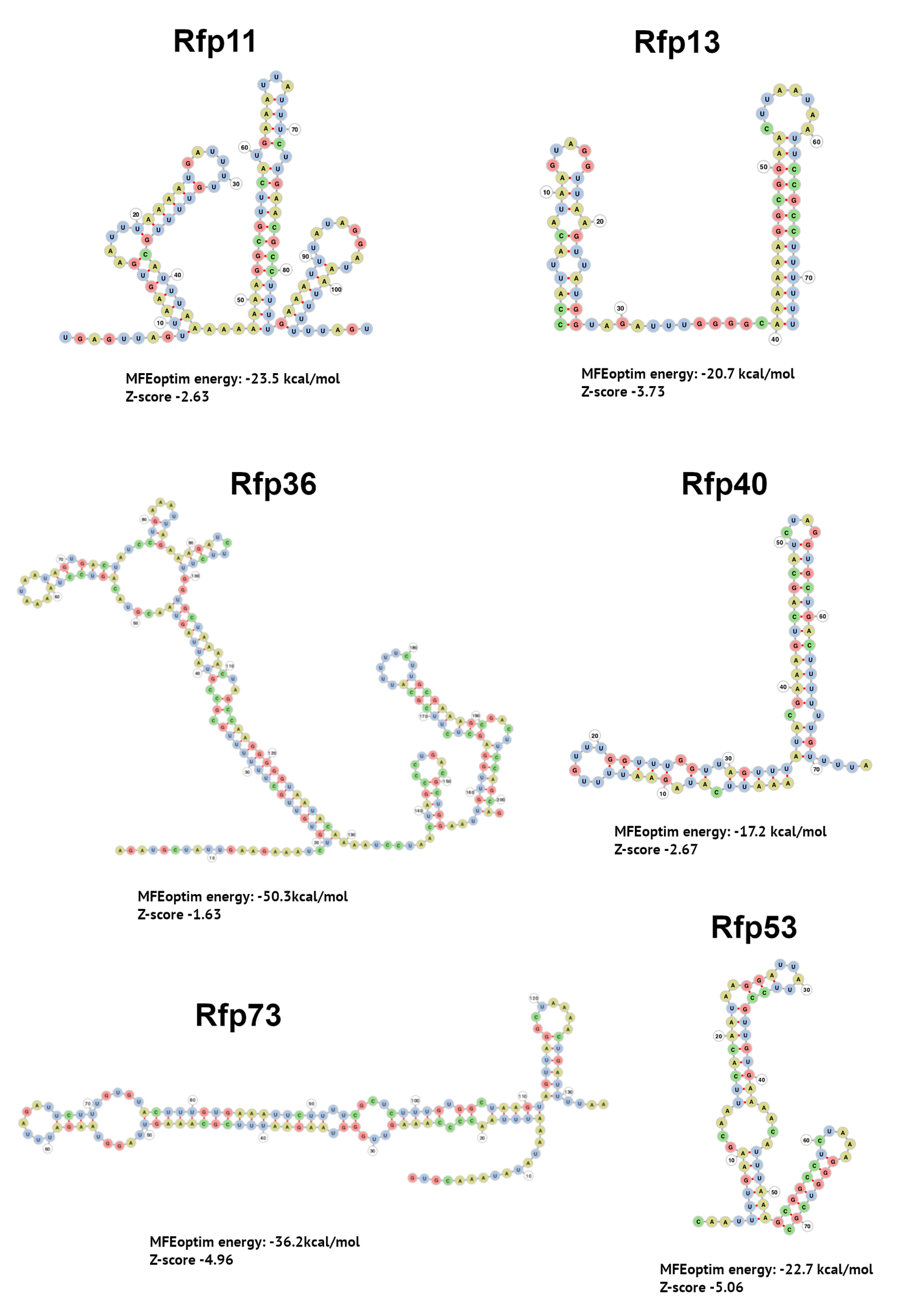
**

**Examples of Indep TRs with predicted secondary structures.**

The level of many sRNAs increases from exponential growth to stationary phase (27 and 67 are induced at T3 and T5, respectively, relative to T1), such as the 6S RNA, a global transcriptional regulator acting by direct binding to RNA polymerase (14). Many indep TRs with unknown function are induced in freshwater (*e.g*. Rfp25 and Rfp68), in colonies growing on low-nutrient medium (*e.g*. Rfp11), or on blood as sole nutrient source (*e.g*. Rfp53). For this last condition, 3 indep TRs are transcribed from a σ^54^ factor promoter (*i.e*. Rfp1, Rfp36 and Rfp45).


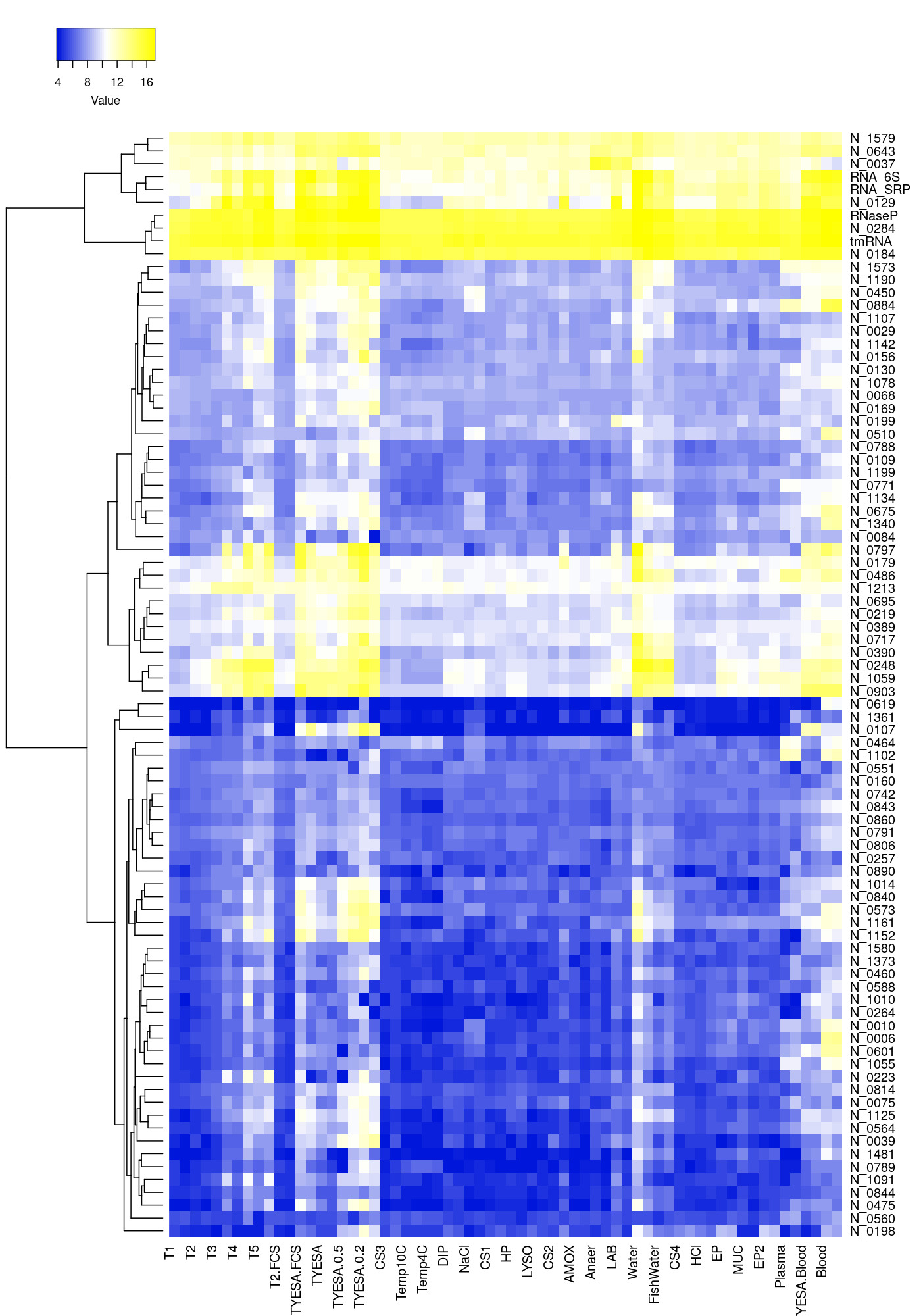


**Heatmap of hierarchical clustering of expression values across biological conditions of indep TRs and ubiquitous RNAs.**

Values correspond to quantile-normalized log2-expression values of 64 hybridizations (two biological replicates for each of the 32 biological conditions, full description in Table S1). Hierarchical clustering was performed using Heatmapper (15) with Manhattan distance and average linkage parameters.

# D11. Rfp18, a putative regulatory sRNA predicted to control several metalloproteases.

The putative sRNA Rfp18 (THC0290_N_0179) is conserved outside the species *F. psychrophilum* and expressed from a SM1 promoter (σ^A^). and likely folds into a strong secondary RNA structure composed of 4 stem-loops, the fourth likely acting as intrinsic terminator.

**
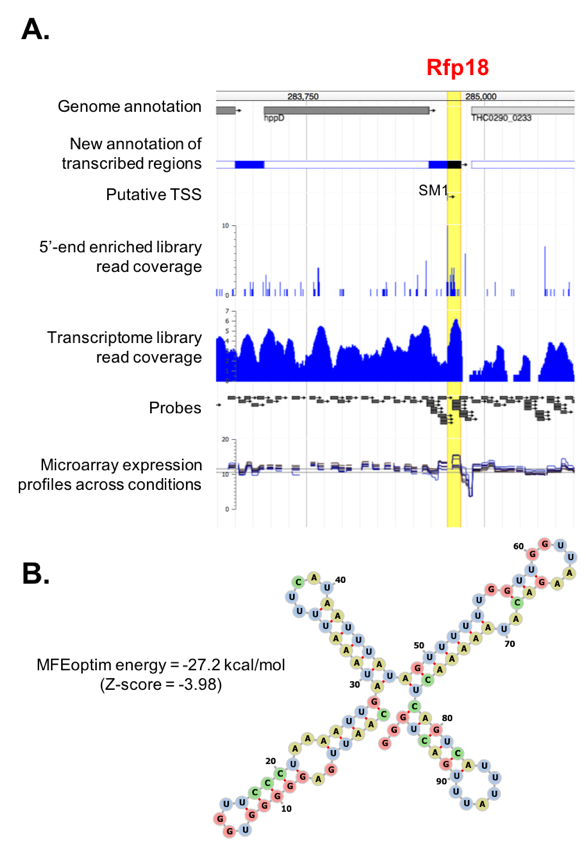
**

**Genomic view of the region surrounding Rfp18.**

SM1 indicates the TSS with predicted σ^A^ (SM1) binding site. Rfp18 is highlighted in yellow.


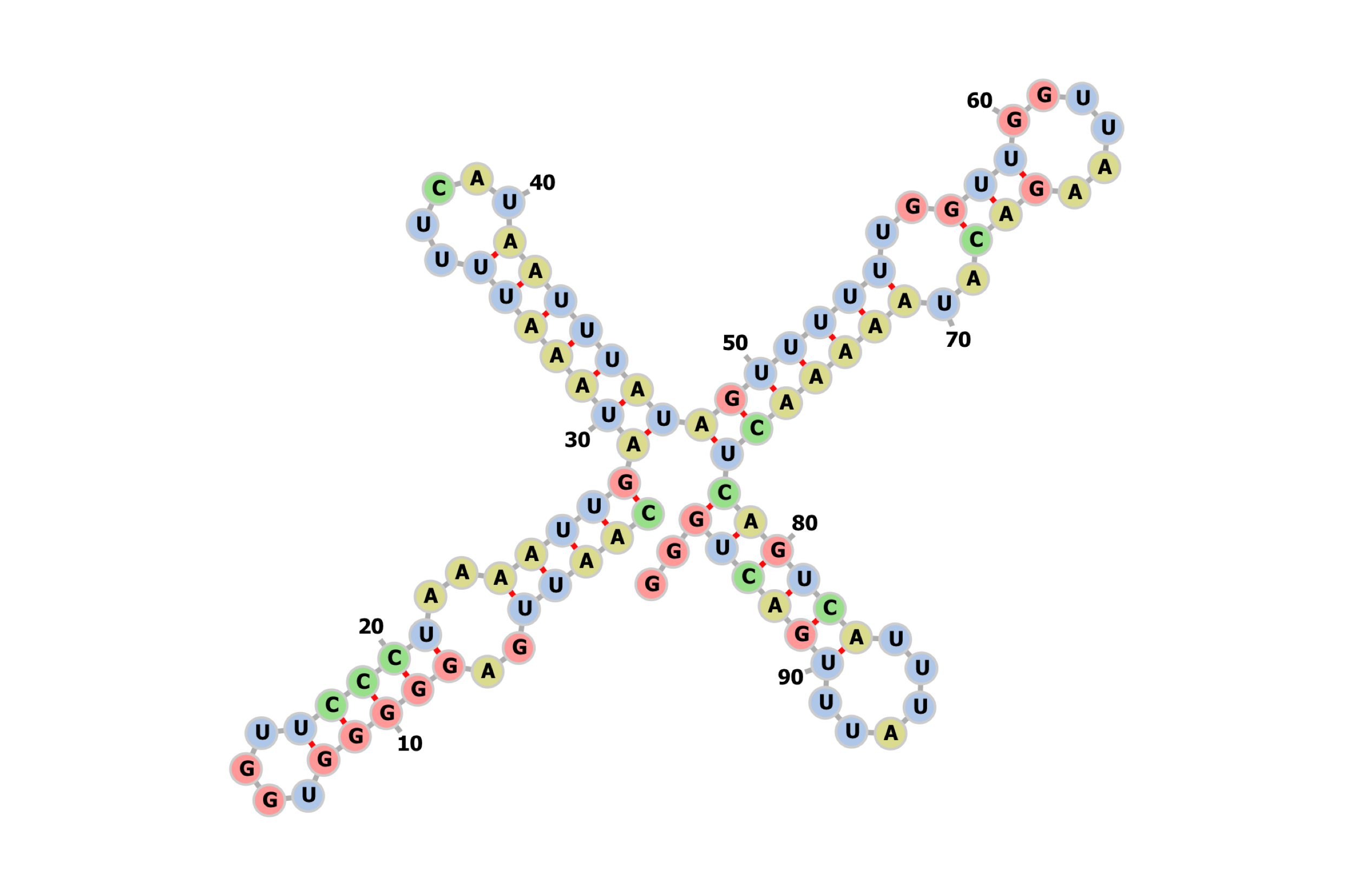


MFEoptim energy = -27.2 kcal/mol

(Z-score = -3.98)

**Representation of the Rfp18 secondary structure predicted by RNAfold.**

Target mRNA-sRNA interactions were examined to identify putative targets using CopraRNA with default parameters and 9 sequence homologs of Rfp18 as input (16). Searches were run on strain JIP02/86 instead of OSU THCO2-90 due to genome availability on the Freiburg RNA tools. Among the 13 putative metalloprotease-encoding mRNAs identified in strain JIP02/86 (17), 4 are predicted as possible targets: FP1619 (THC0290_1090) encoding a probable M43 cytophagalysin (1^st^ rank in the list of CopraRNA-predicted targets), FP0506 (3^rd^ rank, no homolog in strain OSU THCO2-90) encoding a metalloprotease with elastinolytic activity (18), FP0231 (THC0290_0237) encoding the psychrophilic metalloprotease Fpp1 (20^th^ rank) and, with lower confidence, FP0281 (THC0290_0300; 102^th^ rank) encoding a probable M36 fungalysin family metalloprotease. Another high confident predicted target encodes a putative adhesin FP2413 (THC0290_2338, 2^nd^ rank) secreted through the T9SS (19). The predicted pairing region of Rfp18 was identical for the above-mentioned putative targets, with the involvement of a GGUUGG seed motif between positions 56 and 61 of the third stem-loop.

**FP1619 (THC0290_1090)** probable M43 cytophagalysin family metalloprotease

IntaRNA Energy = -17.40 kcal/mol ; CopraRNA p-value = 2.33E-07

**176 208**

**| |**

**5'-AAU...UAACA CC AC AAUUA...CAC-3’ mRNA**

**AACCAACC AAAAUUA AAAUUAUGAAAA**

**|||||||| ||||:|| ||||||||||||**

**UUGGUUGG UUUUGAU UUUAAUACUUUU**

**3'-GGG...CAGAA UU A AAAUA...AAC-5’ Rfp18**

**| |**

**64 33**

**FP02413 (THC0290_2338)**, putative adhesin

IntaRNA Energy -11.06 kcal/mol ; CopraRNA p-value = 1.10E-05

**175 208**

**| |**

**5'-CAA...AAAUC AAU UAAC AAAUU...CCA-3' mRNA**

**AAUCAACCAAAAAA UGA UAUGAAAA**

**||:||||||||||| |:| ||||||||**

**UUGGUUGGUUUUUU AUU AUACUUUU**

**3'-GGG...CAGAA GAU UA AAAUA...AAC-5' Rfp18**

**| |**

**64 33**

**FP0506**, metalloprotease with elastase activity

IntaRNA Energy -15.6 kcal/mol ; CopraRNA p-value = 8.88E-05

**182 205**

**| |**

**5'-ACU...UAAAA C CAACA...CUG-3’ mRNA**

**UUAACCAACC AAAAAUUAUGA**

**|||||||||| |||||:|||:|**

**AAUUGGUUGG UUUUUGAUAUU**

**3'-GGG...UACAG U UAAUA...AAC-5’ Rfp18**

**| |**

**66 43**

**FP0231 (THC0290_0237)**, psychrophilic metalloprotease Fpp1

IntaRNA Energy -12.44 kcal/mol ; CopraRNA p-value = 9.98E-04

**174 208**

**| |**

**5'-AAC...AAAAU U CUU C AG AAAAA...CAA-3’ mRNA**

**UUAACCAACC AAA CU UAAA AUGAAAA**

**|||||||||| ||| || |||| |||||||**

**AAUUGGUUGG UUU GA AUUU UACUUUU**

**3'-GGG...UACAG UUU U AA AAAUA...AAC-5’ Rfp18**

**| |**

**66 33**

**FP0281 (THC0290_0300)**, probable M36 fungalysin family metalloprotease

IntaRNA Energy -8.13 kcal/mol ; CopraRNA p-value = 6.02E-03

**167 208**

**| |**

**5'-UUU...AUAUA AA UUUGA UA AAACU...GUU-3’ mRNA**

**UUUGUG UAAUUAACCAAAA UAUAA AUGAAAA**

**|||:|| |||::|||||||| ||||| |||||||**

**AAAUAC AUUGGUUGGUUUU AUAUU UACUUUU**

**3'-GGG...CUCAA AGA UUG UAA AAAUA...AAC-5’ Rfp18**

**| |**

**74 33**

**Selection of predicted mRNA-sRNA interactions.**

**
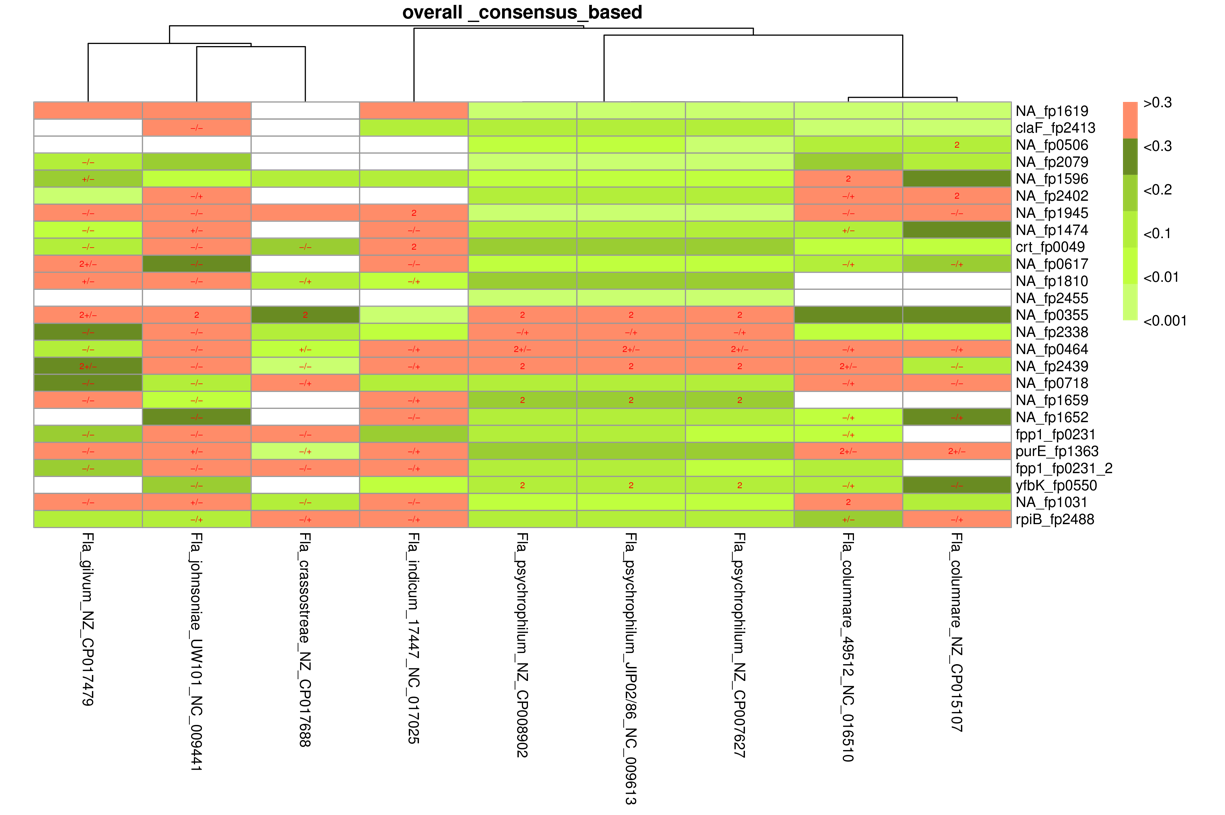
**

**CopraRNA graphical output of the predicted targets of Rfp18.**

The heatmap shows the interaction conservation of the top 25 predictions. Full results are available in **Table S9**.

A role of Rfp18 in the regulation of predicted mRNA targets was investigated by constructing a deletion mutant of *rfp18* in *F. psychrophilum* OSU THCO2-90. Absence of Rfp18 did not affect the growth or the proteolytic activity on spent medium at three stages of growth (OD 0.5, 1.2 and 2) using azocasein as substrate.

**Characterization of *F. psychrophilum* Δ*rfp18* strain.**

**A.** Growth monitoring of wild-type (WT) and Δ*rfp18* in TYES broth at 18°C. **B.** proteolytic activity on spent medium at three stages of growth (OD 0.5, 1.2 and 2) using azocasein as substrate. Data presented are mean and SD from three independent cultures.

The expression of Rfp18 homolog was confirmed in *Flavobacterium columnare* by PCR amplification using specific primers (***SI Appendix* T2**) and cDNA synthetized from total RNA extracts as described in ***SI* *Appendix* M12**.

**
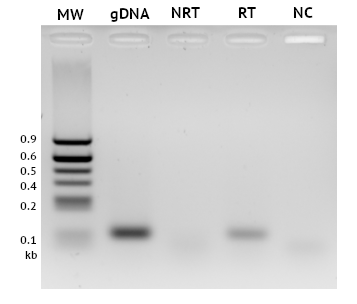
**

**Rfp18 homolog is transcribed in *F. columnare.***

Migration of the products after RT-PCR: MW: pBR322 DNA/AluI marker (Fermentas); gDNA: positive control (genomic DNA used as template); NRT: negative control (cDNA synthesis reaction without reverse transcriptase used as template); RT: cDNA reaction used as template; NC: no DNA template.

# D12. Transcriptional adaptation of freshwater-living bacteria

Pairwise comparisons between bacteria maintained in freshwater with or without rainbow trout relative to growth in TYES broth identified large numbers of differentially expressed genes (1029 DEG for freshwater relative to TYES condition) and PCA analysis pointed out similarities (75% shared DEG) between transcriptional profiles of cells in freshwater and in stationary phase (***SI* *Appendix* T4**), testifying of the nutritional deprivation of *F. psychrophilum* outside the host.

Among genes highly differentially expressed in freshwater are elements of clusters B549 (30), C140 (11), C50 (2) that are up-regulated, and C107 (41) that is down-regulated. Expression profiles of a selection of genes belonging to these clusters are shown below. The full list of genes constituting clusters is available on the ***fpeb*** website and **Table S4**.

Among those genes, some were up-regulated in freshwater only in the presence of rainbow trout (see ***SI Appendix* D13**).

Genes related to redox homeostasis were up-regulated in freshwater: an operon conserved in *F. columnare* encoding an AhpC-like peroxiredoxin (THC0290_1514) and 4 unknown function proteins*,* the bi-functional catalase-peroxidase KatG (see below gene cluster B549).


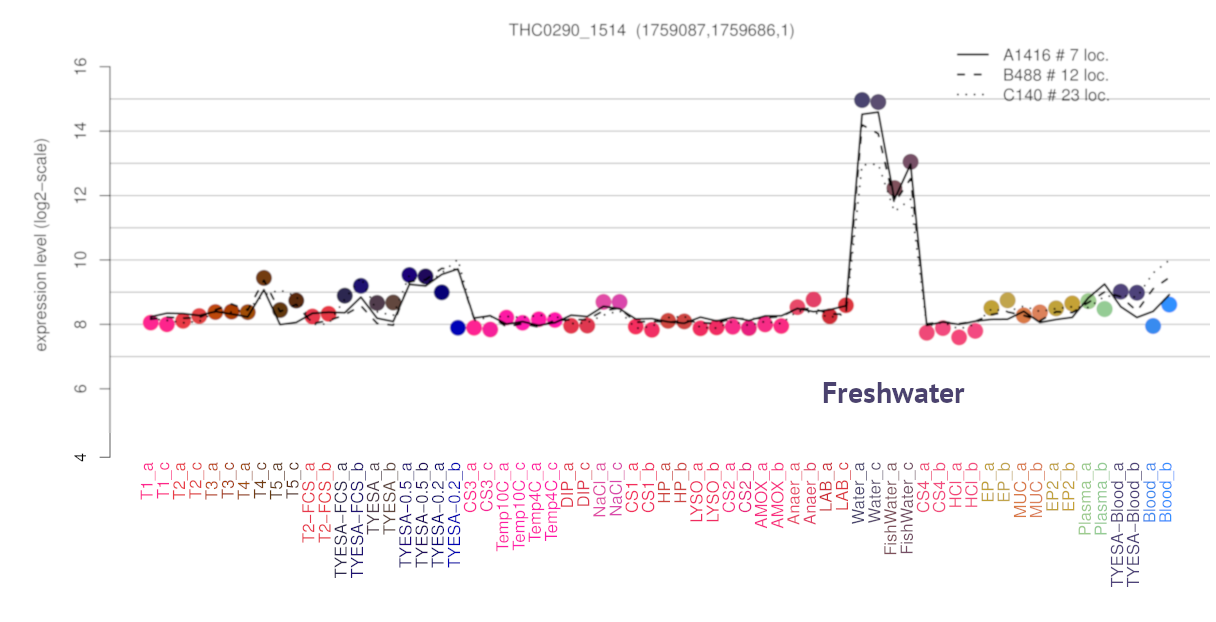


**Expression profiles of a representative gene of cluster C140 upregulated in freshwater-living bacteria.**

Quantile-normalized log2-expression values across 64 RNA samples

Several peptidases are among the freshwater-induced genes, indicating that *F. psychrophilum* produces enzymes necessary for harvesting host proteins and/or for tissue invasion when outside the host. Some such as the collagenase are up-regulated in freshwater independently of the presence of rainbow trout, while others like *fpp1* accumulate in the presence of fish.


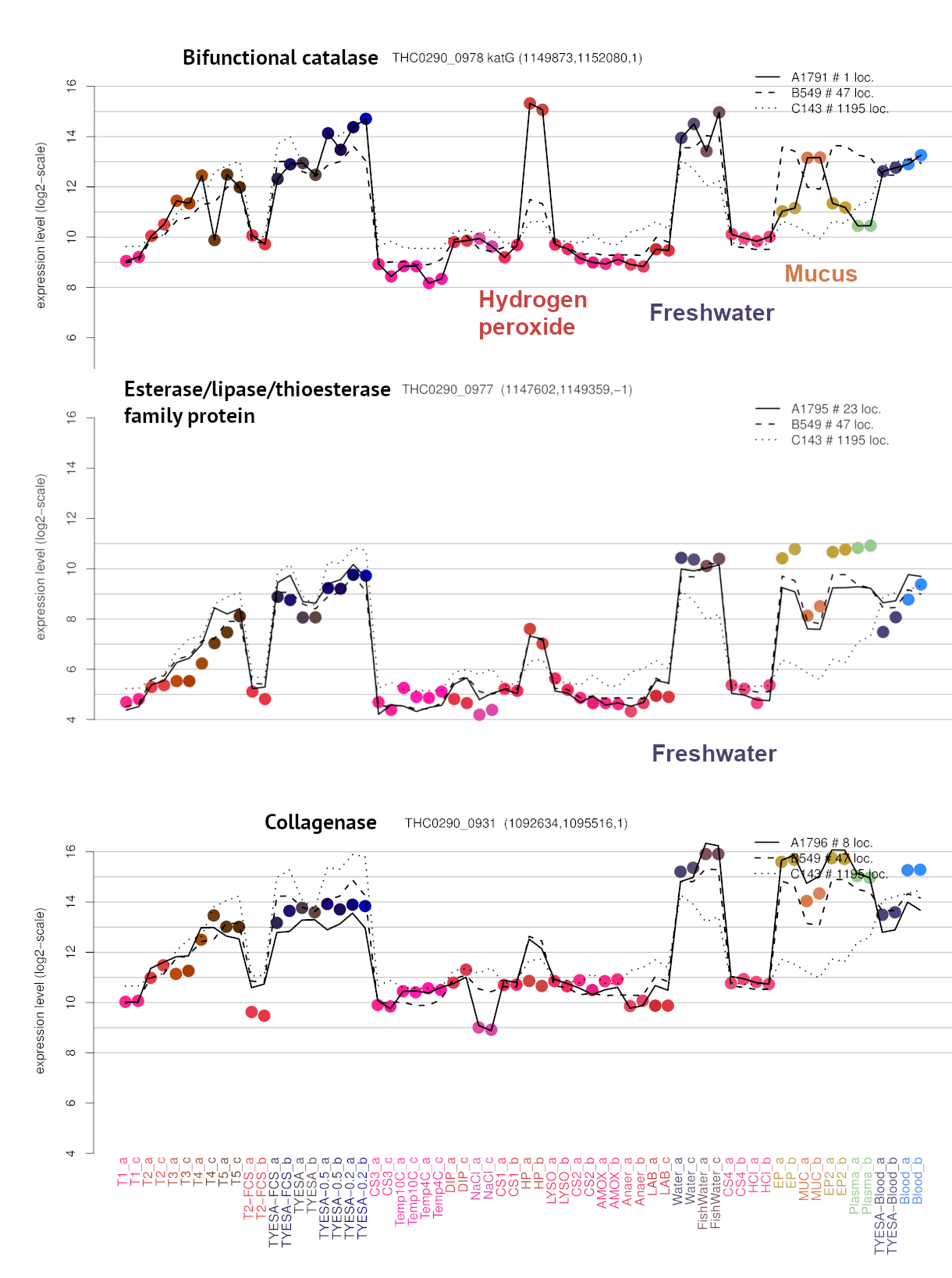


**Expression profiles of two genes representative of cluster B549 upregulated in freshwater-living bacteria.**

The iron storage ferritin encoding gene *ftnA* is induced in freshwater-living bacteria while genes related to iron acquisition (gene cluster C107), including several TBDTs (*e.g*. FecA, FhuA or a putative heme receptor) are repressed.


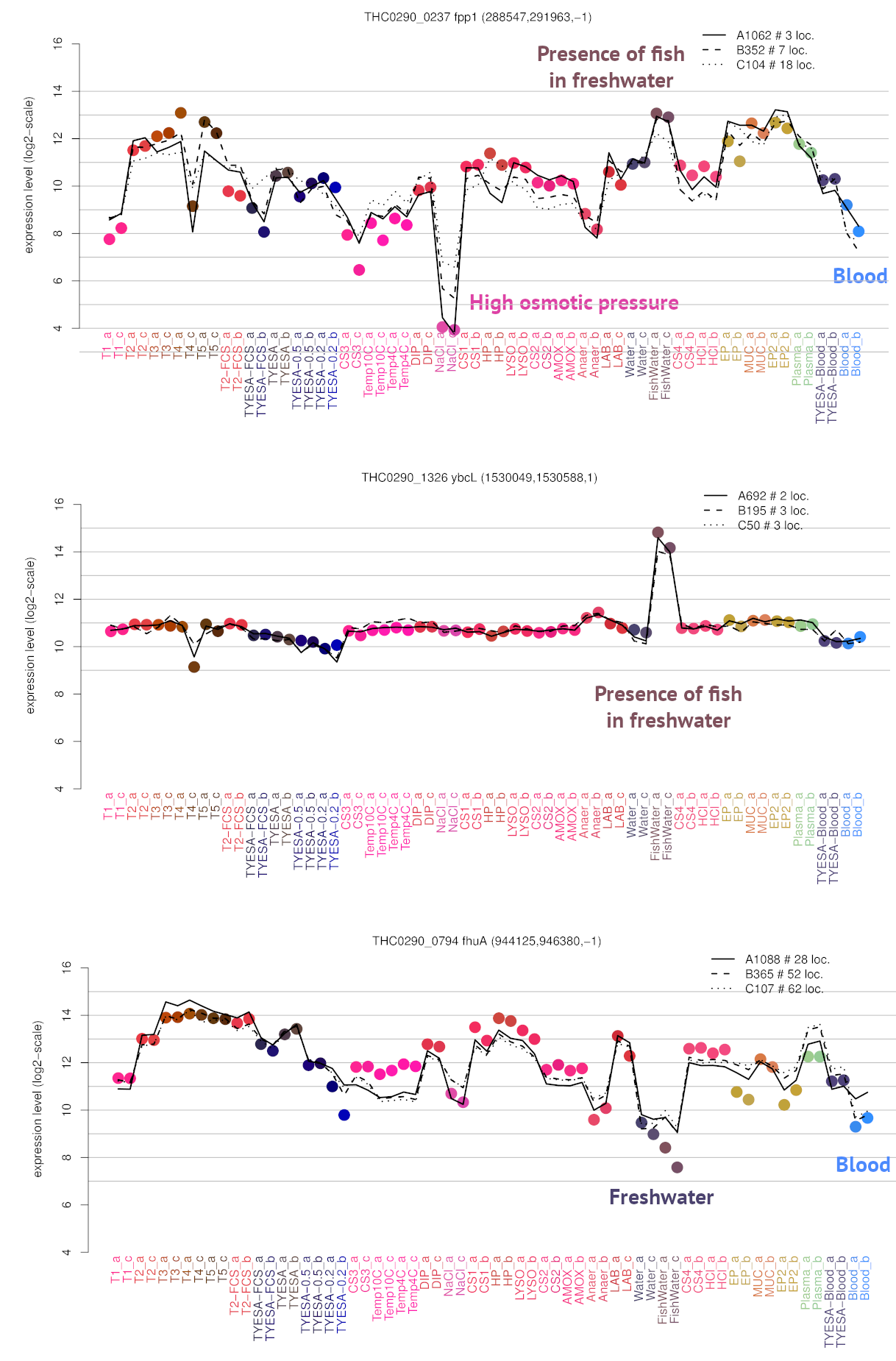


**Expression profile of a representative gene of cluster C107 down-regulated in freshwater-living bacteria.**

# D13. Responses to fish compounds

Transcriptional changes specifically associated to the host were investigated using three pairwise comparisons, namely the presence of fish relative to freshwater only, exposure to fish mucus or fish plasma.

In contrast to the collagenase, Fpp1, Fpp2 and THC0290_0300 metalloproteases are induced in freshwater-living cells only when fish are present (cluster C104).


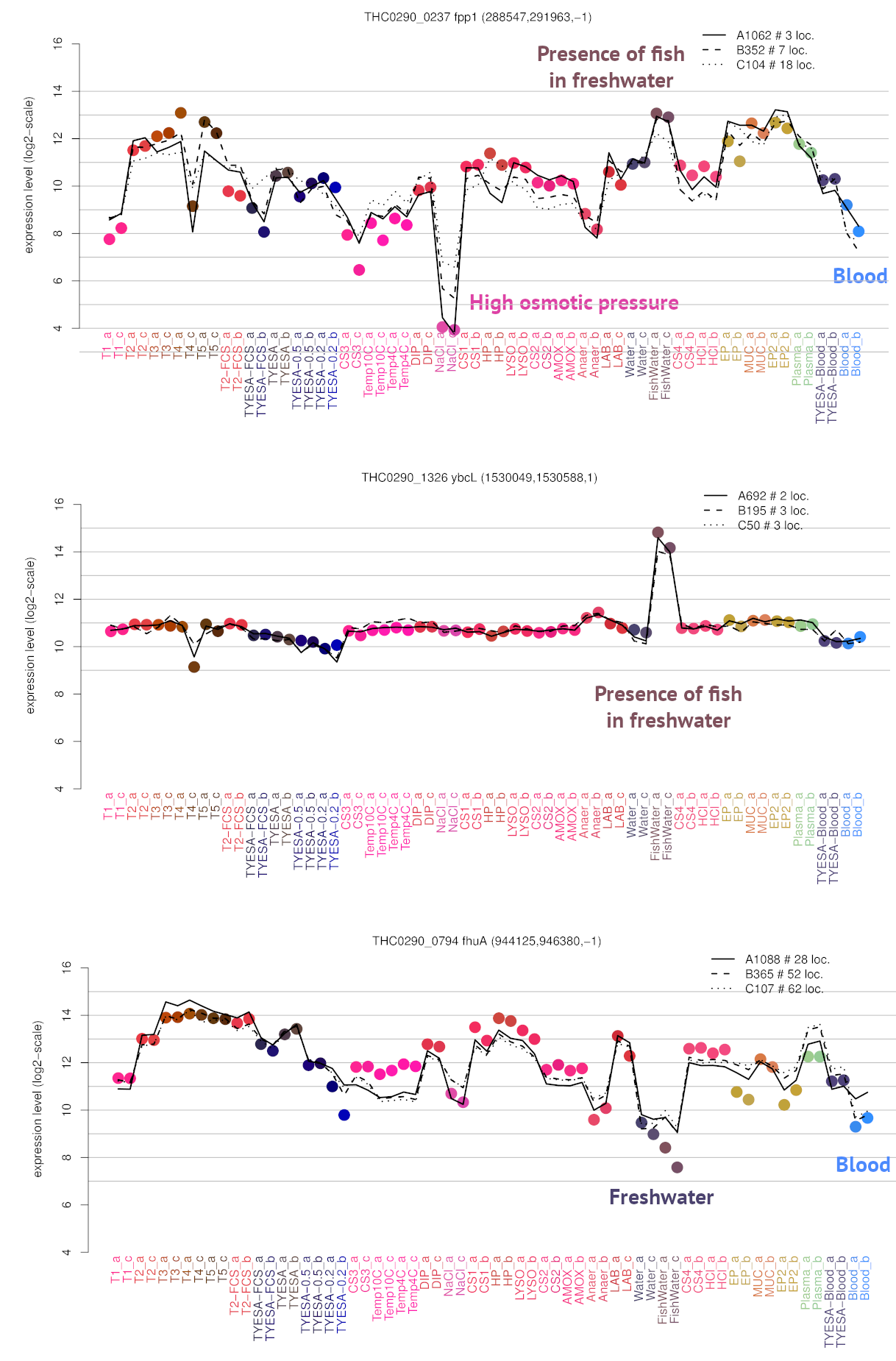


**Expression profile of *fpp1* from cluster C104 upregulated in freshwater only in the presence of fish.**

Among the 59 CDSs differentially expressed in freshwater in the presence of fish, a bicistronic operon encoding a phosphatidylethanolamine-binding family protein (YbcL) and a protein of unknown function related to cytochromes (HTHP_sf domain) is expressed only in the presence of rainbow trout compared to all other conditions (cluster C50). In *F. psychrophilum* and *F. columnare*, YbcL is conserved in synteny with a putative AraC-type transcriptional regulator that may switch on *ybcL* expression by sensing a fish metabolism-derived compound (20). A YbcL ortholog was found to inhibit neutrophil migration in uropathogenic *E. coli* strains (21).


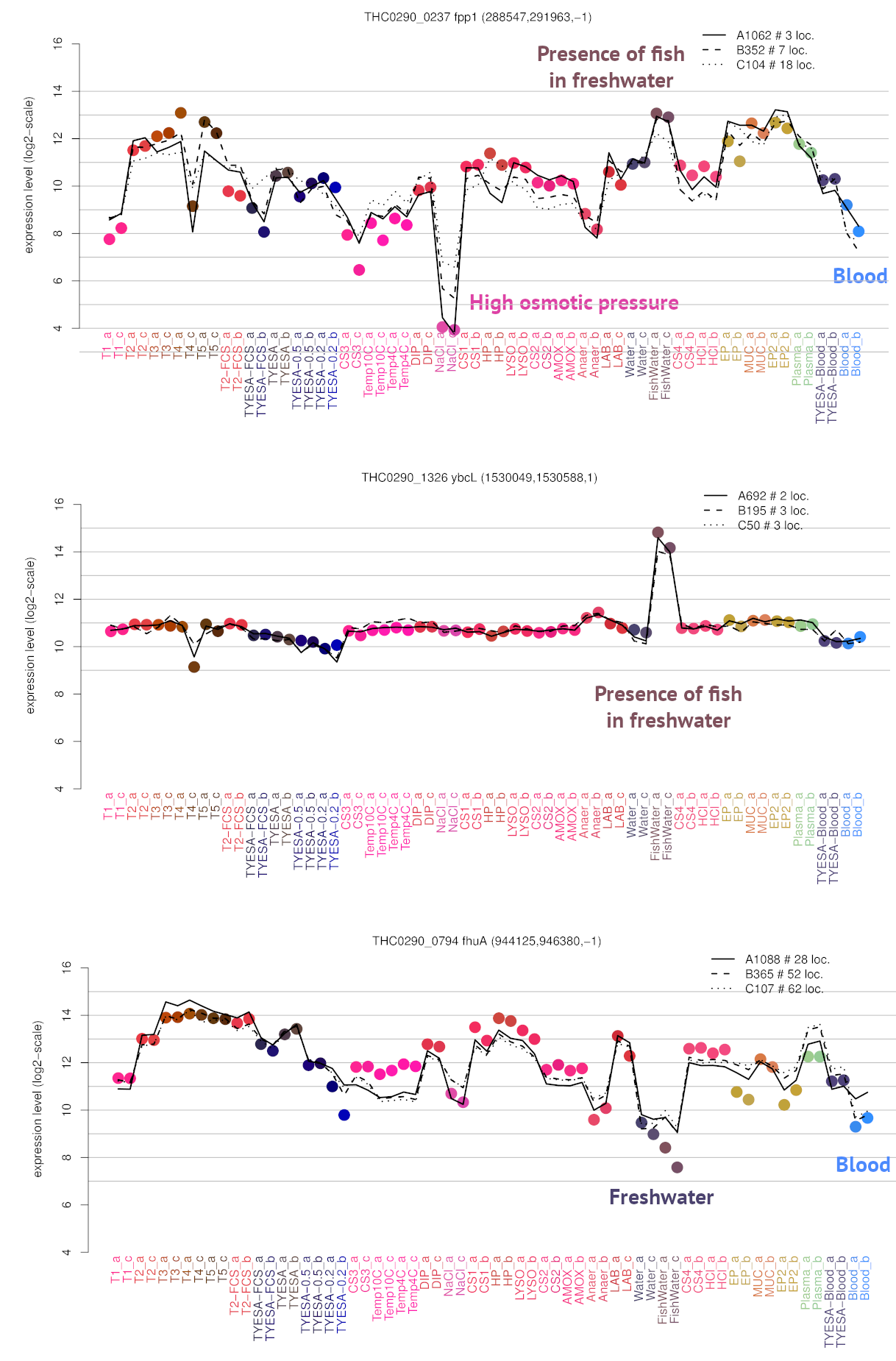


**Expression profile of *ybcL* gene from cluster C50 upregulated in freshwater only in the presence of fish.**

Genes related to fatty acids (FA) metabolism are differentially expressed in the presence of fish compounds. Gene cluster (C51) is highly up-regulated by the three conditions (mucus, plasma, freshwater in the presence of fish). It contains two operons, one encoding enzymes of the FA β-oxidation (THC0290_0982 and _0983, homologs of *B. subtilis* FadE and FadA, respectively), while the other encodes the electron transfer flavoprotein complex EtfAB that links β-oxidation with the respiratory system providing energy for ATP synthesis. The 2,4-dienoyl-CoA reductase encoded by THC0290_1892 (48% identity with FadH of *B. subtilis*), which is required for β-oxidation of unsaturated FA (22), is also highly up-regulated by fish mucus and plasma.


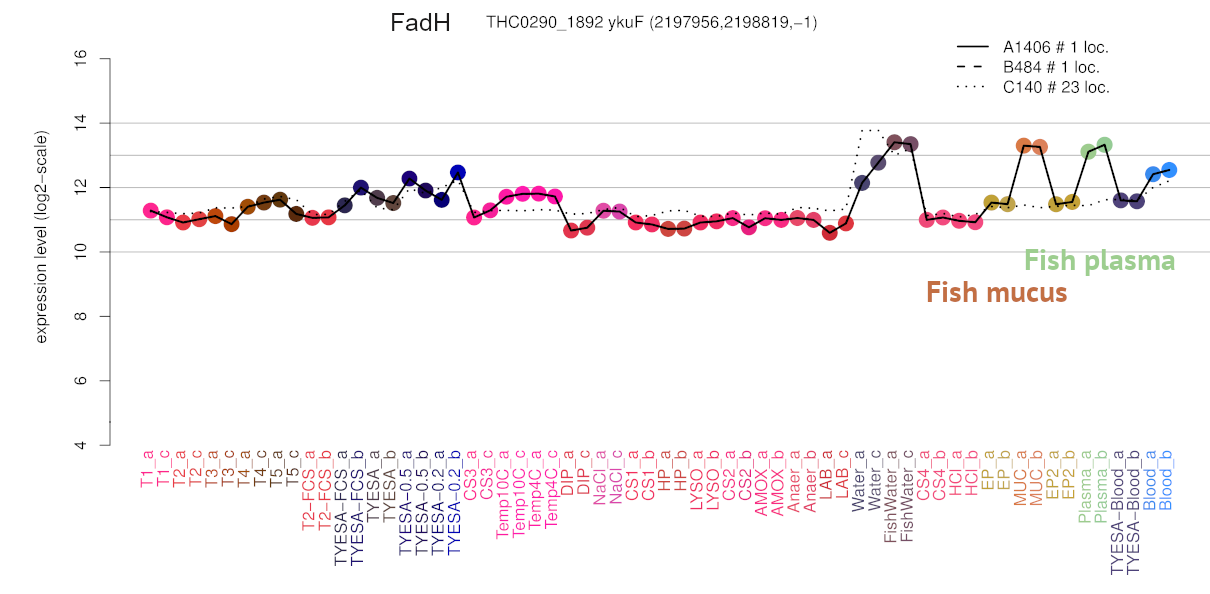


**Expression profile of *fadH* gene upregulated in the presence of fish compounds.**

Another aim of FA degradation can be to prevent damages induced by host-derived FA. Indeed, genes displaying the highest up-regulation under rainbow trout mucus exposure belong to cluster C48 which contains two oleate hydratases that catalyze the hydration of unsaturated FA and were shown to confer bacterial resistance to antimicrobial FA in some pathogenic bacteria (23, 24), a MFS type multidrug efflux system with homology to the EmrAB-TolC complex and the KdsA enzyme involved in (KDO)_2_-lipid A biosynthesis. A second MFS efflux system (C47) was induced by fish mucus. Tripartite efflux pumps are required during host infection and can help pathogenic bacteria to extrude host antimicrobial peptides and FA (25). Other genes up-regulated under fish mucus and plasma exposure have functions related to exopolysaccharides biosynthesis (clusters A580 and A581).

# D14. Life inside the host

In the host, *F. psychrophilum* has both to cope with diverse physicochemical constraints and to retrieve essential nutrients to survive and multiply.

Hydrogen peroxide exposure leads to the induction of typical oxidative stress response characterized by the overexpression of genes encoding the bi-functional peroxidase-catalase KatG, the thioredoxin-disulfide reductase TrxB1, three thioredoxins, the alkyl hydroperoxide reductase AhpC (THC0290_0979) and the components of the [Fe-S] clusters assembly machinery (cluster C66). The OxyR redox transcriptional activator (THC0290_0970), likely controlled by a thiol-disulfide switch, is induced by hydrogen peroxide. Two operons (clusters A1720 and A1721) encoding transcriptional factors of the MarR and ArsR families are also induced and transcribed with a nitroreductase and a glutaredoxin, respectively, indicating a role in detoxification of quinones and disulfides (26). Several highly up-regulated genes encode conserved proteins of unknown function (*e.g*. THC0290_0367, THC0290_0275, THC0290_0292) suggesting their involvement in oxidative stress resistance.

*F. psychrophilum* cells exposed to oxygen limitation adapt by triggering a strong up-regulation of the operon encoding *cbb3*-type cytochrome oxidase (*cbb3*-Cox). *cbb3*-Cox complexes have a high affinity for oxygen and allow bacterial growth under microaerobic conditions, including colonization of anoxic tissues by pathogenic bacteria (27). The *cbb3*-Cox encoding operon is co-expressed (cluster C52) with other hypoxia-induced genes including the oxygen-independent coproporphyrinogen III oxidase HemN required for heme biosynthesis in anaerobiosis and a monoheme *c*-type cytochrome of unknown function. Other hypoxia-induced genes include peroxiredoxin and thioredoxins as well as other enzymes of the heme biosynthesis pathway (clusters B46, A588 and B368) that could provide the porphyrins required for assembly of newly synthetized *cbb3*-Cox complexes.

The transcriptional response to high osmotic pressure is characterized by the induction of the operon encoding the high-affinity potassium transport system Kdp (cluster B539) which is related to osmoregulation in other bacteria, the gliding motility operon *remFG-sprCDBF*, the T9SS C-terminal signal peptidase PorU and several genes of unknown function. Interestingly, high osmotic pressure triggers a thiol-specific oxidative stress response in *F. psychrophilum*, characterized by the induction of KatG, three thioredoxins, three protein-methionine-S-oxide reductases and the thiol:disulfide oxidoreductases TlpA and TlpB. A *tlpB* mutant was reported to be deficient in gliding, secretion and virulence (28). Growth on blood also triggers transcriptional increase of those high osmolarity-induced genes (cluster C125).

Transcriptome profiles were analyzed under metal deprivation by growing *F. psychrophilum* into 2,2'-dipyridyl supplemented TYES broth to identify genes involved in this adaptation. Four TBDTs, which ensure substrate-specific transport across the outer membrane, are induced and could be involved in iron acquisition. Two of them are localized within a 19 kb-long locus containing at least 5 transcriptional units (cluster A1088), all found induced by iron scarcity (*i*.*e*. 2,2'-dipyridyl and fish plasma). Functions encoded at this locus are mainly unknown, however some proteins are related to iron acquisition in other bacteria, such as the iron(III) dicitrate-transport system FecA, a HmuY-like protein functioning as an hemophore in *P. gingivalis* (29) or two lipoproteins homologous to the iron-regulated protein IrpA shown to be necessary for growth under iron-deficient conditions in the cyanobacteria *Synechococcus* sp. (30). Among genes highly up-regulated in 2,2'-dipyridyl were those of clusters C48 and C52, encoding the two oleate hydratases related to FA detoxification, Cox and cytochromes c family proteins including the *cbb3*-Cox encoding operon related to hypoxia.


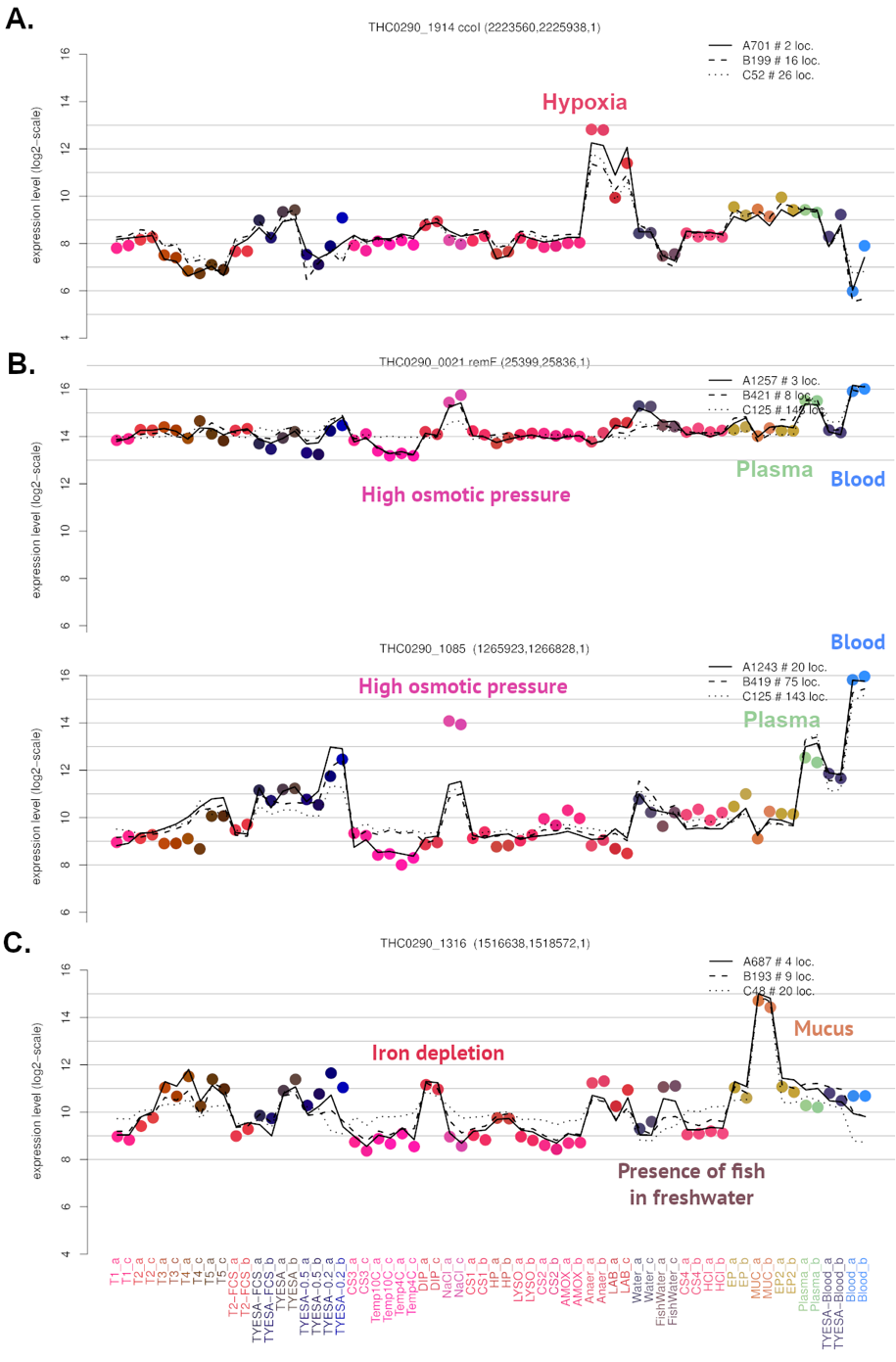


**Expression profiles of genes representative of the clusters up-regulated in within-host mimicking conditions.**

Quantile-normalized log2-expression values across 64 RNA samples of mRNAs belonging to gene clusters C52 (A), C125 (B) and C48 (C).

Exposure to fish plasma led to large amounts of deregulated genes (***SI* *Appendix* T4**) that include iron scarcity, hypoxia, osmotic and peroxide stress responses described above (*e.g*. gene clusters A1088, C52, B254, A1720 and A1721). Additional genes are induced such as two other tripartite efflux pumps (A1198, A1719), nine TBDTs that may play a role in blood-derived nutrients acquisition including iron sources (FhuA, HmuR, THC0290_1679) or cobalamin (THC0290_1776). Up-regulated transcription factors may contribute to the transcriptional reprogramming occurring under plasma exposure, such as the ferric uptake regulator Fur homolog (THC0290_0812), an uncharacterized σ^ECF^ sigma factor (THC0290_1773, cluster C139) and its cognate anti-sigma factor, or a PadR family transcription factor. Functions related to exopolysaccharides are induced: LptE is likely involved in translocation of newly synthetized LPS through the outer membrane, WbpM catalyzes the UDP-D-Qui2NAc O-antigen biosynthesis precursor, WcaJ catalyzes the UDP-glucose biosynthesis, first step in the assembly of colanic acid capsular exopolysaccharide in *E. coli*, and the oligosaccharide translocase WzxE (cluster A327) ensures the translocation of O-antigen subunits across the inner membrane allowing lipopolysaccharides assembly at the bacterial surface. Bacterial pathogens that spread into the bloodstream resist serum killing partly by modulating the synthesis of exopolysaccharides (LPS, capsule) in order to mask surface determinants recognized by the host’s complement. Several genes were commonly induced by growth on blood and plasma exposure (112 CDSs are induced in both Plasma.vs.Ctrl and Blood.vs.Ctrl pairwise comparisons). Numerous transcriptional units also carry unknown functions (*e.g*. cluster A1638).


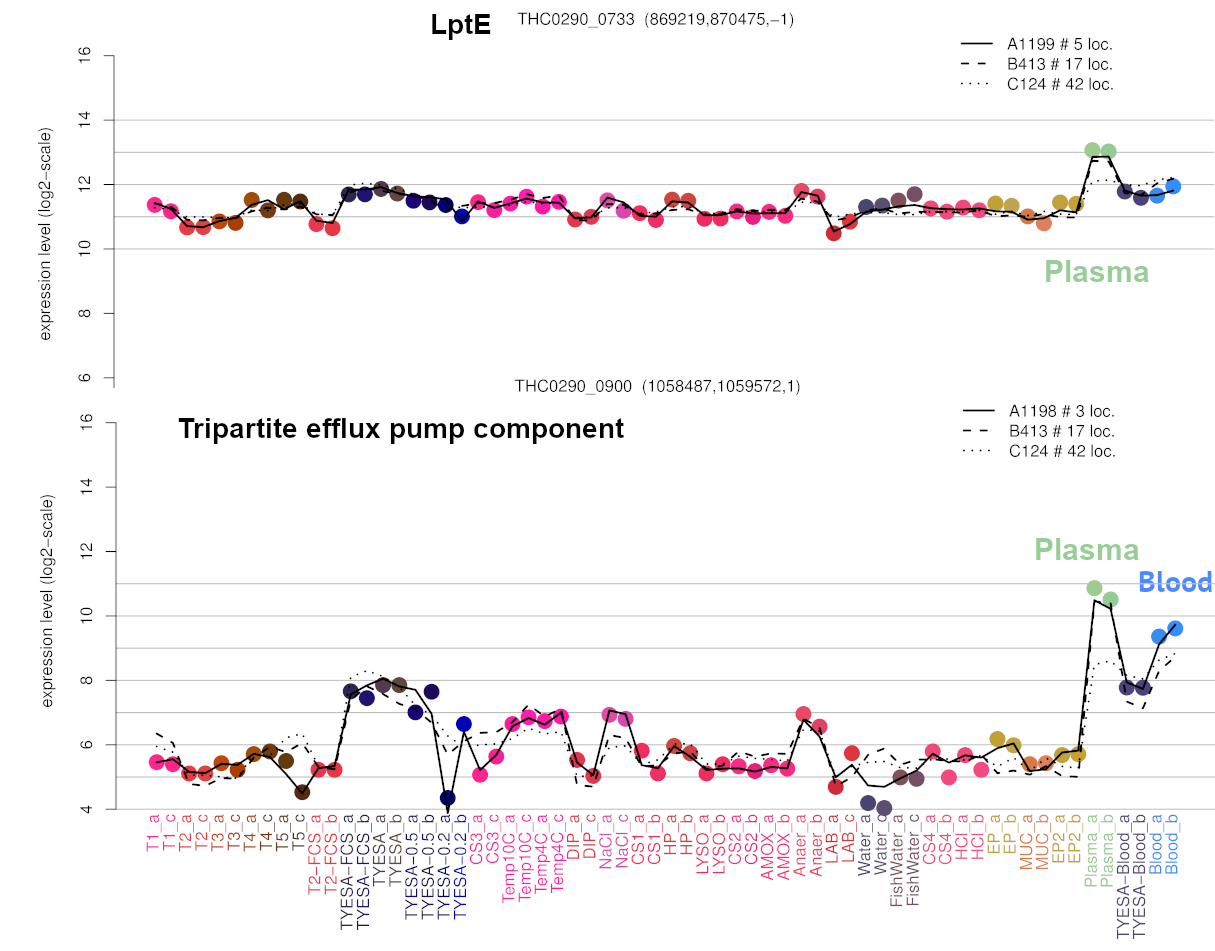


**Expression profiles of genes representative of the clusters up-regulated under fish plasma exposure.**

Quantile-normalized log2-expression values across 64 RNA samples of mRNAs belonging to gene clusters C124 (D).


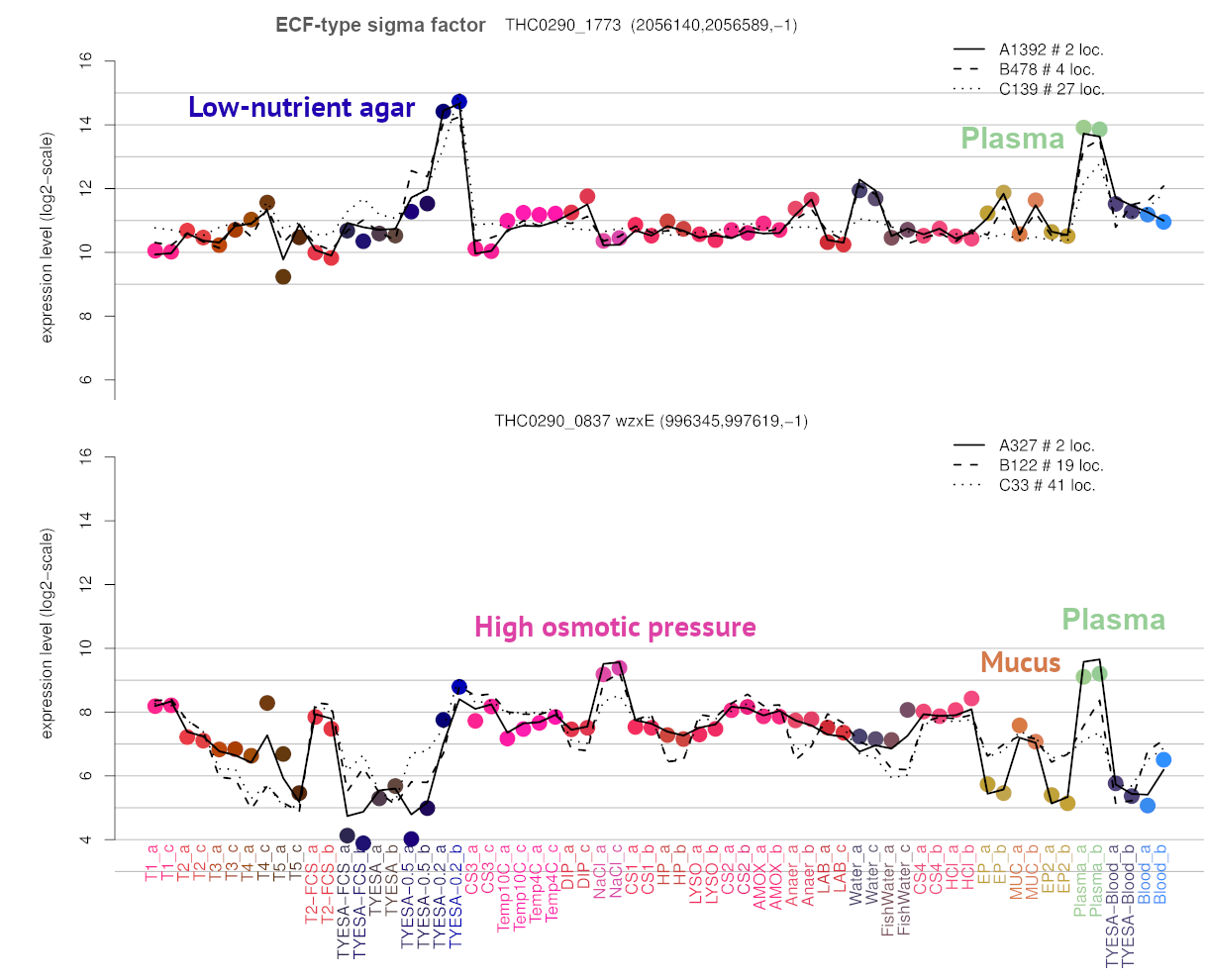


**Expression profiles of genes representative of the clusters up-regulated under fish plasma exposure.**

Quantile-normalized log2-expression values across 64 RNA samples of mRNAs belonging to gene clusters C139 and A327.

LaFrentz *et al* identified 20 proteins modulated under *in vivo* growth using intraperitoneal dialysis chambers implants in rainbow trout relative to *in vitro* growth using a comparative proteomic analysis of *F. psychrophilum* strain CSF-259-93 (31). We analyzed expression profiles of the corresponding genes in strain OSU THCO2-90 in our condition-dependent dataset and summarized the results as a table (see below). Half of the genes were found differentially expressed in host-mimicking conditions (*e.g*. 2,2-dipyridyl, fish plasma, hydrogen peroxide, osmotic stress, blood). Among them are the HmuY-like protein (FP1493), the gliding motility protein RemF (FP0012), the outer membrane OmpH (FP2098), the molecular chaperone HtpG (FP1509) and proteins of unknown function (FP1496, FP1486, FP2205) that are induced, while the outer membrane protein OmpA P60 is down-regulated.

**Differential expression analysis results for genes corresponding to the proteins identified by LaFrentz *et al* using proteomic analysis of *F. psychrophilum* cells cultured *in vivo* and in iron-limited media.**

| Locustag | |  | 2D-PAGE profiling^a^  from LaFrentz *et al* | | | Differential expression in pairwise comparisons  using the *in vitro* condition-dependent dataset^b^ | |
| --- | --- | --- | --- | --- | --- | --- | --- |
| JIP02/86 | THCO2-90 | Annotation | #spot | DIP | *in vivo* | Induction | Repression |
| FP1782 | THC0290_0926 | Ferritin FtnA | 1 | down | down | Anaer.vs.Ctrl, Water.vs.Ctrl, FishWater.vs.Ctrl, Blood.vs.Ctrl, Blood.vs.TYESA-0.2 | none |
| FP1493 | THC0290_1812 | Putative heme binding lipoprotein, HmuY-like family | 2 | up | up | DIP.vs.Ctrl, Trans.vs.Expo, Stat.vs.Expo | Blood.vs.Ctrl, TYESA-Blood.vs.Ctrl, Water.vs.Ctrl, FishWater.vs.Ctrl,  TYESA-0.5.vs.TYESA,  TYESA-0.2.vs.TYESA |
| FP0964 | THC0290_1283 | 3-oxoacyl-[acyl-carrier-protein]-reductase | 3 | up | up | none | Stat.vs.Expo |
| FP0467 | THC0290_0471 | Universal stress protein UspA | 4 | up | up | Trans.vs.Expo, Stat.vs.Expo,  Anaer.vs.Ctrl | Blood.vs.TYESA-0.2 |
| FP1496 | THC0290_1815 | Protein of unknown function precursor | 5 | up | up | DIP.vs.Ctrl, Plasma.vs.Ctrl, HP.vs.Ctrl, Trans.vs.Expo, Stat.vs.Expo | Anaer.vs.Ctrl, Water.vs.Ctrl, FishWater.vs.Ctrl, TYESA-Blood.vs.Ctrl, Blood.vs.Ctrl, Blood.vs.TYESA-0.2 |
| FP1970 | THC0290_0743 | Gliding motility protein GldN | 6 | up | up | none | none |
| FP2424 | THC0290_2349 | Probable lipoprotein precursor | 7 | n.d. | present | Plasma.vs.Ctrl, NaCl.vs.Ctrl | TYESA-0.5.vs.TYESA, TYESA-0.2.vs.TYESA |
| FP0012 | THC0290_0021 | Gliding motility protein RemF precursor | 8 |  | up | Plasma.vs.Ctrl, NaCl.vs.Ctrl, Blood.vs.Ctrl, Blood.vs.TYESA-0.2 |  |
| FP2098 | THC0290_0617 | Outer membrane protein OmpH | 9 |  | up | Blood.vs.Ctrl, Blood.vs.TYESA-0.2 | Stat.vs.Expo, FishWater.vs.Ctrl |
| FP1486 | THC0290_1805 | Protein of unknown function | 10 |  | up | DIP.vs.Ctrl, Plasma.vs.Ctrl, Trans.vs.Expo, Stat.vs.Expo | TYESA-0.5.vs.TYESA, TYESA-0.2.vs.TYESA, Anaer.vs.Ctrl, Water.vs.Ctrl, FishWater.vs.Ctrl, TYESA-Blood.vs.Ctrl, Blood.vs.Ctrl |
| FP0770 | THC0290_1448 | Probable acetyl-CoA acetyltransferase | 10 |  | up |  | Plasma.vs.Ctrl |
| FP1509 | THC0290_1828 | Chaperone protein HtpG | 11 |  | up | Blood.vs.TYESA-0.2 | Trans.vs.Expo, Stat.vs.Expo, TYESA-0.2.vs.TYESA, Water.vs.Ctrl, FishWater.vs.Ctrl |
| FP0110 | THC0290_0118 | Probable lipoprotein of unknown function | 12 |  | up |  |  |
| FP1765 | THC0290_0942 | ATPase with chaperone activity, ATP-binding subunit ClpB | 13 |  | up | Stat.vs.Expo, HP.vs.Ctrl, Anaer.vs.Ctrl | TYESA-0.2.vs.TYESA, Anaer.vs.Ctrl, Water.vs.Ctrl, FishWater.vs.Ctrl, Blood.vs.Ctrl |
| FP1888 | THC0290_0827 | Probable M1 family aminopeptidase precursor | 14 |  | up |  | Stat.vs.Expo, Anaer.vs.Ctrl |
| FP2411 | THC0290_2336 | Probable outer membrane protein, OmpA family | 15 |  | up |  | TYESA-0.5.vs.TYESA, TYESA-0.2.vs.TYESA, Water.vs.Ctrl, Blood.vs.Ctrl |
| FP2411 | THC0290_2336 | Probable outer membrane protein, OmpA family | 16 |  | up |  | TYESA-0.5.vs.TYESA, TYESA-0.2.vs.TYESA, Water.vs.Ctrl, Blood.vs.Ctrl |
| FP0321  FP1169 | THC0290_0334  THC0290_1930 | Probable outer membrane protein, OmpA family | 17 |  | up | FishWater.vs.Ctrl | Blood.vs.Ctrl |
| FP1167 | THC0290_1928 | Peptidyl-prolyl cis-trans isomerase precursor SurA | 18 |  | up |  | DIP.vs.Ctrl, Anaer.vs.Ctrl, Trans.vs.Expo, Stat.vs.Expo |
| FP2205 | THC0290_2159 | Protein of unknown function precursor | 19 |  | up | NaCl.vs.Ctrl, Blood.vs.Ctrl, Blood.vs.TYESA-0.2 | Stat.vs.Expo |
| FP0156 | THC0290_0161 | Outer membrane protein OmpA (P60) | 20 | present | n.d. |  | Plasma.vs.Ctrl, Blood.vs.Ctrl |

^a^ List of proteins retrieved from Table 1 of LaFrentz *et al* 2009 (31). 2D-PAGE and LC-MS/MS analyses were run on *F. psychrophilum* strain CSF-259-93 whole-cell lysate proteins extracted from cultures in TYES 2,2-dipyridyl (DIP) or in dialysis tubing chambers incubated into rainbow trout peritoneal cavity (*in vivo*). up: up-regulation, down: down-regulation compared to control condition (TYES), present: detected in the specified condition, n.d.: not detected. #spot: protein spot numbers from Table 1.

^b^ List of the pairwise comparisons for which the gene was found differentially expressed. Genes were considered as induced or repressed with a log2-FC > 1 (induction) or < -1 (repression) and FDR 5%. Full description of the pairwise comparisons is available in ***SI Appendix* T4** and in **Table S5**.

# D15. Expression of putative virulence factors across environmental conditions

Most proteins secreted through the T9SS are uncharacterized, a particular attention was paid to the changes in the expression of the corresponding genes (*i.e.* genes encoding proteins with a predicted Type A or Type B CTDs as reported by Barbier *et al*, 2020). Results of differential expression analyses were visualized as gene-condition interaction networks. Among the putative secreted adhesins, 17 tandem-arranged probable cell surface Leucine-rich repeat (LRR) proteins that belong to cluster C107 are all induced under fish plasma exposure and stationary phase of growth, while repressed in freshwater-living bacteria, by cold temperature or on blood agar. The first genes of the locus are also up-regulated in conditions of peroxide stress, serum supplementation and iron depletion. In other species, bacterial LRR proteins were shown to mediate host-pathogen interactions allowing adhesion to host cell surface receptors such as E-cadherin and immune receptors expressed by phagocytes (32). In *Leptospira* sp*.*, the abundance of genes encoding LRR proteins correlates with high virulence (33). By analyzing available genomic sequences of members of the family *Flavobacteriaceae*, we observed that the high number of LRR proteins in *F. psychrophilum* was a unique trait, other species encoding LRR-domain proteins such as *F. columnare* or *Capnotytophaga* *canimorsus* total fewer genes, none of them tandemly arranged.

**
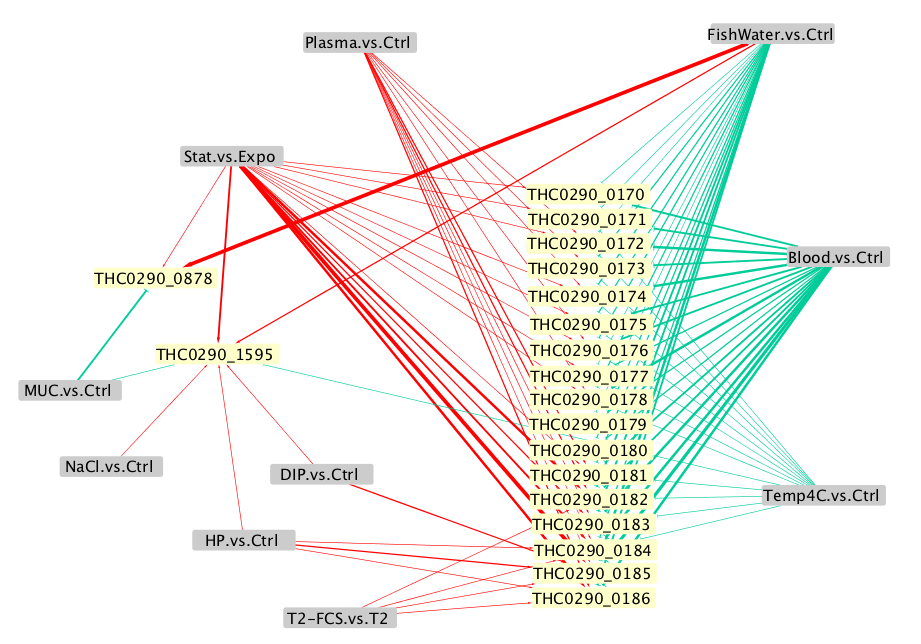
**

**Network reflecting transcriptional changes for putative Type A CTD adhesins.**

Network was built using Cytoscape v3.7.2 software (34). Nodes represent genes (grey) and biological effects (yellow). Red and green edges indicate differential expression (FDR 5% and log2-FC > 1 for induction, < -1 for repression, respectively) in the biological condition of interest. The width of edge increases with the absolute log2-fold-change value. The complete description of pairwise comparisons (test.vs.reference) is available in **Table S5**. THC0290_0878 (fibronectin type-III domain), THC0290_1595, and from THC0290_0186 to THC0290_0170, 17 tandemly arranged probable cell surface LRR family proteins.

Several secreted protein genes are also up-regulated under within-host mimicking conditions (*e.g.,* peroxide stress or iron deprivation), such as a probable ribonuclease (THC0290_1494) and an unknown function RCC1-repeat protein (THC0290_2385). Three adhesins, namely SprC, SprB and THC0290_0344 which contains Calx-beta repeats related to sodium-calcium exchangers, are up-regulated in response to high osmolarity. Some other predicted T9SS secreted protein encoding genes are up-regulated in freshwater and might have a role in host invasion such as putative adhesins and a secreted esterase/lipase/thioesterase family protein (THC0290_0977; see ***SI*** ***Appendix* D12**).

**
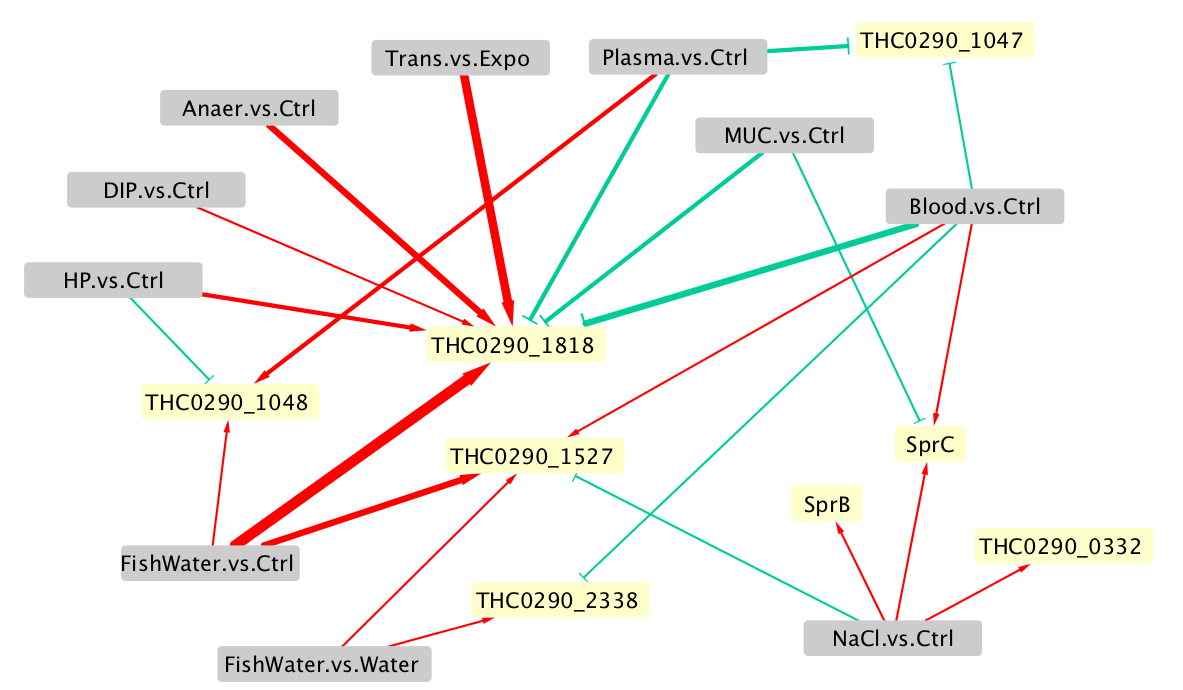
**

**Network reflecting transcriptional changes for putative Type B CTD adhesins.**

In strain OSU THCO2-90, 8 proteases are predicted to be secreted through the T9SS: the two tandem-arranged Fpp1 and Fpp2 metalloproteases, a collagenase (THC0290_0931) homologous to the Cog protein from *Cytophaga* sp. L43-1 (35, 36), two tandemly arranged M36 fungalysin family metalloproteases (THC0290_299 and THC0290_0300), a M1 family metalloprotease (THC0290_0086), a S8 subtilisin family serine endopeptidase (THC0290_0944) and a multidomain protein (THC0290_1343) carrying a metallopeptidase catalytic domain and several PKD repeat domains that may be involved in protein-protein or protein-carbohydrate interactions (19). Two additional lipoproteins with probable proteolytic activity were predicted to localize at the cell surface despite the lack of T9SS CTD: the M43 cytophagalysin family metalloprotease (THC0290_1090) and the streptopain family cysteine protease FcpB (THC0290_0491). In previous studies, inactivation of a single protease gene (*fpp1* or *fcpB*) in strain OSU THCO2-90 did not affect the LD50 determined by injection in rainbow trout, suggesting functional redundancy (37, 38). On the other hand, differences in the pool of functional proteases among *F. psychrophilum* strains isolated from different fish species suggest that some may act preferentially on specific fish host (17, 35). In an attempt to understand their contribution to the bacterial lifestyle, a network featuring links between extracellular proteases expression changes and environmental signals was built. Half of the proteases are modulated by many biological conditions. The expression of six genes increases during the transition from exponential to stationary phase of growth, indicating potential regulation by cell density or nutrients starvation. Three of them, the collagenase, Fpp1 and THC0290_1343, are also over-expressed under iron depletion. Fpp1 and the probable fungalysin metalloprotease THC0290_0300 are up-regulated in freshwater-living cells in the presence of fish, but are strongly repressed in cells grown under *in vivo* mimicking conditions (oxygen limitation, presence of blood, high osmotic pressure). In contrast, the expression of the collagenase and the probable M43 cytophagalysin family metalloprotease were up-regulated in cells grown on blood agar and may be required for optimal growth under these conditions.

**
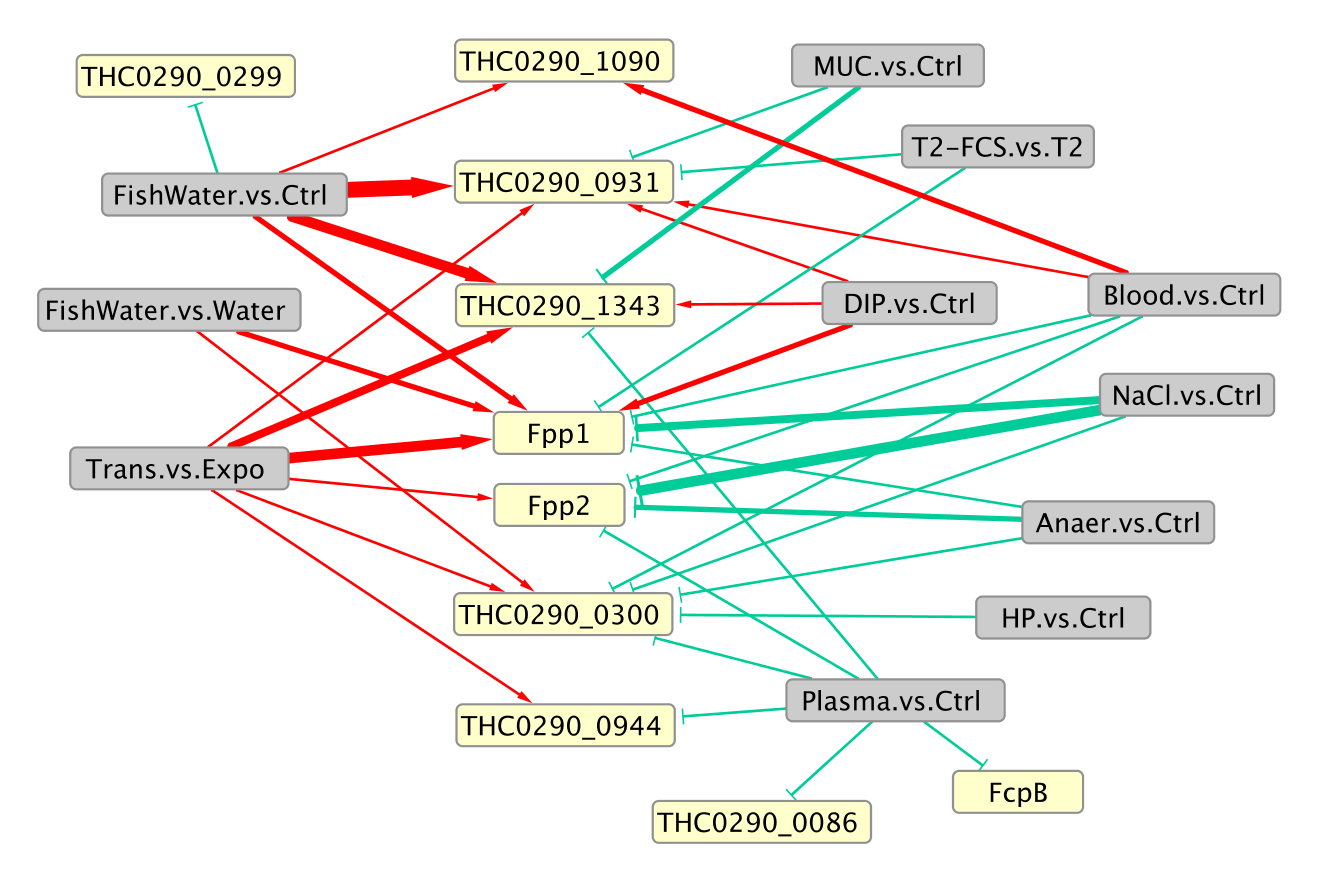
**

**Network featuring secreted or surface exposed proteases encoding genes**.

THC0290_0086 Probable M1 family metalloprotease (T9SS type A CTD)

THC0290_0237 *fpp1* Psychrophilic metalloprotease Fpp1 (T9SS type A CTD)

THC0290_0238 *fpp2* Psychrophilic metalloprotease Fpp2 (T9SS type A CTD)

THC0290_0299 Probable M36 fungalysin family metalloprotease (T9SS type A CTD)

THC0290_0300 Probable M36 fungalysin family metalloprotease (T9SS type A CTD)

THC0290_0491 *fcpB* Streptopain family cysteine protease (predicted extracellular lipoprotein)

THC0290_0931 Collagenase precursor (T9SS type A CTD)

THC0290_0944 Probable S8 subtilisin family serine endopeptidase (T9SS type A CTD)

THC0290_1090 Predicted surface exposed lipoprotein, M43 cytophagalysin family metalloprotease

THC0290_1343 Protein of unknown function, PKD and metallopeptidase domains (T9SS type A CTD)

We showed that the low expression of Fpp2-Fpp1 and THC0290_0300 mRNAs measured by transcriptomics under high osmotic pressure is correlated to a strong reduction of exoproteolytic activity measured by *in vitro* assays using azocasein as the substrate. Supply of fetal calf serum in TYES broth also results in down-regulation of *fpp1* and collagenase genes, and concordantly, a weak exoproteolytic activity was measured in spent culture medium, indicating that other signals than osmolarity can drive their repression.

**A.**


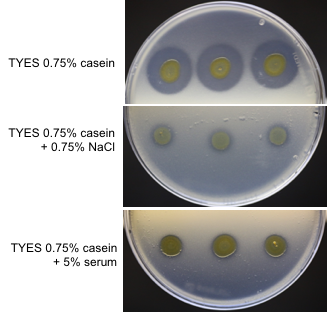


**B.**


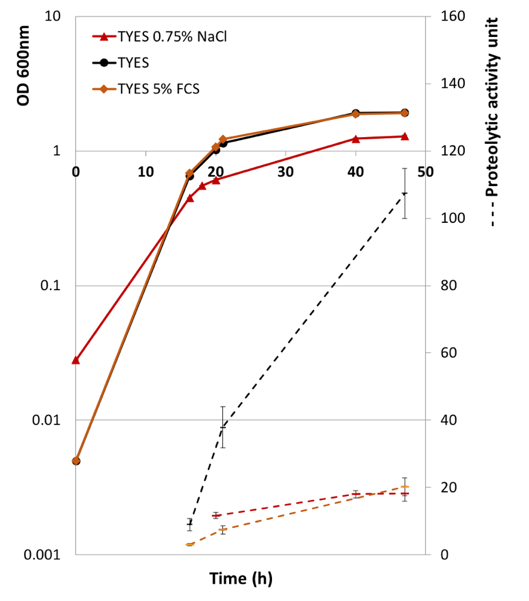


**Exoproteolytic activity of *F. psychrophilum* OSU THCO2-90 is reduced in cells exposed to high osmotic pressure or the presence of serum.**

**A.** Ten µL of stationary phase cultures performed in triplicate were spotted on TYES agar supplemented with casein (0.75%) and either NaCl (0.75%) or fetal calf serum (FCS, 5%). Proteolytic activity was visualized as clearing zones around the bacterial growth after incubation at 18°C for 6 days. **B.** Proteolytic activity measured using azocasein as substrate. Cells were grown in TYES broth supplemented with either NaCl (0.75%) or FCS (5%) at 18°C with shaking. Proteolytic activity was measured in cell-free supernatants as previously described (19). All cultures were performed in triplicate and an average of the three experiments was used as the measure of proteolytic activity over the growth curve. One unit of proteolytic activity was defined as an increase in absorbance of 0.001 under the conditions of the assay. Values and error bars are means and standard deviations, respectively. Solid lines: OD600 indicating growth. Dashed lines: proteolytic activity units. Color code: black (no supplementation), red (NaCl), orange (FCS).

SI Appendix, Materials and Methods

# M1. Bacterial strains and growth conditions

*F. psychrophilum* OSU THCO2-90 was routinely grown aerobically in tryptone yeast extract salts (TYES) broth [0.4 % (w/v) tryptone, 0.04 % yeast extract, 0.05 % (w/v) MgSO_4_ 7H_2_O, 0.02 % (w/v) CaCl_2_ 2H_2_O, 0.05 % (w/v) D-glucose, pH 7.2] or on TYES agar. The medium was supplemented with 5% fetal calf serum (FCS) for optimal growth when specified. Growth in liquid culture was carried out at 200 rpm and 18°C and monitored by measuring OD_600nm_ using a Biochrom WPA CO8000 spectrophotometer. Two distinct sets of growth conditions were used for RNA-sequencing and microarrays experiments. The first set corresponds to a pool of 18 total RNA samples, each extracted in a different biological condition (thus referred to as 18-condition RNA pool), which was used for transcriptome and TSS mapping by RNA-seq. The second set is composed 64 RNA samples accounting for 32 biological conditions, and served to analyze the expression profiles across conditions using high-density microarrays (***SI Appendix* T3**). A full description of the two sets of biological conditions is available in **Table S1**.

*E. coli* strains were grown at 37°C in Luria Bertani broth or with 15 g of agar per liter for solid medium. For selective growth of *E. coli* strains carrying the pCP*Gm*^r^- and pYT313-derivative plasmids, transformants were selected with 100 µg mL^-1^ of ampicillin. Cultures of *E. coli* MFD*pir* were supplemented with 0.3 mM diaminopimelic acid (Sigma-Aldrich Co.). Selection of *F. psychrophilum* transconjugants was carried out with 50 µg mL^-1^ of gentamycin and 10 µg mL^-1^ of erythromycin for pCP*Gm*^r^- and pYT313-derivative plasmids respectively (18, 19).

# M2. Construction of transcriptional reporter plasmids and promoter activity monitoring

Oligonucleotides are listed in ***SI Appendix* T2**. A transcriptional reporter plasmid and derivatives carrying different promoter fragments were constructed using as backbone pCP*Gm*^r^-*gldD*, a pCP23-derivative vector carrying a gentamycin resistance marker (39). The promoter-less vector named pCP*Gm*^r^-P_less_-mCh was designed to carry, in that order: an intrinsic terminator (DNA region downstream of THC0290_0151), a multi-cloning site (NheI, NotI, AflII, SpeI, XhoI), a *F. psychrophilum* codon-adapted mCherry DNA coding sequence and the intrinsic terminator sequence of *gldD*.

**
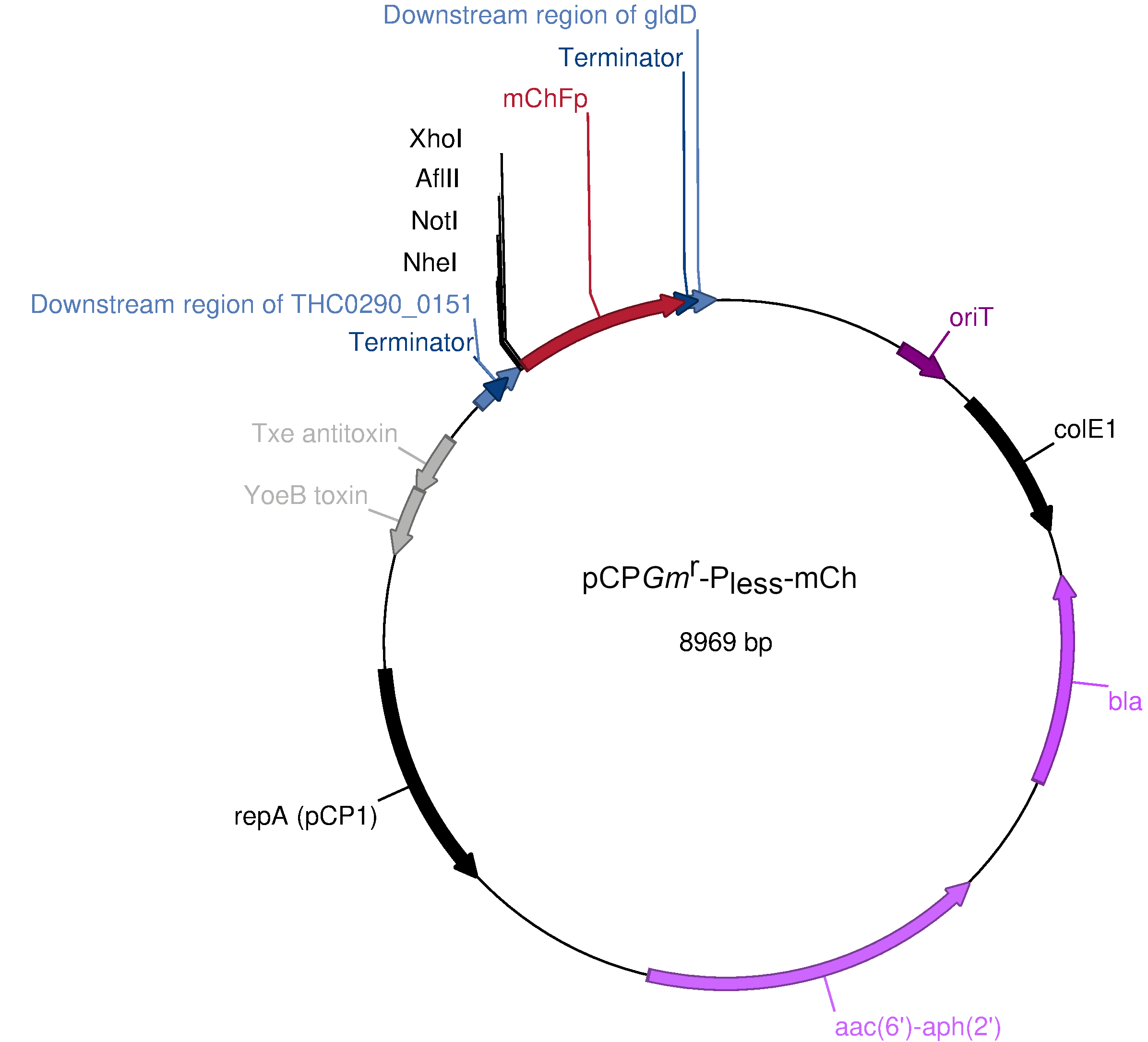
**

**Graphical map of the transcriptional fusion pCP*Gm*^r^-P_less_-mCh plasmid.**

Codon adaptation was designed using OPTIMIZER (40), the codon-adapted mCherry gene was synthetized and cloned into pUC57 (Genewiz^®^, Sigma-Aldrich).

**DNA sequence of mCherry gene codon-adapted for expression in *F. psychrophilum* (Genbank ID: MW401799)**

>mChFp

ATGGTAAGCAAAGGAGAAGAAGATAACATGGCCATTATTAAAGAATTTATGAGATTTAAAGTACACATGGAAGGATCTGTAAACGGACACGAATTTGAAATTGAAGGAGAAGGAGAAGGAAGACCATACGAAGGAACCCAAACCGCCAAATTAAAAGTAACCAAAGGTGGACCATTACCATTTGCCTGGGATATTTTATCTCCTCAATTTATGTACGGATCTAAAGCCTACGTAAAACACCCAGCCGATATTCCAGATTACTTGAAATTATCTTTTCCAGAAGGATTTAAATGGGAAAGAGTAATGAACTTTGAAGATGGAGGAGTAGTAACCGTAACCCAAGATTCTTCTTTACAAGATGGAGAATTTATTTACAAAGTAAAATTAAGAGGAACCAACTTTCCATCTGATGGACCAGTAATGCAAAAAAAAACCATGGGATGGGAAGCCTCTTCTGAAAGAATGTACCCAGAAGATGGAGCCTTAAAAGGAGAAATTAAACAAAGATTAAAATTAAAAGATGGAGGACACTACGATGCTGAAGTAAAAACCACCTACAAAGCCAAAAAACCAGTACAATTACCAGGAGCCTACAACGTAAACATTAAATTGGATATTACCTCTCACAACGAAGATTACACCATTGTAGAACAATACGAAAGAGCCGAAGGAAGACACTCTACCGGAGGAATGGATGAATTATACAAATAG

pCP*Gm*^r^-P*_less_*-mCh was constructed by Gibson method. Three DNA fragments were amplified by PCR using Phusion High-Fidelity DNA polymerase (Thermo Fisher) as follows: the vector fragment using pCP*Gm*^r^-*gldD* DNA as matrix and primers TRO454/TRO455, the upstream terminator fragment using primers TRO456/TRO457, the mCherry gene using primers TRO458/TRO459. The resulting PCR products were assembled using the NEBuilder^®^ HiFi DNA Assembly Master Mix (New England Biolabs). pCP*Gm*^r^-P*_less_*-mCh derivative carrying *remF* promoter was constructed by Gibson assembly of two DNA fragments: the vector fragment was amplified using primers TRO460/TRO461 with pCP*Gm*^r^-P*_less_*-mCh as matrix and the *remF* promoter region (409 bps) using primers TRO470/TRO609, resulting in pCP*Gm*^r^-P*_remF_*-mCh. Point-mutations were introduced to pCP*Gm*^r^-P*_remF_*-mCh by amplification of the entire plasmid template using primers with the desired mutations as follows: *remF-sm4** and *remF-sm5** were generated using TRO609/TRO610 and TRO615/TRO616, respectively, and pCP*Gm*^r^-P*_remF_*-mCh DNA as matrix; *remF-sm4*sm5** was then introduced in pCP*Gm*^r^-P*_remF-sm5*_*-mCh using TRO609/TRO610. Engineered plasmids were constructed in *E. coli* DH5-alpha Z1, verified by PCR and DNA sequencing, transferred to *E. coli* MFD*pir* and introduced into *F. psychrophilum* OSU THCO2-90 by conjugation as previously described (18).

*F. psychrophilum* strains carrying the pCP*Gm*^r^-P*_less_*-mCh and P*_remF_* derivatives were grown in TYES broth supplemented with 20 µg mL^-1^ gentamycin. Expression of the *mCherry* gene was monitored in exponential growth phase using whole-cell fluorescence with a Tecan Microplate Reader (Infinite 200 PRO). Excitation and emission wavelengths were set at 535 nm and 610 nm, respectively. Promoter activity (in arbitrary units) was estimated by dividing fluorescence intensity by OD_600._

Proteolytic activity assays were performed as follows: *F. psychrophilum* strains were grown in 25 mL TYES broth at 18°C and 200 rpm for 2 days and sampled (2 mL) at OD_600_ of 0.5, 1.2 and 2.0. Samples were centrifuged, the supernatants filtered using 0.22 µm Millipore filters and stored at 4°C for 24 h. Proteolytic activity was quantified on azocasein substrate as previously described (19).

# M3. Construction of the *rfp18* deletion mutant

The pYT313-*rfp18* suicide vector was constructed using pYT313-derivative plasmid (41) by Gibson method using oligonucleotides listed in ***SI* *Appendix* T2**. Three DNA fragments were amplified by PCR as follows: the plasmid fragment using pYT313 DNA as matrix and primers TRO186/TRO187, the 1,968-bp fragment upstream and the 2,053-bp fragment downstream of *rfp18* with chromosomal DNA and primers TRO721/TRO722 and TRO723/TRO724, respectively. The chromosomal structure of clones selected after double crossing over was analyzed by PCR using the following primers: TRO566/TRO726 (right-hand junction), TRO565/TRO727 (left-hand junction), TRO565/TRO566 (internal fragment of *rfp18*) as well as TRO726/TRO727 (fragment encompassing the *rfp18* gene). The resulting pYT-*rfp18* plasmid was introduced by conjugation in strain OSU THCO2-90 and colonies carrying chromosomal integration of the plasmid were selected using erythromycin resistance. The second recombination event (plasmid excision) was obtained by plating a stationary phase culture inoculated with a single colony in TYES broth without antibiotics on TYES supplemented with 5% sucrose. The chromosomal structure of erythromycin-sensitive clones was determined by PCR in order to identify the *rfp18* deletion mutant.

# M4. RNA extraction, libraries preparation and RNA-sequencing

Total RNA extractions were performed using the hot phenol method as previously described (18). 50 µg of RNA extracts were treated using TurboDNase I (Ambion) to remove residual genomic DNA and then purified using the RNA Clean-Up and Concentration Kit (Norgen Biotek). RNA concentration of the DNase-treated samples was measured using a NanoDrop 1000 Spectrophotometer (NanoDrop Technologies, Inc.) and RNA quality was assessed by capillary electrophoresis using RNA Nano chips with a Bioanalyzer Agilent 2100 (Agilent Technologies, Palo Alto, USA). An equimolar 18-condition RNA pool was obtained by mixing each of the extracts in equal quantity.

Preparation of the library for 5’-end RNA-Seq started with 35 µg of the RNA pool. A first ligation was performed over-night at 16°C with a RNA adapter (5’-GCAUAGGGGUAAA-3’) using 4 units/µg of T4 RNA ligase (New England Biolabs, NEB). After phenol-chloroform RNA extraction and removal of non-ligated RNA adapters using a Micro Bio-spin column P-30 (Biorad), 30 µg of purified RNA sample was treated by 50 units of Tobacco Alkaline Phosphatase (TAP, Epicentre) to transform 5’-triphosphate groups carried by primary transcripts into 5’-monophosphate groups. The reaction was performed at 37°C for 2h, and followed by phenol-chloroform purification. The TAP-treated RNA sample was then used to prepare the strand-specific 5’-end RNA-Seq library. First, a second RNA adapter (5’-GUUCAGAGUUCUACAGUCCGACGAUC-3’) was ligated to the 5’ end of the TAP-treated RNAs in a 10 µL reaction containing 40 ng RNA, 1 µM adapter, 10 units RNA ligase 1 (NEB), 1x RNA ligase 1 reaction buffer and 40 units RNase inhibitor. Incubation was at 20 °C for 6 h followed by purification on RNeasy columns (Qiagen). RNA was eluted in 15 µL water and subsequently fragmented using fragmentation buffer (Ambion) following the manufacturer’s protocol, then purified again. Phosphatase treatment was performed using 5 units Antarctic phosphatase (NEB) and incubation for 30 min at 37 °C and 5 min at 65 °C. RNA was purified by RNeasy purification, eluted in 18 µL water and then vacuum concentrated to 5 µL. The Illumina small RNA library preparation kit was used for 3’ adapter ligation, subsequent reverse transcription and PCR following the kit protocol except that half of the reaction volumes were used. Size selection was performed on 4% low melting agarose gels to remove adapter dimers.

For global RNA-Seq, 20 µg of the 18-condition RNA pool were processed using MICROBExpress (Ambion) according to the manufacturer’s instructions to remove ribosomal RNAs. Efficient rRNA depletion was confirmed using Bioanalyzer 2100. Strand specific transcriptome RNA-Seq library was performed following a similar procedure as described above for 5’-end RNA-Seq, except for the ligation of a 5’ adapter prior to RNA fragmentation. Here, RNA was first fragmented and subjected to 3’ and 5’ adapter ligation (in this order), then reverse transcription and PCR using the Illumina small RNA library preparation kit following the kit protocol.

# M5. Read mapping

Adapters were removed using cutadapt (42) and low-quality base pairs in 3’-end were trimmed using sickle (version 1.330, options “-x -n” with default Phred quality cut-off of 20). Reads were aligned to the *F. psychrophilum* OSU THCO2-90 complete genome sequence (ENA accession number LT670843) using bowtie2 (version 2.2.4) (43) with options “--very-sensitive --score-min L,-0.2,-0.2” where the modification of the minimal score discarded some low quality matches. Multiple mapped reads of the TSS-library were discarded from the dataset prior to transformation of both mapped dataset results in BAM format using SAMTools (version 1.1) (44).

# M6. Determination of putative TSSs

Identification of putative TSSs was based on the exact starting positions of uniquely mapped reads of the 5’-end RNA-Seq library. Positions on the genome with at least 3 starting reads were selected if this number of reads was 100x above the mean coverage of their upstream (from -220 to -20 bp) and downstream (from 20 to 220 bp) regions. If less than 8 bp apart, these positions were aggregated, and the position with highest coverage was used as the consensus. Putative TSSs identified with these criteria were masked and the procedure was repeated in order to identify additional putative TSSs.

# M7. Delineation of newly defined transcribed regions

As a complement to the genome annotation (45), RNA predictions were also retrieved from the RFAM database (9). This RFAM list contained 9 putative RNAs related to well-known functional families (RNase P, signal recognition particle RNA, transfer-messenger RNA, 6S-RNA, tryptophan peptide leader RNA, cobalamin, 2 TPP and SAM-II long loop riboswitches). The other predictions corresponded to 128 occurrences of the Flavo-1 RNA motif, a conserved RNA structure of unknown function identified *in silico* (10). In absence of functional evidence, these 128 features were not considered as *bone fide* annotated RNA features during the transcribed regions detection procedure (see below).

Genomic segments transcribed outside the previously released genome annotation and RFAM predictions were delineated based on expression-level reconstructed from the distribution of 5’-ends of global RNA-Seq reads with the state-space model of Parseq (46). The cut-off used for this purpose was expression-level above 0.01 read 5’-end/bp with probability 0.95. Regions were extended in both directions based on a relaxed probability of 0.50 to avoid segmentation artifacts near the cut-off value. Since the regions were defined on the sole basis of the distribution of 5’-ends, their 3’-ends were shifted downstream by 40 bp (approximately the average read length after trimming).

These transcribed regions were further decomposed into more homogeneous segments according to (i) genome annotation (45), (ii) RFAM predictions and intrinsic terminators identified *in silico* by Petrin (47) and (iii) probable TSSs identified by 5’ RNA-Seq and confirmed by prediction of a sigma-factor binding site (this study). Coincidence of 3’-ends of 3’ transcribed regions with intrinsic terminators was defined as the condition where the 3’-end of a predicted intrinsic terminator was between -50 bp and +10 bp of the 3’-end of the TR.

Expert curation and classification of the resulting segments relied on visual inspection of 2190 segments along the chromosome. A total of 608 segments considered as putative artefacts were discarded. After further exclusion of 71 segments surrounded by repeated regions, the remaining segments (1511) were considered as newly defined transcribed regions (hereafter named TRs) and classified into different categories according to their transcriptional context (presence of adjacent CDS, intrinsic terminator, putative TSS detected by 5’-end RNA-seq). These categories are described in ***SI* *Appendix* D1**.

Presence of putative short CDSs in TRs was examined using the SHOW software based on a probability cut-off of 0.5 and a minimal length of 15 aa (48). TRs overlapping annotated CDSs on the opposite strand were considered as antisense RNAs if the overlap was ≥ 50 bp or represented ≥ 50% of the region or the CDS.

# M8. High-density microarray design and strand-specific hybridization procedure

Microarray design was performed using Oligowiz (version 2.0) (49). Options “-minlh 20 -length 50 -lmin 40 -lmax 60 –optTM 71” were used to select candidate probes with target TM of 71°C, target length of 50 bp, and limited self-annealing and cross-hybridization potential. The final selection of probes made with the graphical interface applied a minimal start-to-start distance between probes of 40 bp and the “prokaryotic random priming score” (*i.e.* no preferential position within regions). The target number of probes per annotated gene, RFAM predictions (excepted Flavo-1), or newly delineated transcribed segment was set to 15. For each TSS we also defined two flanking regions (150 bp starting at 5 bp upstream and downstream the TSS). The target number of probes for TSS-flanking and Flavo-1 regions was set to 3. Probes were duplicated when these target number of probes could not be reached due to short length or sequence properties of the regions.

Strand specific synthesis of Cy-3-labeled DNA from the DNase-treated RNA samples was performed using Fairplay III Microarray labelling kit (Agilent technologies) with random priming and 50 µg/mL actinomycin D (VWR) (50). One-color hybridization was performed on SurePrint G3 Custom GE 8x60K microarrays (Agilent technologies) as recommended by suppliers. The “gProcessedSignal” produced by Agilent Feature Extraction software served as probe-level raw expression values.

An aggregated raw expression value was computed for each region of the structural annotation as the median of probe-level values. CDS expression values were quantile-normalized between all experiments (64 hybridizations data) using the R package ‘limma’ (51) and this transformation was propagated to all other types of regions. Pairwise differential expression analyses were performed on relevant reference/test conditions using ‘limma’. P-values were adjusted for multiple testing using the Benjamini-Hochberg method to control the false discovery rate. Principal Component Analysis was carried out using the R package ‘prcomp’ after centering (but not scaling) the log2 expression levels for each gene. Hierarchical clustering of genes used average-link method implemented in the R function ‘hclust’ and Pearson distance (1-r).

# M9. Sigma factors binding sites discovery

Promoter activity was measured by probes in TSS-flanking regions. A promoter tree summarizing similarity between promoter activities across samples was built by hierarchical clustering and average link based on estimated pairwise correlation coefficients, considering data censoring by upstream signal and signal saturation (2). Promoter sequences (-50bp, +10bp around the TSS) were analyzed together with the hierarchical clustering promoter tree using TreeMM algorithm (2). Among putative TSSs identified by 5’-end RNA-Seq, 16 were discarded based on all-against-all BLASTN comparison (default alignment parameters, e-value cut-off 0.001) to remove repetitions that could mislead the search for shared motifs. A unique representative promoter sequence was selected for each sequence repeated 3 or more times. After analyzing the results of pilot runs the set-up was slightly modified compared to (2) to improve convergence. Namely, the number of motifs was fixed to 8; the allowed intervals for the widths of the first and second boxes were (8,25) and (1,6), respectively; the MCMC algorithm was run for a total of 200000 sweeps with the first 20000 without the second box and allowing heuristic non-reversible component-split move to replenish empty components for 40000 sweeps; the length of the burn-in period was set to 50000 sweeps and 20 random seeds were tested (before selecting the best in terms of likelihood).

# M10. Computational analysis of newly defined transcribed regions

To characterize their phylogenetic conservation, DNA sequences of new TRs identified in strain OSU THCO2-90 were compared to a database of 64 genomes using BLASTN (version 2.10.0+, (52)) with a cut-off evalue set to 0.01, tuned parameters to improve detection of short and distant homologs (gap open: -2, gap extend: -1, mismatch: -1, match: 1, word_size: 4), and requiring an alignment over 50% of the query.

RNA secondary structures were predicted with RNAfold (ViennaRNA package, version 2.3.4, parameters -p -d2 –noLP) and visualized with the forna web interface (13). Statistical support for significant RNA folding was summarized in terms of a z-score comparing the optimal minimum free energy (MFE) returned by RNAfold to the mean and standard deviation estimated on 100 randomly shuffled versions of the original TR sequence (53).

Target mRNA-sRNA interactions were predicted using CopraRNA run via its web interface with default parameters (16). Sequences of 9 selected putative small RNAs TRs homologs (identified by the aforementioned BLASTN search) were used as input. When homologs could not be used due to too low sequence divergence, putative targets were ranked based on the sole IntaRNA score which predicts interactions in single organisms (54). Searches were run on strain JIP02/86 due to genome availability on the Freiburg RNA tools.

# M11. Real Time quantitative PCR

Total 3 µg RNA was treated with DNase I (Qiagen) to remove genomic DNA and then purified using the RNA Clean-Up and Concentration Kit (Norgen Biotek) following manufacturer’s instruction. Reverse transcription reaction was carried out on 500 ng RNA in thermal cycler (Eppendorf) using iScript Advanced cDNA Synthesis Kit for RT-qPCR (BIO-RAD) following manufacturer’s instruction. cDNA was diluted 1:30 in water and mixed with iTaq Universal SYBR green supermix (BIO-RAD) and forward and reverse primers with final concentration at 312.5 nM each. qPCR was performed using CFX system (BIO-RAD) with initial denaturation at 95°C for 3 mins followed by 40 cycles of amplification as following: denaturation at 95°C for 5 s, annealing and extension at 60°C for 30 s. Relative expression values are from five independent cultures, measured in technical triplicates. Two reference genes (*gmd* and *parE*) were selected based on stability of their expression level in the microarray condition-dependent dataset using NormFinder software (55).

**Assessment of mRNA stability of 10 candidate reference genes using NormFinder.**

| Locustag | Gene | Stability |
| --- | --- | --- |
| THC0290_1412 | *recJ* | 0.2 |
| THC0290_1069 | *gmd* | 0.21 |
| THC0290_0916 | *rpsA* | 0.25 |
| THC0290_1081 | *dapD* | 0.25 |
| THC0290_1933 | *prfC* | 0.25 |
| THC0290_0440 | *frr* | 0.27 |
| THC0290_1568 | *parE* | 0.28 |
| THC0290_1417 | *cysK* | 0.29 |
| THC0290_0741 | *gldL* | 0.31 |
| THC0290_1227 | *susC* | 0.37 |

Stability estimation was obtained based on log2-expression values of 64 microarrays data.

Genes are ranked according to their expression stability.

For each biological replicate, mean Ct values were calculated based on technical triplicate reactions. The stability of Ct values was confirmed for the two reference genes and values of others genes normalized using the geomean of *gmd* and *parE* Ct values. Relative quantification of mRNA was expressed as 2^–∆∆Ct^ using wild-type at OD600 0.5 as reference sample. Statistical differences of mRNA levels between wild-type and Δ*rfp18* through three phases of growth were analyzed using two-way ANOVA with GraphPad version 8.2.0. Bonferroni adjusted pvalues < 0.05 were considered significant.

# M12. Reverse Transcription PCR

Total RNA was extracted from colonies of *F. columnare* strain ATCC 49512, treated with DNase I (Qiagen) and then purified using the RNA Clean-Up and Concentration Kit (Norgen Biotek) following manufacturer’s instruction. cDNA synthesis was carried out on 400 ng of DNase-treated RNA using iScript Advanced cDNA Synthesis Kit. No RT negative control was run in same condition except that reverse transcriptase was omitted. Expression of Rfp18 homolog was detected by PCR amplification using primers TRO802/TRO803 and cDNA as matrix. Negative and positive controls were run using no RT cDNA reaction and genomic DNA as matrix, respectively.

SI Appendix, Supplementary Tables

# T1. Strains and plasmids.

| **Strain or plasmid** | **Description** | **Source or reference** |
| --- | --- | --- |
| **Strains** |  |  |
| *E. coli* DH5α-Z1 | Strain used for cloning | Expressys |
| *E. coli* MFD*pir* | Strain used for conjugation | (56) |
| *F. psychrophilum strain* OSU THCO2-90 | Wild type | (57) |
| *F. columnare* strain ATCC 49512 | Wild type | (58) |
| **Plasmids** |  |  |
| pCP*Gm*^r^ | *E. coli*–*F. psychrophilum* shuttle plasmid; ColE1 *ori* (pCP1 ori), Ap^r^ (Gm^r^) | (39) |
| pCP*Gm*^r^-P_less_-mCh | pCP*Gm*^r^ carrying a promoter less codon-adapted *mCherry* gene | This study |
| pCP*Gm*^r^-P*_remF_*-mCh | pCP*Gm*^r^-P_less_-mCh derivative with mCherry under the transcriptional control of *remF* promoter | This study |
| pCP*Gm*^r^-P*_remF-sm4*_*_-_mCh | pCP*Gm*^r^-P*_remF_*-mCh mutated in motif SM4 | This study |
| pCP*Gm*^r^-P*_remF-sm5*_*_-_mCh | pCP*Gm*^r^-P*_remF_*-mCh mutated in motif SM5 | This study |
| pCP*Gm*^r^-P*_remF-sm4*sm5*_*_-_mCh | pCP*Gm*^r^-P*_remF_*-mCh mutated in motif SM4 and SM5 | This study |
| pYT313 | Suicide vector carrying *sacB*; Ap^r^ (Em^r^) | (41) |
| pYT-*rfp18* | pYT313*-*derivative plasmid used to delete *rfp18* carrying *sacB* gene and upstream and downstream regions of *rfp18* | This study |

Antibiotic resistance markers: ampicillin, Ap^r^ is expressed in *E. coli* and erythromycin (Em^r^) and gentamicin (Gm^r^) in *F. psychrophilum*. Replication origin in parentheses is active in *F. psychrophilum*,

# T2. Oligonucleotides.

| **Name** | **Sequence 5’->3’** | **PCR Amplification** |
| --- | --- | --- |
| **Construction of pCP*Gm*^r^-P_less_-mCh** | |  |
| TRO454 | acatcgacaccaGAACTATCGCTTTGCGTCGTTC | pCP*Gm*^r^ |
| TRO455 | ATTATACAAATAGgaaaaggctcctgtttttgagc | pCP*Gm*^r^ |
| TRO456 | GCGATAGTTCtggtgtcgatgtaagaaatcaagtt | THC0290_0151 intrinsic terminator |
| TRO457 | *ctcgagactagtcttaagcgg*ccgctagccactgtttgctaagtgagctagac | THC0290_0151 intrinsic terminator |
| TRO458 | *ccgcttaagactagtctcgag*AAAAATTATGGTAAGCAAAGGAGAAGAAGATAA | Optimized mCherry gene |
| TRO459 | aacaggagccttttcCTATTTGTATAATTCATCCATTCCTCCG | Optimized mCherry gene |
| **Construction of pCP*Gm*^r^-P*_remF_*-mCh** | |  |
| TRO460 | CTCGAGAAAAATTATGGTAAGCAAAGG | pCP*Gm*^r^ P*_less_*-mCh |
| TRO461 | actagtcttaagcggccgcta | pCP*Gm*^r^ P*_less_*-mCh |
| TRO470 | tagcggccgcttaagactagtAGAATAAATTTTGCAATATGGTTTGT | *remF* promoter region (409 bp) |
| TRO609 | CCTTTGCTTACCATAATTTTTCTCGAGTTGTTTATAATTTAAATAATTTAAAGTTTTAAATGG | *remF* promoter region (409 bp) |
| **Construction of pCP*Gm*^r^-P*_remF-sm4*_***_-_**mCh (SM4 mutagenesis)** | | |
| TRO610 | AAATGTTTTT**TT**CACGATTTTA**TG**TGTGTAATTTAAAAGAAAGAAATTAG | pCP*Gm*^r^ P*_remF_*-mCh with sm4* |
| TRO611 | TAAATTACACA**CA**TAAAATCGTG**AA**AAAAACATTTTTAACGTAAAAAAAGACG | pCP*Gm*^r^ P*_remF_*-mCh with sm4* |
| **Construction of pCP*Gm*^r^-P*_remF-sm5*_***_-_**mCh (SM5 mutagenesis)** | | |
| TRO615 | GTTATTAGAAAC**GGTA**AGTTAGAATTTAAAGCGCTGTTTTTAGA | pCP*Gm*^r^ P*_remF_*-mCh with sm5* |
| TRO616 | CTTTAAATTCTAACT**TACC**GTTTCTAATAACTTTTTAATAAAAAAAATTAATT | pCP*Gm*^r^ P*_remF_*-mCh with sm5* |
| **Construction of pYT-*rfp18* (*rfp18* deletion)** | | |
| TRO186 | AAATGTGCGCGGAACCCCTA | pYT313 fragment |
| TRO187 | CGGCACATAACAAACAAATTGGC | pYT313 fragment |
| TRO721 | TAGGGGTTCCGCGCACATTTagaaggcatgatgcacga | Upstream region of *rfp18* |
| TRO722 | atgactgagtgtgtgggaattcgttaatttctaac | Upstream region of *rfp18* |
| TRO723 | ttcccacacactcagtcatttatttgactggg | Downstream region of *rfp18* |
| TRO724 | GCCAATTTGTTTGTTATGTGCCGagtgtcttttactttggcaaca | Downstream region of *rfp18* |
| TRO566 | CCCAGTCAAATAAATGACTGAGTTTTTATG | Internal fragment of *rfp18* |
| TRO565 | CAATTGAGGGGGTGGTTCCCT | Internal fragment of *rfp18* |
| TRO726 | cgctcacaaatagaagagtatct | Left-hand junction |
| TRO727 | aacatatctatcatgattgattctcc | Right-hand junction |
| **RT-qPCR** | | |
| TRO559 | CCCTAACGACGGAAGCGAAA | *fpp1* forward |
| TRO560 | GTAGTGCCGCCAACAGTAGT | *fpp1* reverse |
| TRO561 | CTGACGGAGCAAATGGCAAC | *fpp2* forward |
| TRO562 | CGACAAACTGTTTCCGCCAG | *fpp2* reverse |
| TRO563 | GCACCCGCTACACTAACCAA | *THC0290*_*0300* forward |
| TRO564 | GAACAGCCTCCGCGTCTTAT | *THC0290*_*0300* reverse |
| TRO745 | AACCCTTACAGCAGCGTGAG | *THC0290*_*1090* forward |
| TRO746 | AGATGTTGGAGCCATACCGC | *THC0290*_*1090* reverse |
| TRO800 | GAGGTTGCGGGACTGTTGTA | *THC0290*_*2338* forward |
| TRO801 | GCAACCCCACCTCCAGTAAA | *THC0290*_*2338* reverse |
| TRO487 | CAAACAACCGGAATTGGGGC | *gmd* forward |
| TRO488 | AAAGGGGTGTCCTCGGTTTG | *gmd* reverse |
| TRO489 | ATGGGAGCCGGAAAAACCAT | *parE* forward |
| TRO490 | CGAGTCGTACTTTCCTCCCG | *parE* reverse |
| **Detection of Rfp18 homolog in *F. columnare*** | | |
| TRO802 | TTGAGGGGTGGTTCCCTC | Forward |
| TRO803 | CCCAATCAGGAAATGACTGAGT | Reverse |

Underlined nucleotides correspond to regions which hybridize with targeted DNA. Mutated nucleotides are in bold. The sequence in italics corresponds to the multi-cloning site (NheI, NotI, AflII, SpeI, XhoI).

# T3. Description of the set of biological conditions analyzed by microarrays.

| **Subset** | **Label** | **Description of experimental conditions** |
| --- | --- | --- |
| Growth phases in TYES broth | T1 | Growth in TYES broth at 18°C to OD600 of 0.4 (exponential phase) |
|  | T2 | Growth in TYES broth at 18°C to OD600 of 1 (transition phase) |
|  | T3 | Growth in TYES broth at 18°C to OD600 of 1.2 (transition phase) |
|  | T4 | Growth in TYES broth at 18°C to OD600 of 1.3 (early stationary phase) |
|  | T5 | Growth in TYES broth at 18°C to OD600 of 1.7 (late stationary phase) |
|  | T2-FCS | Growth in TYES broth supplemented with 5% fetal calf serum at 18°C to OD600 of 1 (transition phase) |
| Colonies on agar | TYESA-FCS | Colonies grown at 18°C on TYES agar supplemented with 5% FCS |
|  | TYESA | Colonies grown at 18°C on TYES agar (reference) |
|  | TYESA-0.5 | Colonies grown at 18°C on TYES agar containing 2-times less nutrients |
|  | TYESA-0.2 | Colonies grown at 18°C on TYES agar containing 5-times less nutrients |
|  | TYESA-Blood | Colonies grown at 18°C on TYES agar plates with a 10% defibrinated horse blood-agar overlay |
|  | Blood | Colonies grown at 18°C on 10% defibrinated horse blood medium solidified with 1.5% agar |
|  | CS1 | Growth in TYES broth at 18°C to OD600 of 0.8, with no further treatment for 10 min (reference) |
| Oxidative stress | HP | Growth in TYES broth at 18°C to OD600 of 0.8, followed by addition of hydrogen peroxide (100 µM) for 10 min |
| Lysozyme stress | LYSO | Growth in TYES broth at 18°C to OD600 of 0.8, followed by addition of lysozyme (100 µg mL^-1^) for 10 min |
|  | CS2 | Growth in TYES broth under aeration to OD600 of 0.5, with no further treatment for 1 h (reference) |
| Amoxicillin | AMOX | Growth in TYES broth under aeration to OD600 of 0.5, followed by addition of amoxicillin (0.4 µg mL^-1^) for 1 h |
| Oxygen limitation | Anaer | Growth in TYES broth under aeration to OD600 of 0.5, followed by transfer in a completely filled 15 mL tube and static growth for 1 h |
|  | CS3 | Growth in TYES broth at 18°C to OD600 of 0.4 (reference) |
| Growth at 10°C | Temp10C | Growth in TYES broth at 10°C to OD600 of 0.4 |
| Growth at 4°C | Temp4C | Growth in TYES broth at 4°C to OD600 of 0.4 |
| Iron depletion | DIP | Growth in TYES broth supplemented 25 µM 2,2-dipyridyl at 18°C to OD600 of 0.4 |
| High osmolarity | NaCl | Growth in TYES broth supplemented 0.75% NaCl at 18°C to OD600 of 0.4 |
| Acid stress | CS4 | Growth in TYES broth under aeration to OD600 of 0.5, with no further treatment for 5 min (reference) |
|  | HCl | Growth in TYES broth under aeration to OD600 of 0.5, followed by addition of HCl to a final concentration of 0.75 mM for 5 min |
| Freshwater-living bacteria with and without rainbow trout | LAB | Growth in TYES broth to OD600 1 (reference) |
|  | Water | Growth in TYES broth to OD600 1, followed by 100-times dilution in dechlorinated fish tank water for 24 h |
|  | FishWater | Growth in TYES broth to OD600 1, followed by 100-times dilution in fish tank water containing juvenile rainbow trout for 24 h |
| Rainbow trout skin mucus exposure | EP | Growth in TYES broth to OD600 of 1, followed by centrifugation and resuspension of cells in 1% peptone water for 15 min (reference) |
|  | MUC | Growth in TYES broth to OD600 of 1, followed by centrifugation and resuspension of cells in a (1:1) mix of 1% peptone water and rainbow trout skin mucus for 15 min |
| Rainbow trout plasma exposure | EP2 | Growth in TYES broth to OD600 of 1, followed by cell washing and resuspension in 1% peptone water for 5 min (reference) |
|  | Plasma | Growth in TYES broth to OD600 of 1, followed by cell washing in 1% peptone water and resuspension in fresh rainbow trout plasma for 5 min |

Each subset of biological conditions corresponds to samples processed at the same time allowing to run differential expression analyses between conditions of each subset. For adaptation and stress response, the reference condition is specified. A detailed description of culture conditions and experimental procedures is available (**Table S1**).

# T4: Number of differentially expressed features across 23 pairwise comparisons of biological conditions.

| **Biological condition relative to reference** | **Test** | **Reference** | **Number of DE features** |
| --- | --- | --- | --- |
| Stationary phase of growth relative to log-phase | T5 | T1 | 1895 |
| Exposure to fish plasma | Plasma | EP2 | 1576 |
| Growth in tank water | Water | LAB | 1324 |
| Colony growth on blood relative to standard medium | Blood | TYESA | 1205 |
| Growth in tank water containing fish | FishWater | LAB | 1029 |
| Transition phase of growth | T3 | T1 | 1019 |
| Colony growth on blood | Blood | TYESA-0.2 | 641 |
| Colony growth on very low-nutrients medium | TYESA-0.2 | TYESA | 547 |
| Oxygen limitation | Anaer | CS2 | 526 |
| Colony growth on low-nutrients medium | TYESA-0.5 | TYESA | 500 |
| Peroxide stress | HP | CS1 | 409 |
| High osmotic pressure | NaCl | CS3 | 392 |
| Iron depletion | DIP | CS3 | 373 |
| Exposure to fish mucus | MUC | EP | 354 |
| Blood supplementation on solid medium | TYESA-Blood | TYESA | 192 |
| Growth at 4°C relative to 18°C | Temp4C | CS3 | 120 |
| Presence of fish | FishWater | Water | 76 |
| Log-phase of growth in serum supplemented medium | T2-FCS | T2 | 54 |
| Growth at 10°C relative to 18°C | Temp10C | CS3 | 54 |
| Colony growth on serum supplemented medium | TYESA-FCS | TYESA | 0 |
| Lysozyme stress | LYSO | CS1 | 0 |
| Amoxicillin exposure | AMOX | CS2 | 0 |
| Acid stress | HCl | CS4 | 0 |

Expression levels of 3853 features (CDSs, RFAM elements and TRs) was determined by cDNA hybridization on custom microarrays. DE features indicate those considered differentially expressed (log2-fold change > 1 or < -1, induced and repressed respectively; false-discovery rate of 5%). Lists of DE features are available for each pairwise comparison in **Table S5** and on the ***fpeb*** website.

Supplementary references

1. Mader U et al. *Staphylococcus aureus* Transcriptome Architecture: From Laboratory to Infection-Mimicking Conditions. PLoS genetics. 2016;12(4):e1005962.

2. Nicolas P et al. Condition-dependent transcriptome reveals high-level regulatory architecture in *Bacillus subtilis*. Science. 2012;335(6072):1103-6.

3. Abu-Jamous B, Kelly S. Clust: automatic extraction of optimal co-expressed gene clusters from gene expression data. Genome biology. 2018;19(1):172.

4. Staron A et al. The third pillar of bacterial signal transduction: classification of the extracytoplasmic function (ECF) sigma factor protein family. Mol Microbiol. 2009;74(3):557-81.

5. Barrios H, Valderrama B, Morett E. Compilation and analysis of sigma(54)-dependent promoter sequences. Nucleic acids research. 1999;27(22):4305-13.

6. Dahl SW et al. Carica papaya glutamine cyclotransferase belongs to a novel plant enzyme subfamily: cloning and characterization of the recombinant enzyme. Protein Expr Purif. 2000;20(1):27-36.

7. Sakamoto Y et al. Structural and mutational analyses of dipeptidyl peptidase 11 from *Porphyromonas gingivalis* reveal the molecular basis for strict substrate specificity. Sci Rep. 2015;5:11151.

8. Tsu BV, Saier MH, Jr. The LysE Superfamily of Transport Proteins Involved in Cell Physiology and Pathogenesis. PloS one. 2015;10(10):e0137184.

9. Kalvari I et al. Rfam 13.0: shifting to a genome-centric resource for non-coding RNA families. Nucleic acids research. 2018;46(D1):D335-D42.

10. Weinberg Z et al. Comparative genomics reveals 104 candidate structured RNAs from bacteria, archaea, and their metagenomes. Genome biology. 2010;11(3):R31.

11. Merino E, Jensen RA, Yanofsky C. Evolution of bacterial *trp* operons and their regulation. Curr Opin Microbiol. 2008;11(2):78-86.

12. Putzer H, Laalami S. Expression of Aminoacyl-tRNA Synthetases and Translation Factors. In: Bioscience L, editor. Madame Curie Bioscience Database [Internet]2000.

13. Kerpedjiev P, Hammer S, Hofacker IL. Forna (force-directed RNA): Simple and effective online RNA secondary structure diagrams. Bioinformatics. 2015;31(20):3377-9.

14. Wassarman KM. 6S RNA, a Global Regulator of Transcription. Microbiol Spectr. 2018;6(3).

15. Babicki S et al. Heatmapper: web-enabled heat mapping for all. Nucleic acids research. 2016;44(W1):W147-53.

16. Wright PR et al. CopraRNA and IntaRNA: predicting small RNA targets, networks and interaction domains. Nucleic acids research. 2014;42(Web Server issue):W119-23.

17. Duchaud E et al. Complete genome sequence of the fish pathogen *Flavobacterium psychrophilum*. Nature biotechnology. 2007;25(7):763-9.

18. Rochat T et al. Identification of a novel elastin-degrading enzyme from the fish pathogen *Flavobacterium psychrophilum*. Applied and environmental microbiology. 2019.

19. Barbier P et al. The type IX secretion system is required for virulence of the fish pathogen *Flavobacterium psychrophilum*. Applied and environmental microbiology. 2020.

20. Yang J, Tauschek M, Robins-Browne RM. Control of bacterial virulence by AraC-like regulators that respond to chemical signals. Trends Microbiol. 2011;19(3):128-35.

21. Lau ME, Loughman JA, Hunstad DA. YbcL of uropathogenic *Escherichia coli* suppresses transepithelial neutrophil migration. Infection and immunity. 2012;80(12):4123-32.

22. Fujita Y, Matsuoka H, Hirooka K. Regulation of fatty acid metabolism in bacteria. Mol Microbiol. 2007;66(4):829-39.

23. Subramanian C, Frank MW, Batte JL, Whaley SG, Rock CO. Oleate hydratase from *Staphylococcus aureus* protects against palmitoleic acid, the major antimicrobial fatty acid produced by mammalian skin. The Journal of biological chemistry. 2019;294(23):9285-94.

24. Volkov A et al. Myosin cross-reactive antigen of *Streptococcus pyogenes* M49 encodes a fatty acid double bond hydratase that plays a role in oleic acid detoxification and bacterial virulence. The Journal of biological chemistry. 2010;285(14):10353-61.

25. Du D et al. Multidrug efflux pumps: structure, function and regulation. Nature reviews Microbiology. 2018;16(9):523-39.

26. Hillion M, Antelmann H. Thiol-based redox switches in prokaryotes. Biol Chem. 2015;396(5):415-44.

27. Pitcher RS, Watmough NJ. The bacterial cytochrome *cbb3* oxidases. Biochim Biophys Acta. 2004;1655(1-3):388-99.

28. Alvarez B, Secades P, Prieto M, McBride MJ, Guijarro JA. A mutation in *Flavobacterium psychrophilum tlpB* inhibits gliding motility and induces biofilm formation. Applied and environmental microbiology. 2006;72(6):4044-53.

29. Olczak T, Sroka A, Potempa J, Olczak M. *Porphyromonas gingivalis* HmuY and HmuR: further characterization of a novel mechanism of heme utilization. Arch Microbiol. 2008;189(3):197-210.

30. Reddy KJ, Bullerjahn GS, Sherman DM, Sherman LA. Cloning, nucleotide sequence, and mutagenesis of a gene (*irpA*) involved in iron-deficient growth of the cyanobacterium *Synechococcus* sp. strain PCC7942. Journal of bacteriology. 1988;170(10):4466-76.

31. LaFrentz BR, LaPatra SE, Call DR, Wiens GD, Cain KD. Proteomic analysis of *Flavobacterium psychrophilum* cultured and in iron-limited media. Diseases of aquatic organisms. 2009;87(3):171-82.

32. Loimaranta V et al. Leucine-rich repeats of bacterial surface proteins serve as common pattern recognition motifs of human scavenger receptor gp340. The Journal of biological chemistry. 2009;284(28):18614-23.

33. Eshghi A et al. An extracellular *Leptospira interrogans* leucine-rich repeat protein binds human E- and VE-cadherins. Cell Microbiol. 2019;21(2):e12949.

34. Shannon P et al. Cytoscape: a software environment for integrated models of biomolecular interaction networks. Genome Res. 2003;13(11):2498-504.

35. Nakayama H, Tanaka K, Teramura N, Hattori S. Expression of collagenase in *Flavobacterium psychrophilum* isolated from cold-water disease-affected ayu (*Plecoglossus altivelis*). Biosci Biotechnol Biochem. 2016;80(1):135-44.

36. Sasagawa Y et al. Molecular cloning and sequence analysis of the gene encoding the collagenase from *Cytophaga* sp. L43-1 strain. Biosci Biotechnol Biochem. 1995;59(11):2068-73.

37. Gomez E, Alvarez B, Duchaud E, Guijarro JA. Development of a markerless deletion system for the fish-pathogenic bacterium *Flavobacterium psychrophilum*. PloS one. 2015;10(2):e0117969.

38. Pérez-Pascual D et al. Comparative analysis and mutation effects of *fpp2-fpp1* tandem genes encoding proteolytic extracellular enzymes of *Flavobacterium psychrophilum*. Microbiology (Reading, England). 2011;157:1196-204.

39. Pérez-Pascual D et al. More Than Gliding: Involvement of GldD and GldG in the Virulence of *Flavobacterium psychrophilum*. Frontiers in microbiology. 2017;8:2168.

40. Puigbo P, Guzman E, Romeu A, Garcia-Vallve S. OPTIMIZER: a web server for optimizing the codon usage of DNA sequences. Nucleic acids research. 2007;35(Web Server issue):W126-31.

41. Zhu Y et al. Genetic analyses unravel the crucial role of a horizontally acquired alginate lyase for brown algal biomass degradation by *Zobellia galactanivorans*. Environ Microbiol. 2017;19(6):2164-81.

42. Martin M. Cutadapt removes adapter sequences from high-throughput sequencing reads. EMBnetjournal. 2011;17(1):10-2.

43. Langmead B, Salzberg SL. Fast gapped-read alignment with Bowtie 2. Nature methods. 2012;9(4):357-9.

44. Li H et al. The Sequence Alignment/Map format and SAMtools. Bioinformatics. 2009;25(16):2078-9.

45. Rochat T et al. Complete Genome Sequence of *Flavobacterium psychrophilum* Strain OSU THCO2-90, Used for Functional Genetic Analysis. Genome announcements. 2017;5(8).

46. Mirauta B, Nicolas P, Richard H. Parseq: reconstruction of microbial transcription landscape from RNA-Seq read counts using state-space models. Bioinformatics. 2014;30(10):1409-16.

47. d'Aubenton Carafa Y, Brody E, Thermes C. Prediction of rho-independent *Escherichia coli* transcription terminators. A statistical analysis of their RNA stem-loop structures. J Mol Biol. 1990;216(4):835-58.

48. Ibrahim M et al. A genome-wide survey of short coding sequences in streptococci. Microbiology (Reading, England). 2007;153(Pt 11):3631-44.

49. Wernersson R, Nielsen HB. OligoWiz 2.0--integrating sequence feature annotation into the design of microarray probes. Nucleic acids research. 2005;33(Web Server issue):W611-5.

50. Rasmussen S, Nielsen HB, Jarmer H. The transcriptionally active regions in the genome of Bacillus subtilis. Mol Microbiol. 2009;73(6):1043-57.

51. Smyth GK. Linear models and empirical bayes methods for assessing differential expression in microarray experiments. Stat Appl Genet Mol Biol. 2004;3:Article3.

52. Camacho C et al. BLAST+: architecture and applications. BMC Bioinformatics. 2009;10:421.

53. Mars RA, Nicolas P, Denham EL, van Dijl JM. Regulatory RNAs in *Bacillus subtilis*: a Gram-Positive Perspective on Bacterial RNA-Mediated Regulation of Gene Expression. Microbiol Mol Biol Rev. 2016;80(4):1029-57.

54. Busch A, Richter AS, Backofen R. IntaRNA: efficient prediction of bacterial sRNA targets incorporating target site accessibility and seed regions. Bioinformatics. 2008;24(24):2849-56.

55. Andersen CL, Jensen JL, Orntoft TF. Normalization of real-time quantitative reverse transcription-PCR data: a model-based variance estimation approach to identify genes suited for normalization, applied to bladder and colon cancer data sets. Cancer Res. 2004;64(15):5245-50.

56. Ferrieres L et al. Silent mischief: bacteriophage Mu insertions contaminate products of *Escherichia coli* random mutagenesis performed using suicidal transposon delivery plasmids mobilized by broad-host-range RP4 conjugative machinery. Journal of bacteriology. 2010;192(24):6418-27.

57. Bertolini JM, Wakabayashi H, Watral VG, Whipple MJ, Rohovec JS. Electrophoretic detection of proteases from selected strains of *Flexibacter psychrophilus* and assesment of their variability. Journal of aquatic animal health. 1994;6:224-33.

58. Bernardet JF. *Flavobacteriaceae*. Bergey's Manual of Systematics of Archaea and Bacteria 2015.
